# Identification of the needs of individuals affected by COVID-19

**DOI:** 10.1038/s43856-024-00510-1

**Published:** 2024-05-09

**Authors:** Halina B. Stanley, Veronica Pereda-Campos, Marylou Mantel, Catherine Rouby, Christelle Daudé, Pierre-Emmanuel Aguera, Lesly Fornoni, Thomas Hummel, Susanne Weise, Coralie Mignot, Iordanis Konstantinidis, Konstantinos Garefis, Camille Ferdenzi, Denis Pierron, Moustafa Bensafi

**Affiliations:** 1 Université Claude Bernard Lyon 1, CNRS, INSERM, Centre de Recherche en Neurosciences de Lyon CRNL U1028 UMR5292, NEUROPOP, F-69500 Bron, France; 2 Équipe de Médecine Evolutive Faculté de chirurgie dentaire—UMR5288, CNRS/Université Paul-Sabatier Toulouse III, Toulouse, 31400 France; 3 Smell & Taste Clinic, Department of Otorhinlaryngology, Technische Universität Dresden, Dresden, Germany; 4 2nd Academic ORL Department, Papageorgiou Hospital, Aristotle University, Thessaloniki, Greece

**Keywords:** Lifestyle modification, Olfactory system

## Abstract

**Background:**

The optimal management of COVID-19 symptoms and their sequelae remains an important area of clinical research. Policy makers have little scientific data regarding the effects on the daily life of affected individuals and the identification of their needs. Such data are needed to inform effective care policy.

**Methods:**

We studied 639 people with COVID-19 resident in France via an online questionnaire. They reported their symptoms, effects on daily life, and resulting needs, with particular focus on olfaction.

**Results:**

The results indicate that a majority of participants viewed their symptoms as disabling, with symptoms affecting their physical and mental health, social and professional lives. 60% of the individuals reported having unmet medical, psychological and socio-professional support needs. Finally, affected individuals were concerned about the risk and invasiveness of possible treatments as shown by a preference for non-invasive intervention over surgery to cure anosmia.

**Conclusions:**

It is important that policy makers take these needs into consideration in order to assist affected individuals to regain a normal quality of life.

## Plain Language Summary

The impact of COVID-19 has been substantial, both on individuals’ health and on society. Information is needed to understand the biological mechanisms underlying the illness and to provide appropriate support for people affected. This study uses data from an online questionnaire of adults diagnosed with COVID-19 to characterize symptoms, understand their impact on peoples’ everyday lives, and determine the support that people need. Our over-arching analysis of symptoms experienced reveals that heart- and skin-related symptoms are linked to chronic illness, and symptoms related to the sense of smell may have a different underlying disease mechanism. Most respondents had a mild initial illness, but their symptoms were long-lasting and had a severe impact. Our findings show that sufferers need different kinds of support in order to regain a normal quality of life.

## Abstract (web summary)

Stanley et al. use data from an online questionnaire to investigate the impact of COVID-19 symptoms on peoples’ everyday lives. Many consider their symptoms, which affect their physical and mental health and social lives, so severe as to constitute a disability, and report having unmet medical, psychological, or socio-professional support needs.

## Introduction

By end-January 2023 there have been over 670 million cases of COVID-19 worldwide^1^ and the consequences of the COVID-19 crisis continue to emerge. Estimates of the incidence of long-term sequelae vary widely, but numbers are significant, of the order of at least 3% of those infected^2–6^ with long-term effects for perhaps 50–85% of those hospitalized^7–9^.

It is known that COVID-19 is associated with a large spectrum of symptoms ranging from the classic symptoms of flu, through gastro-intestinal, cardiac and renal, cognitive and olfactory and gustatory dysfunctions^10–14^. Three years after the start of the pandemic data are now emerging on long-lasting symptoms and sequelae even for patients with apparently mild initial illness^2,10,15^ and theoretical studies are underway to understand the mechanisms underlying the appearance of symptoms, such as molecular level investigations of infected cells^16,17^, the role of inflammation^18^ and potential drivers of long-COVID^19^.

Nevertheless, fundamental data remain incomplete and sometimes contradictory. COVID-19 is a multi-organ heterogeneous disease with many interacting factors: age, sex, comorbidities. Scientific knowledge is needed to help clinicians improve their diagnoses (https://www.who.int/teams/blueprint/covid-19) and also to help improve patients’ health by developing better adapted treatment strategies^20–22^.

Although studies are underway (e.g. ref. ^23^) characterization of COVID-19 symptoms (whether persistent or not) and their effect on quality of life, as well as identifying patients’ needs, has not yet been conducted with the level of detail that would allow a clear analysis of the situation.

To date, several questions remain regarding COVID-19 symptoms: how do they differ from one person—or group of persons—to another? What are their dynamics of appearance? How are they associated with each other? What differences are there between the acute and chronic phases? There is also a lack of actionable data on the impact these symptoms have on the quality of life of those affected. Are all these symptoms disabling? How do they affect psychological health? Dietary health? Social life and relationships? Working life? And above all, what are the needs of those affected in terms of medical, psychological and socio-professional support?

Today, policy makers and stakeholders have little scientific data on which to base an effective policy for caring for those affected and to define the resources needed (in terms of financial support, social services or care) to meet their needs, and to plan for future pandemics. If spending can be targeted effectively this could mitigate overall increases in health spending over the medium to long term^24^. The main aim of the present study is to provide characterization of a wide range of symptoms of COVID-19, their effects on quality of life and the needs of those affected. To achieve this goal, we conducted an online study involving a large sample of participants affected by symptoms several days, weeks or months after infection^25^. In this survey, we documented the presence of symptoms by category (flu-like, gastro-intestinal, cognitive & neurological, cutaneous & inflammatory, cardiac & renal, olfactory/gustatory, other), their onset and persistence, their effects on people’s daily lives and also identified people’s needs regarding these symptoms. In addition, to understand how inter-individual factors such as age and gender might explain the diversity in symptoms, effects on quality of life, and patients’ needs, we conducted systematic analyses for each of these three areas.

As a secondary aim, we explored the question of olfactory/gustatory loss in more detail, as this symptom affects several million people worldwide, a quarter to a third of whom continue to have some degree of measurable smell dysfunction for months after their infection^26–28^ and emerging information on a possible link between these COVID-19 olfactory symptoms and dementia is concerning^22^. Moreover, in addition to serious psychological and social effects^29–31^, anosmia (total loss of smell) and parosmia (distorted perception of smell) symptoms exacerbate the malnutrition documented post-COVID^32,33^. Finally, we collected verbatim responses in order to capture individual experiences and enrich our quantitative analysis.

We find a high prevalence of chronic symptoms, concerning a higher proportion of women than men. Olfactory, gustatory and flu-like symptoms were frequently experienced early in the illness, with cutaneous, inflammatory and cardiac symptoms often delayed. The data also show the severe impact flu-like and cognitive symptoms have on people’s everyday lives. We confirm the dietary impact of olfactory symptoms on sufferers and provide a detailed overview of the interventions desired by those affected by the long-term effects.

## Methods

### Selection of the participants

**Fig. 1:**
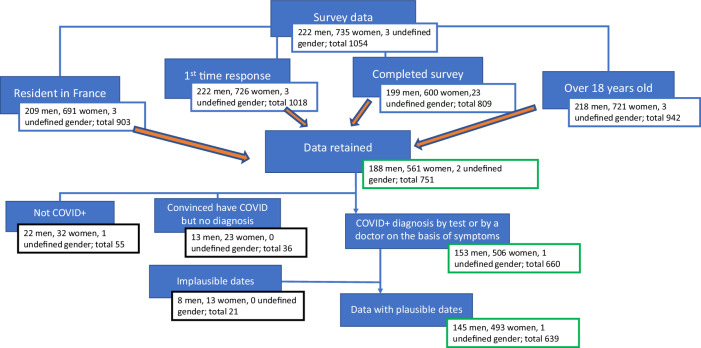
Overview of survey data and participant inclusion criteria. 751 people completed the whole survey who were over 18 years old, resident in France and completing the survey for the first time. Of these, 55 people who had no diagnosis and 36 people who were self-diagnosed were excluded. A further 21 participants were then excluded owing to implausible or missing diagnosis dates.

There were a total of 1054 responses in the study. However, we limited the analysis to adults (over 18 years old), resident in France, who were answering the questionnaire for the first time and completed the entire survey which left us 751 participants. We wanted to ensure, as much as reasonably possible, that symptoms arose from COVID-19 infection so only included participants diagnosed COVID+ either by an analytical test (PCR, lateral flow, blood test etc.) or by a doctor on the basis of their symptoms alone. This excluded 36 self-diagnosed participants and 55 participants without COVID-19. We also excluded 5 participants who did not record the date of their diagnosis, 3 participants who gave an implausible date (prior to the first diagnoses in France on the 24th January 2020^1,34^ and 13 participants who recorded a diagnosis date after their recovery date (see Fig. 1), ending with a final sample of 639 participants.

It should be noted that our analysis does not focus specifically on the “long COVID-19” population, although many participants fall into this category. This survey focused on the symptoms and needs of patients who have had COVID-19 infection, regardless of any subjective persistence of symptoms. Moreover, one can consider oneself cured of COVID-19, but still experience long-term sequelae and express needs. Thus, our analyses include all people who have been infected by Sars-Cov-2, whether they declare themselves cured or not.

### Experimental protocol

The descriptive cohort study was conducted between 15th July 2021 and 6th September 2022. It was approved by the Institutional Review Board of INSERM (IRB00003888, IORG0003254, FWA00005831) of the French Institute of medical research and health, under number 21-805. The study consisted of a cross-sectional online participatory survey. Participants had access to the questionnaire on the website https://project.crnl.fr/covid/. They learned about the survey through their internet searches and we also distributed the link to our scientific and academic network, and to the communication officers of the French institutions involved in the project. The first page of the website mentioned the objectives of the study and several pieces of information concerning the average time of completion and the way in which the answers are taken into account. It was also stated that the questionnaire is anonymous and that the record of their responses to the questionnaire does not contain any identifying information. Once this information was read, the participant was asked to provide their consent to participate in the survey. Afterwards, the participant was asked to answer several questions specifying: their age, gender, weight, height, socio-professional category, education level, place of residence, pregnancy (if female), smoking habits, if he/she has any chronic diseases and treatment, information about COVID-19 (diagnosis, hospitalization, treatment, vaccination), type and duration of symptoms (early, persistent), influence of these symptoms on psychological well-being, diet, social life, professional life, needs for medical follow-up, psychological follow-up, socio-professional follow-up and other needs and therapies. The details of the survey are given in Supplementary Notes 1.

### Characteristics of the sample of COVID-19+ patients surveyed

The principal characteristics of the survey participants are provided in Table 1. The people were mostly of working age (75th percentile is 52 years) and predominantly (77%) female. The majority experienced mild initial illness. Only 42 people were hospitalized (7%), of whom 25 received oxygen and 8 were treated in intensive care. 110 people (17%) were either occasional or regular smokers, which compares to 18.5% of people over 18 years old in France^35^. Further demographic information (on age and gender distribution, BMI, smoking habits, pregnancy status, participants’ illness & medication, vaccination status, hospitalization, temporal information on dates of diagnostic and survey completion) is detailed in Supplementary Notes 2. There were no significant gender related differences in the age distributions.

**Table 1:** Principal characteristics of the survey participants

Characteristics	Participants (*N* = 639)	
Age (years)	Mean ± SD: 43.0 ± 12.9 Median [range]: 44 [18–80]	
Gender	Male: 145 Female: 493 Undefined: 1	
BMI (kg/m^2^)	Mean ± SD: 23.9 ± 4.5 Median [range]: 23.2 [15.9–47.8]	
Socio-professional category	Agricultural workers: 2 Craftsmen, tradespeople & self-employed: 28 Intermediate professions: 54 Executives & professional: 232 Employees: 165 Non-professional: 8 Retired: 27 Without professional activity: 35	
Residence	Suburban: 137 Rural: 159 Urban: 343	
Pregnancy	No: 482 Possibly: 2 Yes: 9	
Smoking	Ex-smoker: 109 Non-smoker: 420 Occasional smoker: 41 Regular smoker: 69	
Chronic illness	No response: 6 No: 484 Yes: 149	
Illnesses mentioned	Asthma: 9 Hypertension: 11 Arthritis: 11 Allergies: 11	
Taking medication	No response: 12 No: 388 Yes: 239	
Hospitalized	No: 597	Yes: 42
Given oxygen (if hospitalized)	No: 17	Yes: 25
Intensive care (if hospitalized)	No: 34	Yes: 8
Treatment for COVID	No: 517	Yes: 122

The diagnosis dates range from the beginning of the pandemic (30th January 2020) to 29th August 2022 and correlate well with the waves of infection in France over this time^25^. The average number of days between a positive COVID diagnosis and completing the survey is 281 (see Supplementary Fig. 11). 54 of the 639 respondents completed the questionnaire less than two weeks after their COVID+ diagnosis.

Note that the diagnosis dates cover waves of infection with different dominant variants, with an under-representation of the delta & omicron variants (see ref. ^25^). However, we do not have sufficient confirmed diagnoses of the variant of infection (this was an open question) for us to be able to perform a statistical analysis comparing the different variants.

There is good geographical representation across metropolitan France, with a bias towards the Lyon, Toulouse and Paris areas. We have some over-representation of urban areas (Supplementary Notes 3).

### Data analysis

The data were extracted from the survey using the open-source software package Jamovi^36,37^ which was also used to extract some of the figures and all statistical tests.

We used descriptive statistics tools (percentage and 95% confidence intervals) to define the prevalence and dynamics of symptom appearance, to evaluate the disabling effect of symptoms on quality of life, and to identify the needs arising from all symptoms and to perform the in-depth analysis of specific needs for olfactory losses.

Generally, percentages were calculated relative to (i) the whole survey population (ii) the whole survey population by gender (iii) number reporting different symptom categories and (iv) number reporting presence of symptom by gender (note that the single individual who did not define their gender was excluded from all gender-based analyses).

Furthermore, 95% confidence intervals were calculated using the Effect Sizes and Confidence Intervals add-on module for Jamovi (esci) which uses the recommended method of Newcombe and Altman^38^(The code for this is available on github, lines 118–127 for a single proportion and lines 456–474 for the difference of two proportions https://github.com/rcalinjageman/esci/blob/master/R/estimateProportions.R).

P values for significance were calculated using jamovi with 2 sided tests for both proportions (for categorical data using χ^2^ and the z test for the difference in 2 proportions) and averages (for continuous data assuming equal variances). These are reported without corrections for multiple comparisons.

Finally, Pearson correlation coefficients r between different symptom categories were calculated by binarising the categorical data, mapping lack of symptom to zero and symptom experienced to one.

### Reporting summary

Further information on research design is available in the Nature Portfolio Reporting Summary linked to this article.

## Results

Here we first quantify the prevalence of the different symptoms for our survey population, together with the dynamics of onset & recovery and also examine symptom associations. We then examine responses related to the impact that these symptoms had on people’s everyday lives and the needs people expressed. We provide an in-depth analysis for olfactory loss.

Supplementary Notes 4 provides detailed information on the effect of gender and age differences on the prevalence of symptoms and their dynamics of onset and recovery. Supplementary Notes 5 provides detailed information for the subjects’ perception of their illness by symptom category, its disabling nature and their associated needs. Selected verbatim responses on the impact of each symptom, together with their translations into English, are also provided

### Characterization of the symptoms experienced

#### Prevalence of symptoms

**Fig. 2:**
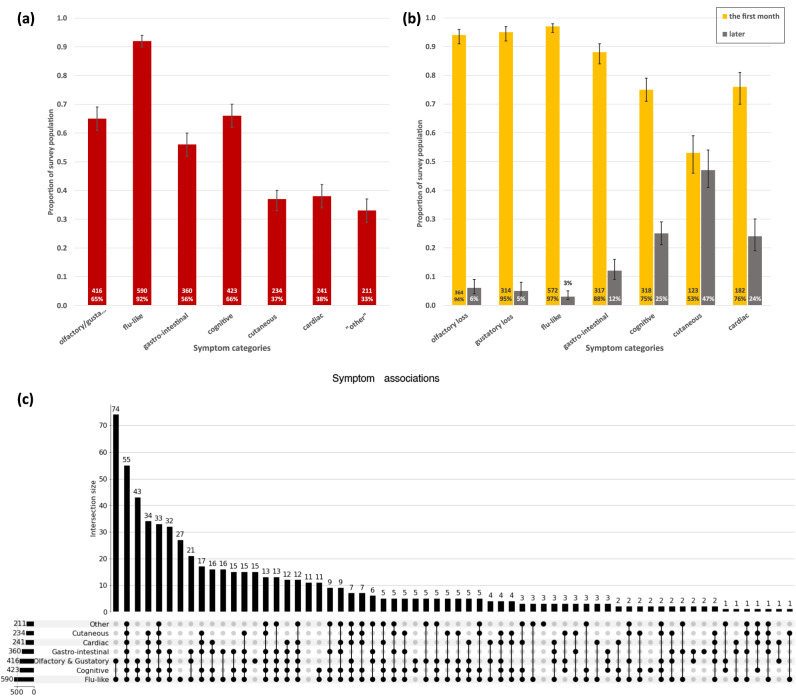
Symptoms suffered after infection with COVID-19. **a** Proportion of survey participants reporting symptoms by symptom category. Error bars are 95% confidence intervals. Inset in bars: Number of survey participants and percentage of survey population. **b** Proportion of survey participants by symptom onset (symptoms began in first month (yellow); later (gray)). Inset in bars: Number of survey participants and percentage by symptom category experienced. **c** Symptom associations. Upset plot illustrating the number of participants reporting different combinations of symptoms. The largest number of people (74) only experienced flu-like and olfactory symptoms.

Amongst our survey participants, 65% had olfactory/gustatory symptoms [95% CI 61–69%], 92% had flu-like symptoms [95% CI 90–94%], 66% [95% CI 62–70%] had cognitive, neurological or psychiatric, 56% [95% CI 52–60%] had gastro-intestinal symptoms, 38% [95% CI 34–42%] had cardiac or renal symptoms and 37% [95% CI 33–40%] had skin or inflammatory symptoms (see Fig. 2a). 33% [95% CI 29–37%] of people reported “other” symptoms. Responses for “other” symptoms were free and we note that symptoms were sometimes included here that could have been included elsewhere. People frequently mentioned extreme fatigue, breathlessness and cognitive symptoms such as tinnitus, vertigo, anxiety & headaches. Several people had issues with their eyesight or eyes. Women experienced menstrual changes and one man testicular pain. Some mentioned hair loss, pain in articulations, or reactivation of other viruses such as herpes simplex. Overall, 628 participants (98%) experienced at least one symptom and 55 participants (8.6%) experienced all the symptoms.

Regarding gender and age, women were more likely to experience symptoms than men in all symptom categories (*p* < 0.001). The average age of those reporting symptoms was higher than for those not reporting symptoms, except for olfactory/gustatory symptoms where there was no significant difference (Supplementary Fig. 17).

#### Dynamics of appearance of symptoms

Most participants experienced loss of olfaction and gustation, and flu-like symptoms within the first month of infection, however onset of cognitive, cutaneous & inflammatory, cardiac & renal symptoms was frequently delayed (see Fig. 2b). There were no significant gender or age differences for the onset of symptoms (Supplementary Figs. 18 and  19).

#### Associations between symptoms

**Fig. 3:**
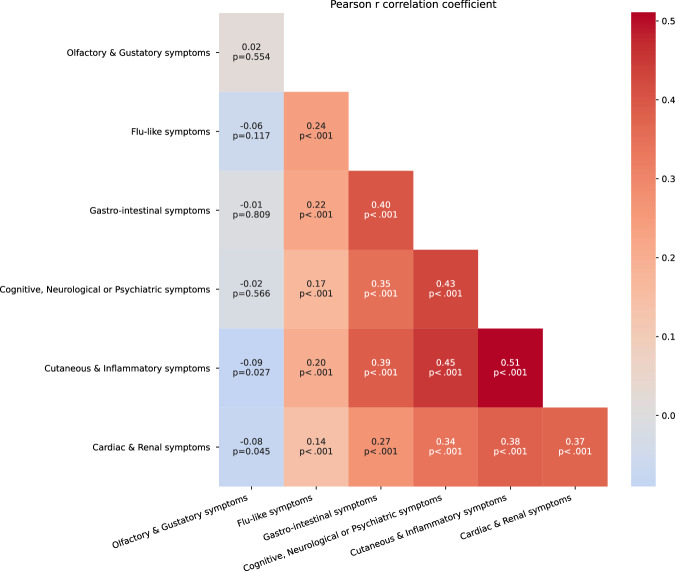
Correlation matrix for individual symptoms. Pearson’s r factor calculated from binary vectors, *p* < 0.05 interpreted as correlated, *p* < 0.01 very correlated. The heatbar corresponds to the Pearson r coefficients. The number of subjects experiencing each symptom category given in Fig. 2.

Figure 2c depicts the number of participants reporting different combinations of symptoms. The largest subset of people (11.6%) had only flu-like and olfactory/gustatory symptoms, and while 27 people only had flu-like symptoms, 15 had only olfactory/gustatory symptoms. We also note that 14% of the survey population suffered all symptoms, or all symptoms except “other” (that are not well defined). 139 people (21.8% of the survey population) reported flu-like and cardiac and cognitive and cutaneous and gastro-intestinal symptoms. We found that the incidence of these symptoms was correlated, but that there was no correlation, or an anti-correlation, with olfactory/gustatory symptoms (Fig. 3).

#### Persistence of symptoms

Overall, only 31% (200) of the 639 participants reported recovery at the time of completing the survey, with younger people and men more likely to report recovery than older people and women (Supplementary Notes 4). The median number of days between diagnosis and recovery (for the 200 people reporting recovery) was 18 (range 0–462). 439 people (69%) did not report recovery. However, 47 of these 439 people completed the survey less than 15 days after their diagnosis (when symptoms are to be expected) and 57 of the 439 people responded within 5 weeks. There were 363 people (57%) who did not report recovery more than 3 months after diagnosis. Only 53 people in our sample reported recovery after more than 35 days, and for 33 of these people it took between three and fifteen months after diagnosis.

For gender and age, more women than men reported persistent flu-like and gastro-intestinal symptoms (*p* < 0.001 and *p* = 0.004 respectively). The average age of those reporting persistent symptoms was higher than those without persistent symptoms for all symptom categories except gastro-intestinal and cutaneous & inflammatory symptoms (Supplementary Notes 4).

### Disabling effect of symptoms and impact on daily life

#### Disabling effect of symptoms

**Fig. 4:**
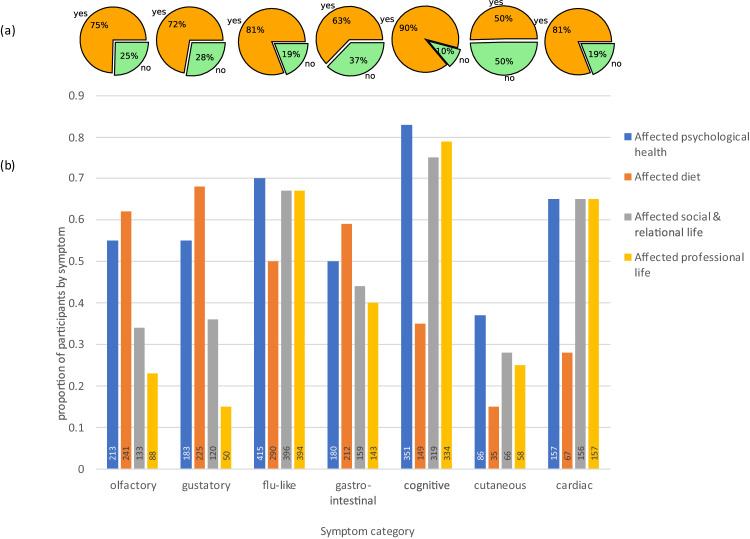
Impact of symptoms in everyday life by symptom category. **a** Percentage of people finding their symptoms handicapping (orange) or not (green) by symptom category. **b** Reported impact of symptoms on psychological health (blue), diet (orange), social & relational life (gray) and professional life (yellow) as a proportion of those experiencing each symptom category. The number of participants concerned is reported inside the bar.

A large proportion of survey participants found their symptoms a handicap in their everyday life (see Fig. 4a). Overall 75% of participants who reported olfactory loss found it handicapped them, 72% of those with gustatory loss, 81% of those with flu-like symptoms (which included headaches, fatigue and weakness), 63% of those with gastro-intestinal symptoms, who struggled with nausea and diarrhea, 90% of those with cognitive symptoms (mentioning depression, “brain fog”, memory and concentration problems) and 81% of those with cardiac or renal symptoms (who suffered tachycardia and chest pain among other symptoms) (see Supplementary Notes 5 and Supplementary Notes 6 for Selected verbatim responses on the impact of each symptom). More women than men found their flu-like, gastro-intestinal, cutaneous & inflammatory, and cardiac & renal symptoms handicapped them in their everyday life, but there was no gender difference for olfactory/gustatory and cognitive symptoms. There were no age-related differences in the perception of whether any of these symptoms were handicapping in everyday life.

#### Impact on daily life

Suffering any of the symptoms had an impact on the psychological health of over a third of respondents, rising to 70% of those with flu-like symptoms and 83% of those with cognitive symptoms (see Fig. 4b). Diet was affected for 50% or more of participants with olfactory, gustatory, flu-like or gastro-intestinal symptoms. Diet was also affected by cognitive, neurological and psychiatric type symptoms for over a third of people and by cardiac or renal symptoms for over a quarter of sufferers. Respondents’ social and professional lives were impacted for over two thirds of those with flu-like, cognitive, cardiac or renal symptoms. Regarding gender, although persistently more women than men reported impacts on their everyday lives this was rarely statistically significant. The small number of men concerned in many of these comparisons make conclusions on this unreliable (see Supplementary Notes 5). The only significant age-related difference was for flu-like symptoms, which younger people found affected their professional life more than older people (average ages 42.8 [41.8,43.9] years and 46.5 [43.0,50.0] years for those finding their flu-like symptoms affected their professional life or didn’t, respectively) (see Supplementary Notes 5).

### Identification of needs

**Fig. 5:**
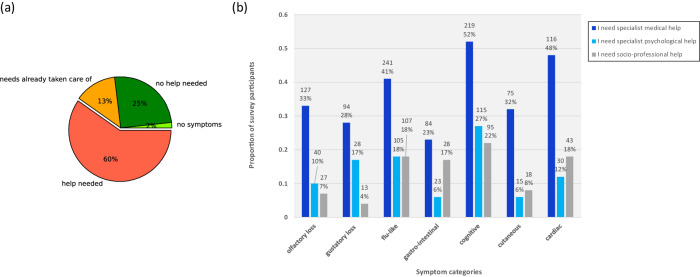
Stated needs of the survey participants for any of their symptoms. **a** Overall, 60% of the participants stated that they had needs that were not being addressed. Percentages are relative to the total survey population. N(no symptoms):11; N(no help needed):161; N(needs taken care of):85; N(help needed):382. **b** Overview of stated needs by symptom experienced. Proportions as a percentage of those experiencing each symptom. Specialist medical help required (dark blue), specialist psychological help required (light blue), socio-professional assistance required (gray).

Needs by type of symptoms. Overall, 60% of the survey population (382 people) expressed a need for some kind of help with managing their symptoms (see Fig. 5a), with a higher proportion of women than men (*p* = 0.002) (see Supplementary Notes 5). The average age of those seeking additional help was higher than the average age of those with no needs (*p* < 0.001) (see Supplementary Notes 5). There was no significant gender difference, or difference in the average age, between the group who reported that they had sufficient help with their symptoms already, and the group who reported they needed additional assistance (*p* = 0.155).

Of those that expressed a need for additional follow-up help, the majority, 58% (371 participants) wanted specialist medical help, 25% (158 participants) wanted help from a psychologist or psychiatrist, and 21% (137 participants) wanted socio-professional help.

Unsurprisingly, for those experiencing cognitive, neurological and psychiatric symptoms (migraines, forgetfulness, attention deficits, speech problems, confusion, anxiety, depression, sleep disorders etc.) there was also a significant desire for psychological and socio-professional support (see Fig. 5b). These people frequently cited an inability to work (see Supplementary Notes 5).

**Fig. 6:**
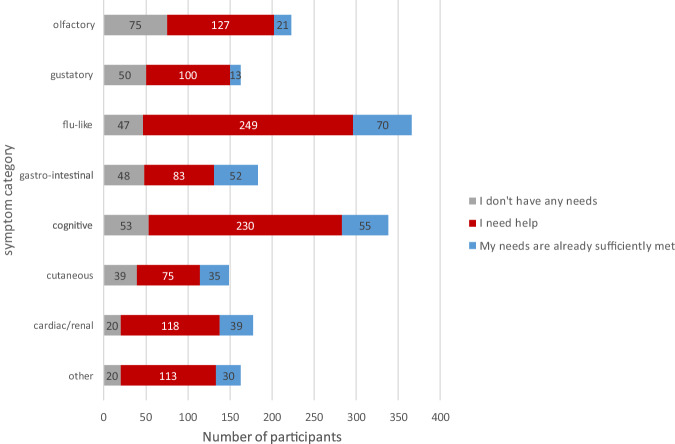
Stated needs for survey participants who had ongoing symptoms by symptom category. Gray: no help needed; red: I have needs that are not met; blue: I have needs for which I have sufficient help. The number of participants concerned is provided inside the bars.

The majority of the participants who reported that they had not yet recovered from their symptoms stated that they needed help, and only a small proportion said they had sufficient help already. Specifically, confining ourselves to participants who reported that they had not yet recovered from their symptoms, the percentage of people needing help was: 66% of people with olfactory symptoms, 69% of people with gustatory symptoms, 87% of people with flu-like symptoms, 73% of people with gastro-intestinal symptoms, 84% of people with cognitive symptoms, 73% of people with cutaneous symptoms, 89% of people with cardiac symptoms and 88% of people with “other” symptoms (see Fig. 6).

### Detailed analysis for a specific symptom: olfactory loss

**Fig. 7:**
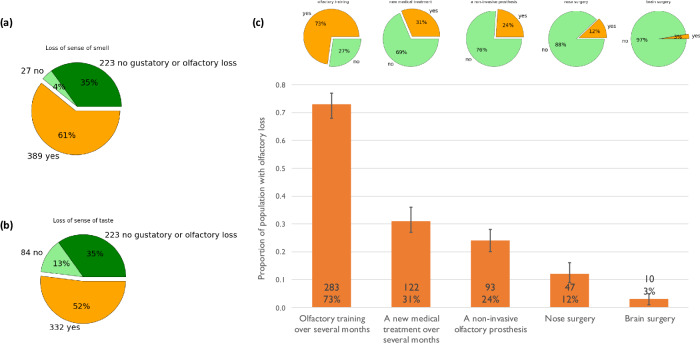
Analysis of olfactory loss. **a** Proportion of the 639 survey participants reporting loss of olfaction. **b** Proportion with loss of gustation. **c** Acceptable treatments for olfactory loss as a proportion of the survey population experiencing olfactory loss. Error bars are calculated 95% confidence intervals. Inset in bars: Number of participants and percentage of those with olfactory loss.

We conducted a detailed investigation for olfactory losses. Of the 416 survey participants reporting olfactory and/or gustatory loss, 389 (94%) experienced loss of olfaction and 80% (332) reported loss of sense of taste (see Fig. 7a, b). Of the participants losing olfaction 295 (76%) reported a total loss. This olfactory loss was frequently associated with changed odors (parosmia) (55%) and phantom odors (42%). These changed and phantom odors were almost invariably unpleasant with participants describing them as putrefaction, drains, sweat, burning, cigarette smoke, rotten eggs etc.

Of the individuals losing gustation 209 (63%) reported a total loss. This gustatory loss was associated with changed tastes in 63% of the participants and phantom tastes in 23% of the cases. People had difficulty describing their changed and phantom tastes; although many described metallic, burnt or rotten tastes, some said that food had a taste of perfume or “carrot juice tastes of flowers”. One person said everything tasted like toothpaste and another that courgette soup tasted of fish. Note that 10 of the 416 participants reporting olfactory and/or gustatory symptoms reported no loss of smell or of taste. It is unclear whether these participants experienced changed or phantom symptoms as if a participant did not report loss these questions were not asked. Two thirds of the participants with olfactory and/or gustatory symptoms provided verbatim comments, reflecting a high degree of distress amongst this population (see Supplementary Notes 5).

Survey participants reporting olfactory loss were also asked what potential interventions they would accept to restore their sense of smell. 73% (283 participants) would follow olfactory training over several months, 31% (122 participants) would choose a new medical treatment over several months, nearly a quarter were prepared to wear a non-invasive olfactory prosthesis and 12% (47 people) would accept nasal surgery. 10 people indicated acceptance of a prosthesis requiring invasive brain surgery (see Fig. 7c).

## Discussion

The objective of the present study was to characterize the symptoms and sequelae of COVID reported by affected individuals, as well as the impact that these symptoms have on their quality of life. In particular, our study aimed to identify the needs of the persons concerned in terms of medical, psychological and socio-professional support.

In terms of prevalence, we found that although over 93% of the people in our survey were not hospitalized, the proportion of people with symptoms was high (see Fig. 2a) and only 31% (200/639) reported recovery. We expect our data to suffer from self-selection bias so extrapolation of the prevalence of symptoms from our data to the general population cannot be rigorous, however the numbers of people concerned are high. The prevalence of different symptoms of COVID varies very widely in the literature^11,22,39–45^. Early in the pandemic reports were centered on symptoms experienced by those with more severe illness and may not reflect the statistics of the large population with milder initial illness. Prevalence also appears to vary with COVID-19 variant and in particular with the onset of the omicron variant. For example^46^, states that 52.7% of people experienced anosmia during the delta variant wave compared to only 16.7% during the omicron wave (for comparison we find 61% reporting olfactory loss with data mostly prior to the omicron wave). The estimates of the prevalence of asymptomatic cases also vary widely^47–49^ with vaccination also playing a role^50^; however this population is clearly under-represented in our data. Finally, the onset of cognitive, cardiac and cutaneous symptoms is frequently delayed so the prevalence of these symptoms may be under-reported in studies of acute illness.

Moreover, gender effects and age differences were observed. For gender, the women in our study were significantly more likely than men to have symptoms and took longer to recover. For age, we find that the average age of those reporting gastro-intestinal, cardiac, cutaneous or cognitive symptoms was greater than that of those without these symptoms. However, olfactory/gustatory symptoms have a different patient profile to these symptom categories since there was no statistical difference in the average age of those suffering olfactory and gustatory symptoms and those not (*p* = 0.164) (consistent with the results of Stankevice et al.^51^). Nevertheless, the average age of those with persistent olfactory loss (*p* = 0.013) was higher than that of those without, agreeing with the results of Makaronidis et al.^52^.

Regarding the dynamics of appearance of the symptoms, participants in our survey reported the early onset of olfactory/gustatory, flu-like and gastro-intestinal symptoms, agreeing with Kaye et al., Lechien et al.^53,54^ and Groff et al.^55^ but cognitive and cardiac/renal symptoms frequently began after the first month agreeing with Jason et al. and Davis et al.^56,57^. We find that the appearance of cutaneous and inflammatory symptoms is often delayed, although the work of Polly and Fernandez^58^ indicates that these conditions are heterogeneous. Davis et al.^59^ also made these global observations. Davis et al. and Apple et al.^57,60^ associated the delayed onset of neurological symptoms with younger people, but we find no statistically significant difference in age for this factor (*p* = 0.470). We also found no dependence on age for the onset of cardiac/renal symptoms (*p* = 0.356) but there was a tendency for those experiencing cutaneous symptoms in the first month to be younger than those with a later onset (*p* = 0.069). One question raised by these data is why different symptom categories have different onset dynamics. It is tempting to associate early onset symptoms (loss of smell, flu) with direct upper respiratory infection and the persistent cognitive impairment, late onset cardiac problems and skin lesions, alopecia, etc. with an immune response to the virus, perhaps with delayed effects over time. This hypothesis, which is rather speculative, deserves to be tested with an interdisciplinary approach combining neuro-sensory, medical and biological research via a longitudinal patient follow-up study.

Finally, regarding associations between symptoms, the literature often focuses on individual symptoms, but our study suggests that a broader analysis may reveal interesting patterns. First, we showed that almost all symptoms were correlated with each other. However, whilst olfactory and gustatory disorders were highly correlated with each other, these two types of disorders were not correlated with any other category of symptoms, suggesting different mechanisms underlying their genesis. We also found that there was a strong correlation (Pearson’s r = 0.51) between cardiac and cutaneous symptoms, and that those with symptoms in these categories were likely to have multiple symptoms and chronic disease. Finally, of those surveyed, the largest subgroup suffered only from flu and olfactory/gustatory symptoms, with the second largest group suffering from all categories of symptoms. To summarize, the symptomatology of COVID-19 should not be seen in a unidimensional way but through a pattern of symptoms that may be more or less prevalent and associated with each other, and with a specific appearance dynamic.

Although all symptom categories were classed as “handicapping” for a significant number of sufferers our data show that some are more disabling than others. For example, cognitive disorders (P(Yes) = 90% [87%, 93%]) are reported to be more disabling than skin disorders (50% [44%, 57%]) (see Fig. 4a). Furthermore, each type of symptom does not affect people’s quality of life in the same way. While smell disorders have a great impact on diet (62%) and psychological health (55%), cognitive disorders affect people’s professional life (79%) as well as their psychological health (83%), social and relational life (75%), with diet less affected (35%) (see Fig. 4b).

Overall, 60% of the participants declared that they needed support of various kinds, with only 25% declaring that they did not need support (and 13% that their needs were already taken care of). This inter-individual difference may be related to difficulties in accessing health care systems (e.g. distance, personal and financial resources etc.), a feeling of vulnerability to the disease that differs from one person to another, or the fact of being affected by very disabling symptoms. However, we find a substantial healthcare burden, which even may be under-estimated: time will tell. Of those still suffering cardiac/renal symptoms at the time of completing the survey only 11% said they had no need of help; the numbers for cutaneous, cognitive, gastro-intestinal, flu-like and “other” were 26%, 16%, 26%, 13% and 12% respectively.

The symptom categories with highest needs were flu-like (with participants reporting headaches, fatigue, muscle and joint pain) and cognitive (migraines, forgetfulness, lack of attention, anxiety, sleep disorders etc.) as these symptoms concerned the highest number of participants, but all symptom categories were problematic. Cognitive disorders (e.g., difficulty in concentrating or with memory) affect social activities and leave people unable to work^59^; participants were also impacted by sleep disorders. Selected verbatim responses can be found in Supplementary Notes 6.

The free responses to our survey also highlight the severe disruption to daily life that olfactory loss can produce^61–66^. Anosmia significantly affects the hedonic perception of food, reducing people’s desire to prepare and eat food, which causes weight gain, weight loss and nutritional deficits^29^. Epidemiological studies link nutrition with psychological wellbeing^67–69^ and anosmia with depression^70,71^ and generally reduced emotional well-being^72,73^. COVID-19 associated olfactory loss is not very different from other post-viral olfactory loss in terms of quality of life^74^. Although roughly a third of people with olfactory or gustatory symptoms said they needed no help, of those wanting help and still experiencing symptoms only 14.2% (21/148) and 11.5% (13/113) respectively said their needs were met (see Fig. 6). It is concerning that for people resident in France, which has an excellent healthcare system^75^, most need is not met.

In our study we also set out to evaluate the technologies that people affected by anosmia were willing to accept as treatment. We found that olfactory training was the most acceptable method (70%), probably because it was considered less invasive, less expensive and less risky. On the other hand, brain surgery was rarely selected as an option (although 3% of people said they would accept it). It is interesting that 24% of the participants considered the use of a non-invasive prosthesis a possible treatment. A non-invasive olfactory prosthesis is probably considered a less risky treatment than brain surgery, the latter being possibly associated in people’s minds with unfounded efficacy in olfaction, risks and uncertainties and a longer recovery time^76,77^. The similar acceptability of medication and non-invasive prostheses is interesting, however the well-known disconnect between intention and action means that this result needs corroboration^78^.

To summarize, despite the fact that over 93% of our survey population initially had a relatively mild illness, without a need for hospitalization, symptoms were found to be long-lasting and to have a severe impact. People reported dietary problems, pain and inability to work and predominantly requested medical intervention. Over all symptom categories, we note that medical support is sought nearly two to three times more than psychological and/or socio-professional support. Medical support is important in managing the evolution of immediate and severe persistent symptoms and can also help manage co-morbidities. However, although only 20% of the people requested psychological or socio-professional follow-up, it is important not to discount this need, as we may hypothesize that participants did not choose this option owing to embarrassment^79^, or other response bias. In future work it would be interesting to determine if the need for psychological support is linked to the duration of the symptoms or to people’s uncertainty as to their evolution. We note that verbatim comments we have collected show that persistent symptoms affect the ability of people to lead a normal social and professional life, and we link this to the requests for socio-professional support. We believe that the economic aspect is also an important parameter to be considered and would like to see future work examining the impact of a potential reduction in affected persons’ working hours or even loss of employment. It is possible that the need for psychological or work-related support is under-estimated, or may increase over time. Finally, the focus on the needs of people who have lost their sense of smell also provides information on an important dimension: the notion of risk and invasiveness. These two notions are clearly integrated in people’s choices and it is important for researchers and policy makers to take these concerns into account when research projects or governmental measures related to these needs are, or will be, put in place.

Although our study has provided interesting insights into the symptoms and impact of COVID, we note that, in common with any random survey, our data are limited to those people who chose to respond and to complete a long questionnaire. Our survey population has a preponderance of women and a large number of people with “long COVID” (this appears typical for this kind of survey, for example the online survey of Davis et al.^59^ contained 78.7% women and for >91% of the people recovery time exceeded 35 weeks. The survey of Ferdenzi et al.^65^ had a gender bias of 78% women and that of Bousquet et al.^66^ 82% women). We also over-represent urban, educated individuals and exclude those with no internet access. These are selection biases for which no good statistical correction can be made (e.g. ref. ^80^). We can assume a selection bias towards people motivated to seek assistance with symptoms that are problematic for them. There is also the possibility that people who chose to respond are especially health sensitive. The large proportion of the survey population needing help with their symptoms may not, therefore, be reflected in the general population. Moreover, our data are entirely subjective with no external analytical control. We appreciate that we are collecting subjective information based on people’s individual perceptions and that these perceptions may be different for different populations or change with time; nevertheless such “expressed need” is fundamental information for policy makers to take into account.

Our selection criteria exclude self-diagnosed individuals. It is possible that self-diagnosed people may have additional barriers towards accessing care compared to those with a diagnosis of COVID-19. On the other hand, we do include individuals diagnosed COVID+ on the basis of their symptoms alone. This is necessary given the limited testing available at the beginning of the pandemic, but we may include people whose symptoms are not caused by COVID-19. Generally in terms of symptoms we rely on self-assessment with no external analytical control. For example, it is known that people are relatively poor at evaluating their olfactory and gustatory deficits; people often believe they have a deficit when objective testing shows that they are normal, or conversely remain unaware of their real deficits^64^. It may also be the case that people did not correctly identify gustatory loss, as people often confuse this with olfactory loss^81,82^ although recent work^83^ did confirm loss of taste associated with COVID-19 using the “GCCR Smell and Taste check” test (see also ref. ^27^). A further limitation is that “Other” symptoms are not defined. At the end of the survey participants were simply asked to describe symptoms that they had not mentioned in preceding questions. Some participants included symptoms, such as breathlessness and fatigue (which had been listed in the previous description of “Flu-like symptoms”) or anxiety (which had been listed under “cognitive symptoms”). The symptoms described as “other” are very heterogeneous.

Finally, although as commented above, the prevalence of symptoms varies with COVID-19 variant (e.g. ref. ^46^) we do not have a large enough sample size to be able to evaluate this factor.

Nevertheless, taken as a whole, our data do have features that give confidence in the information provided. They show that the average age of hospitalized people is greater than that of un-hospitalized people and that the average age of those with symptoms is higher than the average age of those without, correlating with known information relating to the vulnerability of people to COVID-19 increasing with age. The greater vulnerability of women towards developing chronic effects (which is what we implicitly measure via self-selection bias) is also consistent with recent studies^5,84^. The dates reported by the participants are consistent with the different waves of infection in France^85^. Finally the geographical distribution of the participants correlates well with official government indicators^86,87^.

Another result that needs to be discussed is that a significant proportion of participants declared themselves cured of COVID-19, yet later described a number of persistent symptoms (80/200). These responses, which at first sight seem counterintuitive, are undoubtedly linked to the fact that declaring oneself cured of COVID-19 depends in part on subjective factors, on the individual perception of each person. This individual perception, which our data show to be variable from one person to another, is possibly constructed on the basis of the appreciation of the severity of the persistent symptoms, or of the feeling that people have still not recovered their initial state of health. In fact, all of this suggests that there is no simple definition of who is considered cured or not cured.

## Conclusions

The participants in our study experienced a relatively mild initial illness, but were nevertheless highly symptomatic with a large number finding their symptoms handicapping. The presence of symptoms of different types was correlated, with the notable exception of those of olfactory/gustatory nature, which appear to have a different patient profile. Flu-like and olfactory/gustatory symptoms invariably began early in the illness, but for many people cognitive, cutaneous & inflammatory, and cardiac symptoms began after the first month. Women were significantly more likely than men to have symptoms and a higher proportion of women than men reported they needed additional help. In terms of age, the average age of those with symptoms (of all types except olfactory/gustatory) was higher than that of those without and the average age of those seeking additional help was higher than the average age of those with no needs.

Our study shows symptoms severely affect both physical and mental health together with social and professional interactions. We highlight here the often neglected impact of olfactory loss on sufferers’ nutrition, mood, safety & social interactions, for these people improved access to olfactory training is needed, as few medical solutions exist. It is important that policy makers act to enable affected people regain a normal quality of life. Multidisciplinary support is needed to help manage the physical, emotional and social challenges of the disease: people predominantly ask for specialist medical help, for which improved access is needed, but patients with anxiety & depression need help managing their mental health and those unable to work normally need adequate financial support and help with managing their professional challenges. There is also a need for raising awareness in the general population by fighting against fake news, supporting scientific research, and supporting caregivers and families.

Finally, the magnitude of the health burden suggested by this study is of concern, but the true impact in the general population remains uncertain. The inherent selection biases of an online survey may overestimate, or underestimate, the problem. To extrapolate to the general population, we need results from random representative samples, data which are hard to obtain given the heterogeneous nature of the disease and (ideally) the need for clinical examinations. Nevertheless, given the scale of the problem already emerging, we feel this should be a priority.

### Supplementary information

Supplementary Information

Reporting Summary

## Data Availability

The source data used for all analysis described here has been deposited on the open access database zenodo in csv format^88^ together with a text readme file, a pdf with the survey questionnaire and three descriptive json files. We also provide a description of these data^89^.

## References

[1] WHO COVID-19 Dashboard. *World Health Organisation* https://covid19.who.int/info (2020).

[2] Al-Aly Z, Xie Y, Bowe B (2021). High-dimensional characterization of post-acute sequelae of COVID-19. Nature.

[3] Perlis RH (2022). Prevalence and Correlates of Long COVID Symptoms Among US Adults. JAMA Netw. Open.

[4] Sudre CH (2021). Attributes and predictors of long COVID. Nat. Med..

[5] Landry M (2023). Postacute Sequelae of SARS-CoV-2 in University Setting. Emerg Infect. Dis..

[6] Tan, B. K. J. et al. Prognosis and persistence of smell and taste dysfunction in patients with covid-19: Meta-analysis with parametric cure modelling of recovery curves. *BMJ*10.1136/bmj-2021-069503 (2022). 10.1136/bmj-2021-069503 PMC9326326 35896188

[7] Pavli A, Theodoridou M, Maltezou HC (2021). Post-COVID Syndrome: Incidence, Clinical Spectrum, and Challenges for Primary Healthcare Professionals. Arch. Med. Res..

[8] Sugiyama A (2022). Long COVID occurrence in COVID-19 survivors. Sci. Rep..

[9] Nasserie T, Hittle M, Goodman SN (2021). Assessment of the Frequency and Variety of Persistent Symptoms Among Patients With COVID-19: A Systematic Review. JAMA Netw. Open.

[10] Xie Y, Xu E, Bowe B, Al-Aly Z (2022). Long-term cardiovascular outcomes of COVID-19. Nat. Med..

[11] Grant MC (2020). The prevalence of symptoms in 24,410 adults infected by the novel coronavirus (SARS-CoV-2; COVID-19): A systematic review and meta-analysis of 148 studies from 9 countries. PLoS One.

[12] Buckley BJR (2021). Prevalence and clinical outcomes of myocarditis and pericarditis in 718,365 COVID-19 patients. Eur. J. Clin. Invest..

[13] Xie Y, Xu E, Al-Aly Z (2022). Risks of mental health outcomes in people with covid-19: cohort study. BMJ.

[14] Boscolo-Rizzo P (2023). Psychophysical assessment of olfactory and gustatory function in post-mild COVID-19 patients: A matched case-control study with 2-year follow-up. Int. Forum Allergy Rhinol..

[15] Xu E, Xie Y, Al-Aly Z (2022). Long-term neurologic outcomes of COVID-19. Nat. Med..

[16] Melms JC (2021). A molecular single-cell lung atlas of lethal COVID-19. Nature.

[17] Delorey TM (2021). COVID-19 tissue atlases reveal SARS-CoV-2 pathology and cellular targets. Nature.

[18] Eberhardt N (2023). SARS-CoV-2 infection triggers pro-atherogenic inflammatory responses in human coronary vessels. Nat. Cardiovasc. Res..

[19] Frere JJ (2023). SARS-CoV-2 infection in hamsters and humans results in lasting and unique systemic perturbations after recovery. Sci. Transl Med..

[20] Faghy MA (2022). COVID-19 patients require multi-disciplinary rehabilitation approaches to address persisting symptom profiles and restore pre-COVID quality of life. Expert Rev. Respir. Med..

[21] Briggs A, Vassall A (2021). Count the cost of disability caused by COVID-19. Nature.

[22] Kay LM (2022). COVID-19 and olfactory dysfunction: a looming wave of dementia?. J. Neurophysiol..

[23] Steinmetz A (2023). The Greifswald Post COVID Rehabilitation Study and Research (PoCoRe)–Study Design, Characteristics and Evaluation Tools. J. Clin. Med..

[24] OECD. Investing in health systems to protect society and boost the economy: Priority investments and order-of-magnitude cost estimates (abridged version). *OECD Policy Responses to Coronavirus (COVID-19)* https://www.oecd.org/coronavirus/policy-responses/investing-in-health-systems-to-protect-society-and-boost-the-economy-priority-investments-and-order-of-magnitude-cost-estimates-abridged-version-94ba313a/ (2022).

[25] Stanley, H. B. & Bensafi, M. A data set of symptoms and needs of individuals affected by COVID-19. *Nat. Sci. Data* **11**, 122 (2024). 10.1038/s41597-024-02961-6 PMC10808179 38267450

[26] Bussiere N (2022). Persisting chemosensory impairments in 366 healthcare workers following COVID-19: an 11-month follow-up. Chem. Senses.

[27] Doty RL (2022). Olfactory dysfunction in COVID-19: pathology and long-term implications for brain health. Trends Mol. Med..

[28] Boscolo-Rizzo P (2021). High prevalence of long-term olfactory, gustatory, and chemesthesis dysfunction in post-COVID-19 patients: a matched case-control study with one-year follow-up using a comprehensive psychophysical evaluation. Rhinology.

[29] Watson DLB (2021). Altered smell and taste: Anosmia, parosmia and the impact of long Covid-19. PLoS One.

[30] Connelly, D. ‘My whole world changed’: The repulsive smells that linger for months post COVID. *Pharmaceutical J.* **308**, No. 7960 (2022)

[31] Tan NKW (2022). The burden of prolonged smell and taste loss in covid-19. BMJ.

[32] Barrea, L. et al. Dietary Recommendations for Post-COVID-19 Syndrome. *Nutrients* **14**, 10.3390/nu14061305 (2022). 10.3390/nu14061305 PMC8954128 35334962

[33] Ferrulli A, Senesi P, Terruzzi I, Luzi L (2022). Eating Habits and Body Weight Changes Induced by Variation in Smell and Taste in Patients with Previous SARS-CoV-2 Infection. Nutrients.

[34] Bernard Stoecklin S (2020). First cases of coronavirus disease 2019 (COVID-19) in France: surveillance, investigations and control measures, January 2020. Euro. Surveill..

[35] Eurostat. Daily smokers of cigarettes by sex, age and educational attainment level. https://ec.europa.eu/eurostat/databrowser/view/HLTH_EHIS_SK3E__custom_6000386/default/table?lang=en&page=time:2019 (2019).

[36] Fox, J. & Weisberg, S. *Companion to Applied Regression* 3rd edn (Sage, 2020).

[37] The_jamovi_project. jamovi version 2.2.5 [computer software]. https://www.jamovi.org (2021).

[38] Newcombe, R. & Altman, D. in *Statistics with Confidence* Ch. 6 (BMJ Books, 2017).

[39] Lin L (2020). Gastrointestinal symptoms of 95 cases with SARS-CoV-2 infection. Gut.

[40] Cagnazzo F (2021). Neurological manifestations of patients infected with the SARS-CoV-2: a systematic review of the literature. J. Neurol..

[41] Caruso D (2021). Post-acute sequelae of COVID-19 pneumonia: Six-month chest CT follow-up. Radiology.

[42] Struyf, T. et al. Signs and symptoms to determine if a patient presenting in primary care or hospital outpatient settings has COVID-19. *Cochrane Database Syst. Rev.* **2022**, CD013665 (2022). 10.1002/14651858.CD013665.pub3 PMC9121352 35593186

[43] Vaira LA (2023). COVID-19 related persistent olfactory disorders represent an unprecedented challenge. Am. J. Otolaryngol..

[44] Cooper KW (2020). COVID-19 and the Chemical Senses: Supporting Players Take Center Stage. Neuron.

[45] Zhao Y-H, Zhao L, Yang X-C, Wang P (2021). Cardiovascular complications of SARS-CoV-2 infection (COVID-19): a systematic review and meta-analysis. Rev. Cardiovasc. Med..

[46] Menni C (2022). Symptom prevalence, duration, and risk of hospital admission in individuals infected with SARS-CoV-2 during periods of omicron and delta variant dominance: a prospective observational study from the ZOE COVID Study. Lancet.

[47] Kalish H (2021). Undiagnosed SARS-CoV-2 seropositivity during the first 6 months of the COVID-19 pandemic in the United States. Sci. Transl. Med..

[48] Vihta, K. D. et al. Omicron-associated changes in SARS-CoV-2 symptoms in the United Kingdom. *Clin. Infect. Dis.*10.1093/cid/ciac613 (2022). 10.1093/cid/ciac613 PMC9384604 35917440

[49] Byambasuren O (2020). Estimating the extent of asymptomatic COVID-19 and its potential for community transmission: Systematic review and meta-analysis. J. Assoc. Med. Microbiol. Infectious Dis. Can..

[50] Tande AJ (2022). Impact of the Coronavirus Disease 2019 (COVID-19) Vaccine on Asymptomatic Infection Among Patients Undergoing Preprocedural COVID-19 Molecular Screening. Clin. Infect. Dis..

[51] Stankevice D, Fjaeldstad AW, Agergaard J, Ovesen T (2023). Long-Term COVID-19 Smell and Taste Disorders Differ Significantly from Other Post-Infectious Cases. Laryngoscope.

[52] Makaronidis J (2021). Distorted chemosensory perception and female sex associate with persistent smell and/or taste loss in people with SARS-CoV-2 antibodies: a community based cohort study investigating clinical course and resolution of acute smell and/or taste loss in peopl. BMC Infect. Dis..

[53] Kaye R, Chang CWD, Kazahaya K, Brereton J, Denneny JC (2020). COVID-19 Anosmia Reporting Tool: Initial Findings. Otolaryngol. Head Neck Surg..

[54] Lechien JR (2020). Olfactory and gustatory dysfunctions as a clinical presentation of mild-to-moderate forms of the coronavirus disease (COVID-19): a multicenter European study. Eur. Arch. Oto Rhino Laryngol..

[55] Groff A (2021). Gastrointestinal Manifestations of COVID-19: A Review of What We Know. Ochsner. J..

[56] Jason LA (2021). COVID-19 symptoms over time: comparing long-haulers to ME/CFS. Fatigue.

[57] Davis, H. E., McCorkell, L., Vogel, J. M. & Topol, E. J. Long COVID: major findings, mechanisms and recommendations. *Nat. Rev. Microbiol.*10.1038/s41579-022-00846-2 (2023) 10.1038/s41579-022-00846-2 PMC9839201 36639608

[58] Polly S, Fernandez AP (2022). Common skin signs of COVID-19 in adults: An update. Cleve Clin. J. Med..

[59] Davis HE (2021). Characterizing long COVID in an international cohort: 7 months of symptoms and their impact. EClinicalMedicine.

[60] Apple AC (2022). Risk factors and abnormal cerebrospinal fluid associate with cognitive symptoms after mild COVID-19. Ann. Clin. Transl. Neurol..

[61] Croy I, Nordin S, Hummel T (2014). Olfactory Disorders and Quality of Life—An Updated Review. Chem. Senses.

[62] Keller A, Malaspina D (2013). Hidden consequences of olfactory dysfunction: a patient report series. BMC Ear. Nose Throat Disord..

[63] Manesse C (2017). Dysosmia-Associated Changes in Eating Behavior. Chemosens. Percept..

[64] Manesse, C. et al. The prevalence of olfactory deficits and their effects on eating behavior from childhood to old age: A large-scale study in the French population. *Food Qual. Prefer.* **93**, 104273 (2021).

[65] Ferdenzi C (2021). Recovery From COVID-19-Related Olfactory Disorders and Quality of Life: Insights From an Observational Online Study. Chem. Senses.

[66] Bousquet C, Bouchoucha K, Bensafi M, Ferdenzi C (2023). Phantom smells: a prevalent COVID-19 symptom that progressively sets in. Eur. Arch. Otorhinolaryngol.

[67] Lassale C (2019). Healthy dietary indices and risk of depressive outcomes: asystematic review and meta-analysis of observational studies. Mol. Psychiatry.

[68] Salari-Moghaddam A, Saneei P, Larijani B, Esmaillzadeh A (2019). Glycemic index, glycemic load, and depression: a systematic review and meta-analysis. Eur. J. Clin. Nutr..

[69] Kaiser, A., Schaefer, S. M., Behrendt, I., Eichner, G. & Fasshauer, M. Association of sugar intake from different sources with incident depression in the prospective cohort of UK Biobank participants. *Eur. J. Nutr.*10.1007/s00394-022-03022-7 (2022). 10.1007/s00394-022-03022-7 PMC9941260 36205767

[70] Yom-Tov E, Lekkas D, Jacobson NC (2021). Association of COVID19-induced anosmia and ageusia with depression and suicidal ideation. J. Affect. Disord. Rep..

[71] Croy I, Hummel T (2017). Olfaction as a marker for depression. J. Neurol..

[72] Mahmut MK, Croy I (2019). The role of body odors and olfactory ability in the initiation, maintenance and breakdown of romantic relationships—A review. Physiol. Behav..

[73] Lübke KT, Pause BM (2015). Always follow your nose: The functional significance of social chemosignals in human reproduction and survival. Horm. Behav..

[74] Otte MS (2023). Impact of COVID-19-Mediated Olfactory Loss on Quality of Life. ORL J. Otorhinolaryngol. Relat. Spec..

[75] OECD. State of Health in the EU: Country Health Profiles France: Country Health Profile 2021. https://read.oecd-ilibrary.org/social-issues-migration-health/france-country-health-profile-2021_7d668926-en#page1 (2021).

[76] Besser G, Liu DT, Renner B, Hummel T, Mueller CA (2019). Olfactory implant: Demand for a future treatment option in patients with olfactory dysfunction. Laryngoscope.

[77] Pinger M (2021). Perceived utility of electronic noses in patients with loss of smell. Eur. Arch. Oto Rhino Laryngol..

[78] Sheeran P, Webb TL, Gollwitzer PM (2005). The Interplay Between Goal Intentions and Implementation Intentions. Pers. Soc. Psychol. Bull..

[79] Bohns VK, Flynn FJ (2010). “Why didn’t you just ask?” Underestimating the discomfort of help-seeking. J Exp. Soc. Psychol..

[80] Schnell R, Noack M, Torregroza S (2017). Differences in general health of internet users and non-users and implications for the use of web surveys. Surv. Res. Methods.

[81] Deems DA (1991). Smell and Taste Disorders, A Study of 750 Patients From the University of Pennsylvania Smell and Taste Center. Arch. Otolaryngol. Head Neck Surg..

[82] Doty RL (2018). Measurement of chemosensory function. World J. Otorhinolaryngol. Head Neck. Surg..

[83] Ohla K (2022). A follow-up on quantitative and qualitative olfactory dysfunction and other symptoms in patients recovering from COVID-19 smell loss. Rhinol. J..

[84] Fernández-de-las-Peñas, C. et al. Female Sex Is a Risk Factor Associated with Long-Term Post-COVID Related-Symptoms but Not with COVID-19 Symptoms: The LONG-COVID-EXP-CM Multicenter Study. *J. Clin. Med.* **11**, 10.3390/jcm11020413 (2022). 10.3390/jcm11020413 PMC8778106 35054108

[85] Dong E, Du H, Gardner L (2020). An interactive web-based dashboard to track COVID-19 in real time. Lancet Infect. Dis..

[86] de Bellefon, M.-P. (PSAR A. territoriale-I., Eusebio, P. ((PSAR A. territoriale-I., Forest, J. ((PSAR A. territoriale-I. & Warnod, R. ((PSAR A. territoriale-I. 38% de la population française vit dans une commune densément peuplée. *Statistiques et études* https://www.insee.fr/fr/statistiques/4252859 (2019).

[87] INSEE. Population statistics of France. https://www.insee.fr/fr/statistiques/1893198 (2022).

[88] Bensafi, M. & Stanley, H. B. COVID-19—Symptoms—Impact on quality of life and needs of affected people. *Zenodo*10.5281/zenodo.7920303 (2023).

[89] Stanley HB, Bensafi M (2024). A data set of symptoms and needs of individuals affected by COVID-19. Sci Data.

