# Supplementary material for: Identification of the needs of individuals affected by COVID-19
Source: Commun Med (Lond). 2024 May 9;4:83. doi: 10.1038/s43856-024-00510-1 (PMC11082167; doi:10.1038/s43856-024-00510-1)
Supplement: Supplementary file 1 — Supplementary Information [file 43856_2024_510_MOESM1_ESM.pdf]

Identification of the needs of individuals affected by COVID-19

Supplementary materials

Halina B. Stanley[1*], Veronica Pereda-Campos[2], Marylou Mantel[1,2,3], Catherine Rouby[1], Christelle Daudé[1], Pierre-Emmanuel Aguera[1], Lesly Fornoni[1], Thomas Hummel[4], Susanne Weise[4], Coralie Mignot[4], Iordanis Konstantinidis[5], Konstantinos Garefis[5], Camille Ferdenzi[1], Denis Pierron[2], Moustafa Bensafi[1*]

[1]Université Claude Bernard Lyon 1, CNRS, INSERM, Centre de Recherche en Neurosciences de Lyon CRNL U1028 UMR5292, NEUROPOP, F-69500, Bron, France

[2]Équipe de Médecine Evolutive Faculté de chirurgie dentaire - UMR5288; CNRS/Université Paul-Sabatier Toulouse III, Toulouse, 31400, France

[3]Université Reims Champagne-Ardennes, Laboratoire C2S, 57 rue Pierre Taittinger, BP 30, 51571 Reims cedex, France

[4]Smell & Taste Clinic, Department of Otorhinolaryngology, Technische Universität Dresden, Dresden, Germany

[5]2nd Academic ORL Department, Papageorgiou Hospital, Aristotle University, Thessaloniki, Greece

* Corresponding authors: correspondence to halina.stanley@cnrs.fr or moustafa.bensafi@cnrs.fr

Table of contents

SUPPLEMENTARY NOTES 1 2

SUPPLEMENTARY NOTES 2 (AGE, GENDER, BMI, SMOKING HABITS, PREGNANCY, ILLNESS, MEDICATION, VACCINATION, HOSPITALIZATION) 15

SUPPLEMENTARY NOTES 3 (GEOGRAPHICAL INFORMATION) 26

SUPPLEMENTARY NOTES 4 (SYMPTOMS AS A FUNCTION OF GENDER, AGE; DYNAMICS) 29

SUPPLEMENTARY NOTES 5 (PERCEPTIONS) 42

SUPPLEMENTARY NOTES 6 (SUMMARY INFORMATION & VERBATIM REPONSES) 52

SUPPLEMENTARY REFERENCES 66

Supplementary Notes 1

Online survey on Covid-19 :
Analysis of the persistence of symptoms and their effects on everyday life and the identification of people's needs.

This questionnaire was designed by the Centre for Research in Neuroscience in Lyon

\- - - - - - - - - -

<u>Information for Participants</u>

Today more than 100 million people in the world have been infected with COVID-19. Knowledge of the disease has greatly developed since the beginning of the pandemic in early 2020 and today. In addition to the symptoms identified at the start of the pandemic we now know of additional symptoms that are sometimes persistent. Researchers propose a questionnaire to:

1/ understand better how the different symptoms (« flu-like », «olfactory and/or gustatory loss », « gastro-intestinal», « cognitive, neurological et psychiatric », « cutaneous and inflammatory », « cardiac and renal», other) influence the well-being, social life, diet and the professional life of the the people affected

2/ identify the needs of those affected.

If you have had Covid-19, answering this survey will allow us to understand better the effects of the disease on people's daily lives and needs..

Thank you in advance for the time you are taking to answer this.

Your answers will be of great help.

This questionnaire was approved by ethical review board of Inserm (IRB00003888) on the 13th April 2021

Note : Completing this questionnaire takes about 10 to 15 minutes.
Take care, your responses will only be taken into account if you answer in one sole sitting: any unfinished questionnaire will not be taken into account.

For any difficulty, please contact us via this email : *contact email*

If you want to go back, use the "back" button located below the questionnaire.
This questionnaire works on the browsers Chrome, Edge, Safari, Firefox and Explorer.

\- - - - - - - - - -

Note on privacy protection
This questionnaire is aNoymous.
The recording of your answers to this questionnaire does not contain any information allowing you to be identified.

\- - - - - - - - - -

<u>Consent to participate</u>

Please tick this box if you agree to participate in the survey.: *

\- - - - - - - - - -

Start the survey <u>here</u>

1. Preamble

Is this the first time you are completing this questionnaire?

- o Yes
- o No

2. Sociodemographic information

2.1. You are :

- o A woman
- o A man
- o I prefer not to answer

2.2. How old are you :

___ ans

2.3. How tall are you :

___ cm

2.4. What is your weight:

___ kg

2.5. What is your socio-professional category : ___ [dropdown list]

2.6. What is your level of education : ___ [dropdown list]

2.7. Do you live in France ?

- o Yes
- o No

If Yes, give your department of residence : ___ [dropdown list]

If Yes, indicate if you live :

- o in an urbain area
- o in a peri-urban area
- o in a rural area

If No, give your country of residence : ___ [dropdown list]

2.8. Are you pregnant ? (if a woman or " I prefer not to answer")

- o Yes I am pregnant
- o I may be pregnant
- o No

2.9. Do you smoke ?

- o Yes every day
- o Yes occasionally
- o I am an ex-smoker
- o No

3. Information Covid-19, Quality of life and Needs

3.1. Do you have one or more chronic illnesses ?

- o Yes
- o No

- o I prefer not to answer

  If Yes: Describe your chronic illness or illnesses : ___

3.2. Are you currently taking one or more medical treatments?

- o Yes
- o No
- o I prefer not to answer

If Yes: Indicate the drug or drugs : ___

3.3. Concerning your Covid-19 diagnosis

- 3.3.1. Have you been diagnosed positive for Covid-19 by mouth/nasal swab (e.g. PCR test) or by another method (e.g. x-ray, blood test, saliva etc.)?YES - NO (go to 3.3.2)
  - If Yes:
    - Give the diagnosis date : ____ [date]

      Today,,have you recovered ?
      - o Yes : Indicate the date of end of illness: ____ [date]
      - o No
    - Have you been sequenced for what are called “new variants” of the coronavirus (British, South African, Japanese, Brazilian, Others…):
      - YES - NO – I don’t know
      - If Yes, Have you been identified as a carrier of one of these variants?
        - o Yes, which : _____
        - o No
- 3.3.2. Have you been diagnosed with Covid-19 by a doctor on the basis of your symptoms alone?

  YES - NO (go to 3.3.3)
  - If Yes: Give the diagnosis date : ____ [date]

    Today,,have you recovered ? ?
    - o Yes : Indicate the date of end of illness: ____ [date]
    - o No
- 3.3.3. Are you convinced that you have had Covid-19 without having been formally diagnosed?

  YES - NO (End of questionnaire)
  - If Yes: Give the date of your self-diagnosis : ____ [date]

    Today,,have you recovered ?
    - o Yes : Indicate the date of end of illness: ____ [date]
    - o No

3.4. Have you been hospitalized for Covid-19? YES - NO

- o If Yes :

  How long was your hospitalization ? ____

  Did you receive oxygen ? YES - NO

  Were you in intensive care? YES - NO

  If Yes : How long did you stay in intensive care ? _____

3.5. Have you followed treatment for Covid-19? YES - NO

If Yes : What ? _____

3.6. Have you been vaccinated against Covid-19? YES - NO

If Yes :

Have you received a single dose of vaccine? YES/NO

Have you received more than one dose of vaccine? YES/NO
Which vaccine ? _____[dropdown list] Pfizer, Astrazeneca...I don’t know

3.7. Have you had the following symptoms? (*For this page, pages 3.8 to 3.14 open once the person has answered all the points of 3.7)*

- « olfactory and gustatory » symptoms of Covid_19 (Loss of smell and/or taste): YES - NO (If Yes, open 3.8)
- « flu-like » symptoms of Covid-19 (Fever, Chills or dizziness, Cough, Sore throat, Tiredness, Shortness of breath, Muscle weakness, Muscle pain (ache) or joint pain, Headache): YES - NO (If Yes, open 3.9)
- « gastro-intestinal » symptoms of Covid-19 (Difficulty swallowing, Difficulty eating/drinking, Loss of appetite, Nausea or vomiting, Diarrhea): YES - NO (If Yes, open 3.10)
- « cognitive, neurological and psychiatric » symptoms of Covid-19 (Migraines, Memory disorders, Attention disorders, Speech disorders, Altered consciousness, Confusion, Serious neurological disorders, Irritability, Anxiety disorders, Depression, Sleep disorders): YES - NO (If Yes, open 3.11)
- « cutaneous and inflammatory » symptoms of Covid-19 (Rash, Itching, Hair loss, Loss of teeth, Conjunctivitis (red eyes)): YES - NO (If Yes, open 3.12)
- « cardiac and renal » symptoms of Covid-19 (Kidney problems, Heart problems, Chest pain): YES - NO (If Yes, open 3.13)
- Other symptoms: YES - NO
  If Yes:
    - Can you describe it or them? ____
    - Open 3.14

3.8. Concerning the « olfactory and gustatory » symptoms of Covid_19 (Loss of smell and/or taste)

You have lost:

- The sense of smell (eg. loss of olfactory sensations of food, gas/smoke, perfumes, flowers …) : YES - NO
- The sense of taste (eg. loss of salty, sweet, sour, bitter tastes) : YES - NO

<u>If Yes to Olfaction</u> :

- The loss was: partial – total
- When did this symptom appear?: the first month or later
- Do you still have this symptom? YES – NO
  If NO, after how much time did this symptom disappear: less than one month, between 1 and 3 months, between 3 and 6 months, more than 6 months
- Is, or was, this loss also associated with phantom odors : YES – NO
    - If Yes, describe these phantom odors : ____
- Is, or was, this loss associated with a distorted perception of odors (that is to say that the odors no longer smell the same as before) : YES – NO
- Whether it is still present or not, has this loss of smell been disabling in your daily life? YES – NO
  If Yes : Does it influence or has it influenced
    - your psychological well-being: YES - NO - Specify if you wish : ___
    - your diet: YES - NO - Specify if you wish : ___
    - your social and relational life: YES - NO - Specify if you wish : ___
    - your professional life: YES - NO - Specify if you wish : ___
        - What is your profession ? ___
    - something else : YES - NO - Specify if you wish : ___

- Can you describe your needs with regard to these symptoms and their effects on your daily life? ?
    - I don't need anything for these symptoms (if ticked -> 3.9)
    - My needs are already sufficiently taken care of (if ticked -> 3.9)
    - I need follow-up
        - by a medical specialist: YES - NO - Specify if you wish : ___
        - psychological (psychologist, psychiatrist) : YES - NO - Specify if you wish : ___
        - socio-professional (social worker, occupational health doctor) : YES - NO - Specify if you wish : ___
        - Autre: ___
- Of the following possible treatments that would allow you to detect odors, are you seriously willing to try:
    - A new drug treatment over several months : Yes – No
    - Olfactory training over several months : Yes – No
    - Nose surgery : Yes – No
    - An olfactory prosthesis requiring brain surgery : Yes – No
    - An olfactory prosthesis that can be fitted non-invasively : Yes – No
    - A different therapy : ____
    - In what everyday situation would these possible therapies be most useful to you? Specify if you wish : ___

<u>If Yes to Taste</u> :

- The loss was: partial – total
- When did this symptom appear?: the first month or later
- Do you still have this symptom? YES – NO
  If No, after how much time did this symptom disappear: less than one month, between 1 and 3 months, between 3 and 6 months, more than 6 months
- Is, or was, this loss also associated with phantom tastes : YES – NO
    - If Yes, describe these phantom tastes : ____
- Is, or was, this loss associated with a distorted perception of tastes (that is to say that things no longer taste the same as before) : YES – NO
- Whether it is still present or not, has this loss of taste been disabling in your daily life? YES – NO
  If Yes : Does it influence or has it influenced
    - your psychological well-being: YES - NO - Specify if you wish : ___
    - your diet: YES - NO - Specify if you wish : ___
    - your social and relational life: YES - NO - Specify if you wish : ___
    - your professional life: YES - NO - Specify if you wish : ___
        - Quelle est votre profession ? ___
    - something else : YES - NO - Specify if you wish : ___
- Can you describe your needs with regard to these symptoms and their effects on your daily life? ?
    - I don't need anything for these symptoms (if ticked -> 3.9)
    - My needs are already sufficiently taken care of (if ticked -> 3.9)
    - I need follow-up
        - by a medical specialist: YES - NO - Specify if you wish : ___
        - psychological (psychologist, psychiatrist) : YES - NO - Specify if you wish : ___
        - socio-professionnel (social worker, occupational health doctor) : YES - NO - Specify if you wish : ___
        - Other: ___

3.9. Concerning the « flu-like» symptoms of Covid-19 (Fever, Chills or dizziness, Cough, Sore throat, Tiredness, Shortness of breath, Muscle weakness, Muscle pain (ache) or joint pain, Headache)

- When did the first of these symptoms appear?: the first month or later
- Is at least one of these symptoms still present ? YES - NO
  - If Yes :
    Which one or ones: ______
  - If No, after how much time did these symptoms disappear (Less than one month, between 1 and 3 months, between 3 and 6 months, more than 6 months)
- Whether they are still present or have disappeared, have these symptoms been disabling in your daily life?? YES - NO
  - If Yes :
    Which one or ones: ______
  - If Yes : Does it influence or has it influenced
    - your psychological well-being: YES - NO
    - your diet: YES - NO
    - your social and relational life: YES - NO
    - your professional life: YES - NO
    - something else : YES - NO
- Can you describe your needs with regard to these symptoms and their effects on your daily life? ?
  - I don't need anything for these symptoms (if ticked -> 3.10)
  - My needs are already sufficiently taken care of (if ticked -> 3.10)
  - I need follow-up
    - by a medical specialist: YES - NO
    - psychological (psychologist, psychiatrist) : YES - NO
    - socio-professional (social worker, occupational health doctor) : YES - NO
    - Other: ___

3.10. Concerning the « gastro-intestinal » symptoms of Covid-19 (Difficulty swallowing, Difficulty eating/drinking, Loss of appetite, Nausea or vomiting, Diarrhea)
Idem 3.9

3.11. Concerning the « cognitive, neurological and psychiatric » symptoms of Covid-19 (Migraines, Memory disorders, Attention disorders, Speech disorders, Altered consciousness, Confusion, Serious neurological disorders, Irritability, Anxiety disorders, Depression, Sleep disorders)
Idem 3.9

3.12. « cutaneous and inflammatory » symptoms of Covid-19 (Rash, Itching, Hair loss, Loss of teeth, Conjunctivitis (red eyes))
Idem 3.9

3.13. Concerning the « cardiac and renal » symptoms of Covid-19 (Kidney problems, Heart problems, Chest pain)
Idem 3.9

3.14. Concerning the the other symptoms that you have described but are not mentioned in the preceding questions
Idem 3.9

<u>The questionnaire as published online in French :</u>

Enquête participative sur la Covid-19 :
Analyse de la persistance des symptômes et de leurs effets sur le quotidien
***et* Identification des besoins des personnes**

Ce questionnaire a été conçu par le laboratoire Centre de recherche en neurosciences de Lyon

- - - - - - - - - -

<u>Information aux participants</u>

A ce jour, plus de 100 millions de personnes ont été touchées par la Covid-19 dans le monde. La connaissance de la maladie a largement évolué entre la déclaration de la pandémie début 2020, et aujourd'hui. Aux symptômes connus en début de pandémie sont venus s'ajouter de nouveaux symptômes parfois persistants. Des chercheurs proposent un questionnaire pour :
1/ mieux comprendre comment les différents symptômes (symptômes « type grippal », « perte d'odorat et/ou de goût », symptômes « gastro-intestinaux », « cognitifs, neurologiques et psychiatriques », « cutanés et inflammatoires », « cardiaques et rénaux », autres) influencent le bien-être, la vie sociale, l'alimentation et la vie professionnelle des personnes touchées
2/ identifier les besoins des personnes touchées.
Si vous avez eu la Covid-19, répondre à cette enquête nous permettra de mieux comprendre les effets de la maladie sur le quotidien et les besoins des personnes.
Merci d'avance du temps que vous consacrerez à y répondre.
Vos réponses seront d'une aide précieuse.
Ce questionnaire a reçu un avis favorable du comité d'évaluation éthique de l'Inserm (IRB00003888) le 13 avril 2021

Note : la passation de ce questionnaire dure environ 10 à 15 minutes.
Attention, vos réponses seront prises en compte si vous répondez à tout en une seule fois : tout questionnaire non terminé ne sera pas pris en compte.

Pour toute difficulté, merci de nous contacter à l'adresse suivante : *email-contact*

Si vous souhaitez revenir en arrière, utilisez le bouton "précédent" situé en dessous du questionnaire.
Ce questionnaire fonctionne sur les navigateurs Chrome, Edge, Safari, Firefox et Explorer.

- - - - - - - - - -

Remarque sur la protection de la vie privée
Ce questionnaire est anonyme.
L'enregistrement de vos réponses à ce questionnaire ne contient aucune information permettant de vous identifier.

- - - - - - - - - -

<u>Consentement à participer</u>

Merci de cocher cette case si vous consentez à participer à l'enquête : *

- - - - - - - - - -

Débuter l'enquête <u>ici</u>

1. Préambule

Est-ce la première fois que vous remplissez ce questionnaire ?

- o Oui
- o Non

2. Informations sociodémographiques

2.1. Vous êtes :

- o Une femme
- o Un homme
- o Je ne souhaite pas répondre

2.2. Quel est votre âge :

___ ans

2.3. Quelle est votre taille :

___ cm

2.4. Quel est votre poids :

___ kg

2.5. Quelle est votre catégorie socio-professionnelle : ___ [liste déroulante]

2.6. Quel est votre niveau d'éducation : ___ [liste déroulante]

2.7. Résidez-vous en France ?

- o Oui
- o Non

Si oui, indiquez votre département d'habitation : ___ [liste déroulante]

Si oui, indiquez si vous habitez :

- o en zone urbaine
- o en zone péri-urbaine
- o en zone rurale

Si non, indiquez votre pays d'habitation : ___ [liste déroulante]

2.8. Êtes-vous enceinte ? (si femme ou "je ne souhaite pas repondre")

- o Oui je suis enceinte
- o Je suis susceptible d'être enceinte
- o Non

2.9. Fumez-vous ?

- o Oui quotidiennement
- o Oui occasionnellement
- o Je suis un ancien fumeur
- o Non

3. Informations Covid-19, Qualité de vie et Besoins

3.1. Avez-vous une ou des maladies chroniques ?

- o Oui
- o Non

o Je ne souhaite pas répondre

Si Oui: Décrivez votre ou vos maladies chroniques : ___

3.2. Suivez-vous actuellement un ou des traitements médicamenteux ?

- o Oui
- o Non
- o Je ne souhaite pas répondre

Si Oui: Indiquez le ou les médicaments : ___

3.3. Concernant votre diagnostic à la Covid-19

- 3.3.1. Avez-vous été diagnostiqué(e) positif(ve) à la Covid-19 par prélèvement buccal/nasal (ex. test PCR) ou par une autre méthode (ex. radiographie, prise de sang, salive etc.) ?

OUI - NON (passer à 3.3.2)

- Si OUI:
  - Indiquez la date du diagnostic : ____ [date]

    Aujourd'hui, êtes-vous guérie(e) ?
    - o Oui : Indiquez la date de fin de la maladie : ____ [date]
    - o Non
  - Avez-vous été séquencé(e) pour ce qu'on appelle les « nouveaux variants » du coronavirus (Britannique, Afrique du sud, Japon, Brésil, Autres…):
    - OUI - NON - Je ne sais pas
    - Si OUI, Avez-vous été identifié(e) comme porteur(se) d'un de ces variants ?
      - o Oui, lequel : _____
      - o Non

- 3.3.2. Avez-vous été diagnostiqué(e) positif(ve) à la Covid-19 sur la base de vos symptômes uniquement par un médecin ?

OUI - NON (passer à 3.3.3)

- Si OUI: Indiquez la date du diagnostic : ____ [date]

  Aujourd'hui, êtes-vous guérie(e) ?
  - o Oui : Indiquez la date de fin de la maladie : ____ [date]
  - o Non

- 3.3.3. Êtes-vous convaincu d'avoir eu la Covid-19 sans avoir été formellement diagnostiqué(e) ?

OUI - NON (Fin du questionnaire)

- Si OUI: Indiquez la date de votre autodiagnostic : ____ [date]

  Aujourd'hui, êtes-vous guérie(e) ?
  - o Oui : Indiquez la date de fin de la maladie : ____ [date]
  - o Non

3.4. Avez-vous été hospitalisé(-e) pour la Covid-19? OUI - NON

- o Si OUI :

  Quelle a été la durée de votre hospitalisation ? ____

  Avez-vous reçu de l'oxygène ? OUI - NON

  Étiez-vous en réanimation ? OUI - NON

  Si OUI : Combien de temps êtes-vous resté(-e) en réanimation ? _____

3.5. Avez-vous suivi un traitement pour la Covid-19? OUI - NON

Si OUI : Lequel ? _____

3.6. Avez-vous été vacciné(-e) contre la Covid-19? OUI - NON

Si OUI :

Avez-vous reçu une seule dose du vaccin? OUI/NON

Avez-vous reçu plus d'une dose de vaccin? OUI/NON

Avec quel vaccin ? _____[liste déroulante] Pfizer, Astrazeneca...je ne sais

3.7. Avez-vous eu les symptômes suivants ? (*Pour cette page, les pages 3.8 à 3.14 s'ouvrent une fois que la personne a répondu à tous les points du 3.7*)

- Symptômes « olfactifs et gustatifs » de la Covid-19 (Pertes de l'Odorat et/ou du Goût): OUI - NON (si OUI, ouvrir 3.8)
- Symptômes « états grippaux » de la Covid-19 (Fièvre, Frissons ou vertiges, Toux, Mal de gorge, Fatigue, Essoufflement, Faiblesse musculaire, Douleurs musculaires (courbatures) ou articulaires, Maux de tête): OUI - NON (si OUI, ouvrir 3.9)
- Symptômes « gastro-intestinaux » de la Covid-19 (Difficultés de déglutition, Difficultés à s'alimenter/boire, Perte d'appétit, Nausées ou vomissements, Diarrhée): OUI - NON (si OUI, ouvrir 3.10)
- Symptômes « cognitifs, neurologiques et psychiatriques » de la Covid-19 (Migraines, Troubles de la mémoire, Troubles de l'attention, Troubles de la parole, Altération de la conscience, État confusionnel, Troubles neurologiques graves, Irritabilité, Troubles anxieux, Dépression, Troubles du sommeil): OUI - NON (si OUI, ouvrir 3.11)
- Symptômes « cutanées et inflammatoires » de la Covid-19 (Éruption cutanée, Démangeaisons, Perte de cheveux, Perte de dents, Conjonctivite (yeux rouges)): OUI - NON (si OUI, ouvrir 3.12)
- Symptômes « cardiaques et rénaux » de la Covid-19 (Troubles rénaux, Troubles cardiaques, Douleurs à la poitrine): OUI - NON (si OUI, ouvrir 3.13)
- Autres symptômes: OUI - NON
  Si OUI:
  - Pouvez-vous le ou les décrire? ____
  - Ouvrir 3.14

3.8. Concernant les symptômes « olfactifs et gustatifs » de la Covid-19 (Pertes de l'odorat et/ou du goût)

Vous avez perdu:

- L'odorat (ex. pertes des sensations olfactives d'aliments, de gaz/fumée, de parfums, de fleurs…) : OUI - NON
- Le goût (ex. pertes des sensations gustatives salées, sucrées, acides, amères) : OUI - NON

<u>Si OUI à Odorat</u> :

- Il s'agissait d'une perte: partielle – totale
- Quand est-ce que ce symptôme est apparu ?: le premier mois **ou** plus tard
- Ce symptôme est-il toujours présent ? OUI – NON
  Si NON, au bout de combien de temps ce symptôme a-t-il disparu: moins d'un mois, Entre 1 et 3 mois, Entre 3 mois et 6 mois, Plus de 6 mois
- Cette perte est ou était également associée à une perception d'odeurs fantômes : OUI – NON
  - Si Oui, décrivez ces odeurs fantômes : ____
- Cette perte est ou était également associée à une perception déformée des odeurs (c'est à dire que les odeurs ne sentent plus comme avant) : OUI – NON
- Qu'elle soit encore présente ou qu'elle ait disparu, cette perte de l'odorat a-t-elle été handicapante dans votre quotidien ? OUI – NON
  Si OUI : Est-ce que cela influence ou a influencé
  - votre bien être psychologique : OUI - NON - Précisez si vous le souhaitez : ___
  - votre alimentation : OUI - NON - Précisez si vous le souhaitez : ___

    - votre vie sociale et relationnelle : OUI - NON - Précisez si vous le souhaitez : ___
    - votre vie professionnelle : OUI - NON - Précisez si vous le souhaitez : ___
        - Quelle est votre profession ? ___
    - autre chose : OUI - NON - Précisez si vous le souhaitez : ___
- Pouvez-vous décrire vos besoins vis-à-vis de ces symptômes et de leurs effets sur votre quotidien ?
    - Je n'ai pas de besoins pour ces symptômes (si coché -> 3.9)
    - Mes besoins sont déjà suffisamment pris en charge (si coché -> 3.9)
    - J'ai besoin d'un suivi
        - médical par un spécialiste : OUI - NON - Précisez si vous le souhaitez : ___
        - psychologique (psychologue, psychiatre) : OUI - NON - Précisez si vous le souhaitez : ___
        - socio-professionnel (assistance sociale, médecine du travail) : OUI - NON - Précisez si vous le souhaitez : ___
        - Autre: ___
- Parmi les possibles thérapies suivantes qui vous permettraient de détecter les odeurs, lesquelles seriez-vous prêt à essayer:
    - Un nouveau traitement médicamenteux sur plusieurs mois : Oui – Non
    - Un entraînement olfactif sur plusieurs mois : Oui – Non
    - Une intervention chirurgicale dans le nez : Oui – Non
    - Une prothèse olfactive nécessitant une chirurgie du cerveau : Oui – Non
    - Une prothèse olfactive que l'on peut installer de manière non invasive : Oui – Non
    - Une autre thérapie : ____
    - Dans quelle situation du quotidien ces possibles thérapies vous seraient-elles le plus utiles? Précisez si vous le souhaitez : ___

<u>Si OUI à Goût</u> :

- Il s'agissait d'une perte: partielle - totale
- Quand est-ce que ce symptôme est apparu ?: le premier mois **ou** plus tard
- Ce symptôme est-il toujours présent ? OUI – NON
  Si NON, au bout de combien de temps ce symptôme a-t-il disparu: moins d'un mois, Entre 1 et 3 mois, Entre 3 mois et 6 mois, Plus de 6 mois
- Cette perte est ou était également associée à une perception de goûts fantômes : OUI – NON
    - Si Oui, décrivez ces goûts fantômes : ____
- Cette perte est ou était également associée à une perception déformée des goûts (c'est à dire que les saveurs n'ont pas le même goût qu'avant) : OUI – NON
- Qu'elle soit encore présente ou qu'elle ait disparu, cette perte du goût a-t-elle été handicapante dans votre quotidien ? OUI – NON
  Si OUI : Est-ce que cela influence ou a influencé
    - votre bien être psychologique : OUI - NON - Précisez si vous le souhaitez : ___
    - votre alimentation : OUI - NON - Précisez si vous le souhaitez : ___
    - votre vie sociale et relationnelle : OUI - NON - Précisez si vous le souhaitez : ___
    - votre vie professionnelle : OUI - NON - Précisez si vous le souhaitez : ___
        - Quelle est votre profession ? ___
    - autre chose : OUI - NON - Précisez si vous le souhaitez : ___
- Pouvez-vous décrire vos besoins vis-à-vis de ces symptômes et de leurs effets sur votre quotidien ?
    - Je n'ai pas de besoins pour ces symptômes (si coché -> 3.9)
    - Mes besoins sont déjà suffisamment pris en charge (si coché -> 3.9)
    - J'ai besoin d'un suivi
        - médical par un spécialiste : OUI - NON - Précisez si vous le souhaitez : ___

    - psychologique (psychologue, psychiatre) : OUI - NON - Précisez si vous le souhaitez : ___
    - socio-professionnel (assistance sociale, médecine du travail) : OUI - NON - Précisez si vous le souhaitez : ___
    - Autre: ___

3.9. Concernant les symptômes « états grippaux » de la Covid-19 (Fièvre, Frissons ou vertiges, Toux, Mal de gorge, Fatigue, Essoufflement, Faiblesse musculaire, Douleurs musculaires (courbatures) ou articulaires, Maux de tête)

- Quand est-ce que le premier de ces symptômes est apparu?: le premier mois **ou** plus tard
- Au moins un de ces symptômes est-il toujours présent ? OUI - NON
  - Si OUI :
    Lequel ou lesquels: ______
  - Si NON, au bout de combien de temps ces symptômes ont-ils disparu (Moins d'un mois, Entre 1 et 3 mois, Entre 3 mois et 6 mois, Plus de 6 mois)
- Qu'ils soient encore présents ou qu'ils aient disparu, ces symptômes ont-ils été handicapants dans votre quotidien ? OUI - NON
  - Si OUI :
    Lequel ou lesquels: ______
  - Si OUI : Est-ce que cela influence ou a influencé
    - votre bien être psychologique : OUI - NON
    - votre alimentation : OUI - NON
    - votre vie sociale et relationnelle : OUI - NON
    - votre vie professionnelle : OUI - NON
    - autre chose : OUI - NON
- Pouvez-vous décrire vos besoins vis-à-vis de ces symptômes et de leurs effets sur votre quotidien ?
  - Je n'ai pas de besoins pour ces symptômes (si coché -> 3.10)
  - Mes besoins sont déjà suffisamment pris en charge (si coché -> 3.10)
  - J'ai besoin d'un suivi
    - médical par un spécialiste : OUI - NON
    - psychologique (psychologue, psychiatre) : OUI - NON
    - socio-professionnel (assistance sociale, médecine du travail) : OUI - NON
    - Autre: ___

3.10. Concernant les symptômes « gastro-intestinaux » de la Covid-19 (Difficultés de déglutition, Difficultés à s'alimenter/boire, Perte d'appétit, Nausées ou vomissements, Diarrhée)
Idem 3.9

3.11. Concernant les symptômes « cognitifs, neurologiques et psychiatriques » de la Covid-19 (Migraines, Troubles de la mémoire, Troubles de l'attention, Troubles de la parole, Altération de la conscience, État confusionnel, Troubles neurologiques graves, Irritabilité, Troubles anxieux, Dépression, Troubles du sommeil)
Idem 3.9

3.12. Concernant les symptômes « cutanées et inflammatoires » de la Covid-19 (Éruption cutanée, Démangeaisons, Perte de cheveux, Perte de dents, Conjonctivite (yeux rouges))
Idem 3.9

3.13. Concernant les symptômes « cardiaques et rénaux » de la Covid-19 (Troubles rénaux, Troubles cardiaques, Douleurs à la poitrine)

Idem 3.9

3.14. Concernant les autres symptômes que vous avez décrits, mais qui n’ont pas été mentionnés dans les questions précédentes
Idem 3.9

Merci de votre participation à ce questionnaire !
Vous pouvez être tenu au courant des résultats globaux sur le site web :
https://project.crnl.fr/covid/

Supplementary Notes 2 (Age, gender, BMI, smoking habits, pregnancy, illness, medication, vaccination, hospitalization)

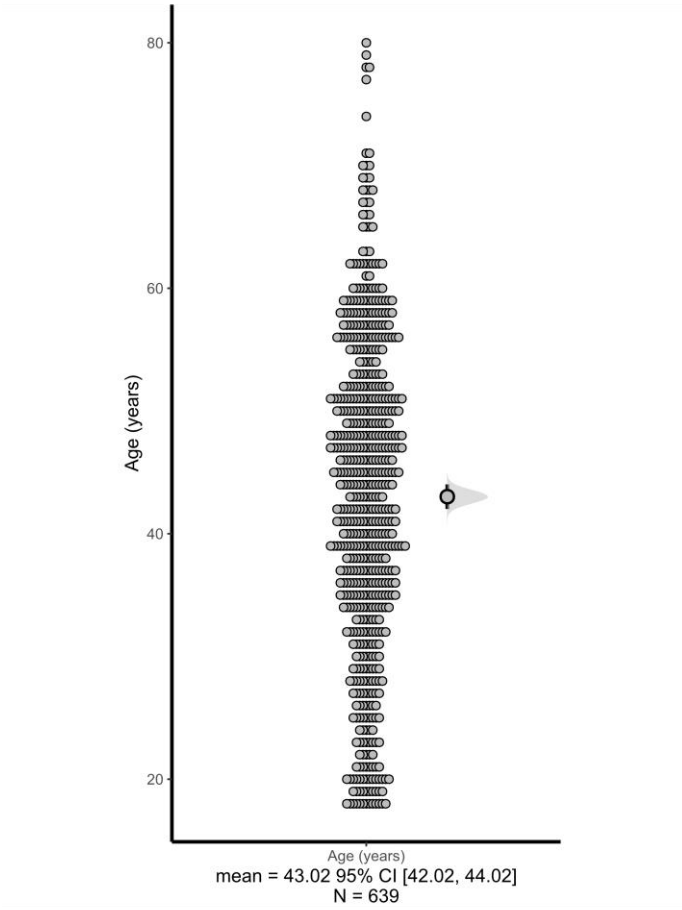

Supplementary Figure 1: Age distribution of the 639 participants retained for analysis. Error bars are 95% confidence intervals in the mean.

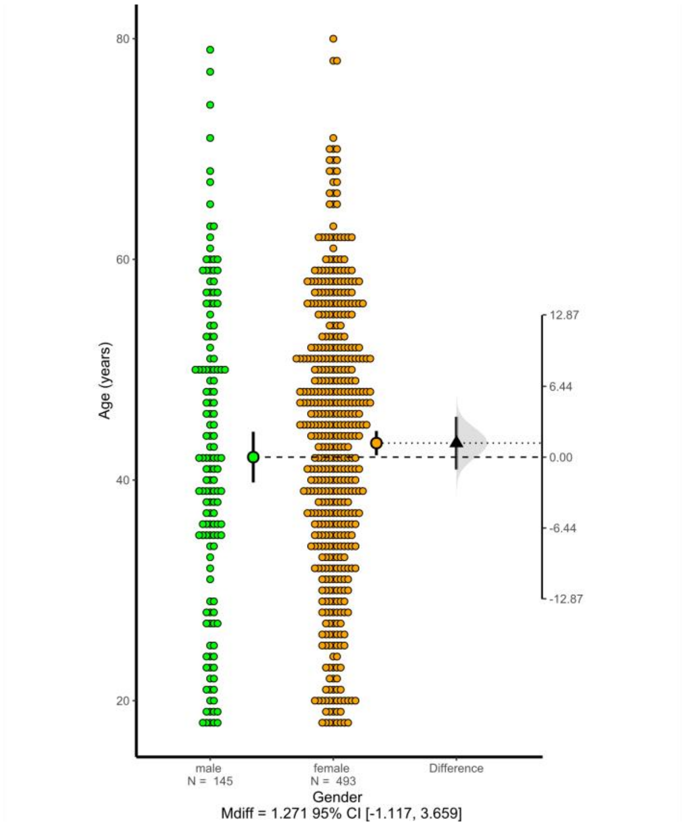

Supplementary Figure 2: Age distribution of the 638 male & female survey participants by gender. Highlighting the mean ages, 95% confidence intervals in the mean, and the difference between the means (p=0.296)

Supplementary Table 1: Age distribution of survey participants divided according to gender

	mean age (years)	standard error in the mean	95% CI		median age (years)	standard deviation	minimum age	maximum age
			lower	upper				
men	42.076	1.171	39.780	44.371	42	14.103	19	79
women	43.347	0.562	42.245	44.449	44	12.487	18	80

Supplementary Table 2: Distribution of body mass indices

	mean BMI (kg/m^2)	standard error in the mean	95% CI		median BMI (kg/m^2)	standard deviation	minimum BMI	maximum BMI
			lower	upper				
men	24.26	0.29	23.69	24.82	23.67	3.46	15.94	34.34
women	23.76	0.22	23.34	24.19	22.72	4.80	16.18	47.83
overall	23.88	0.18	23.52	24.23	23.15	4.53	15.94	47.83

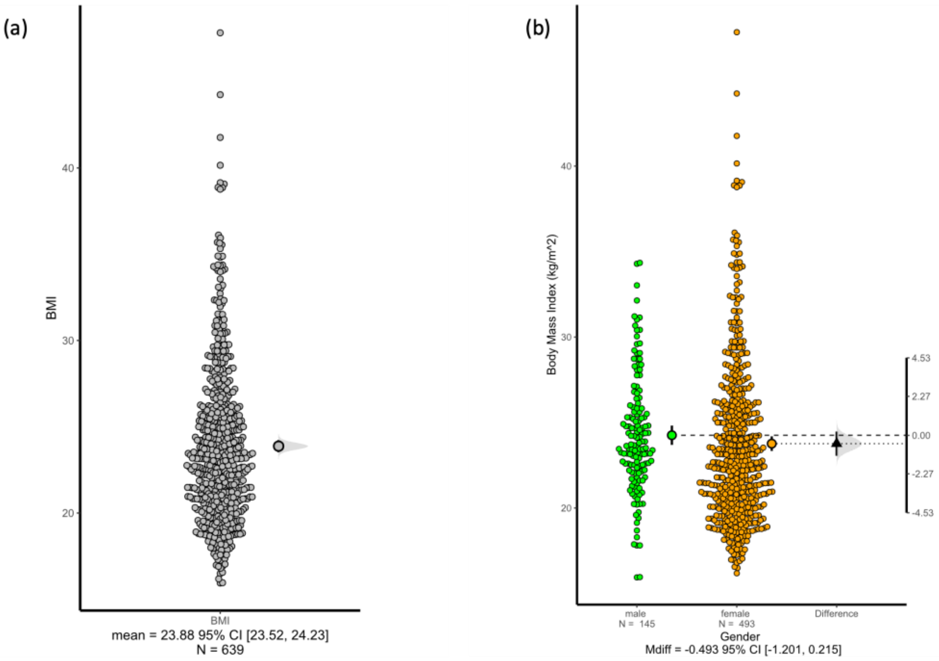

Supplementary Figure 3: Distribution of body mass index for the 639 survey participants (a) all participants (b) participants by gender with the individual with undefined gender excluded. Men (green), women (orange) with means and calculated 95% confidence intervals (p=0.250).

Supplementary Table 3: Correlation between age and BMI

	95% CI		
Pearson's r	Lower	Upper	N
0.15	0.07	0.22	639

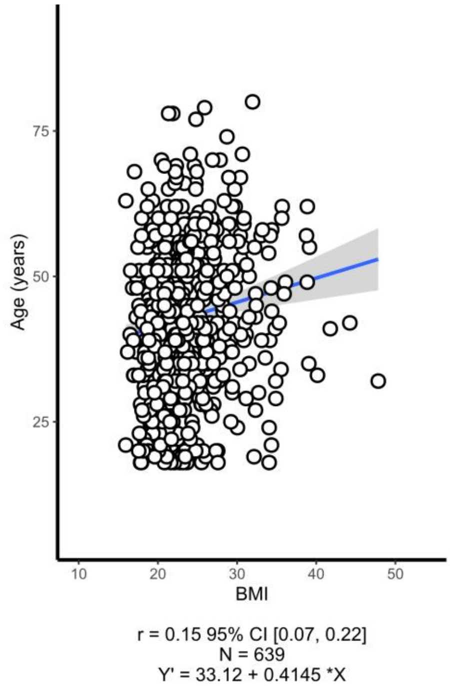

Supplementary Figure 4: Correlation between Age and BMI for the 639 participants. The regression line is indicated in blue with confidence intervals shaded

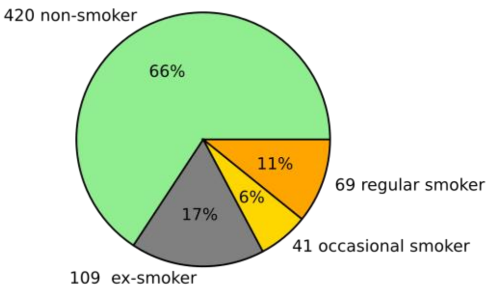

Supplementary Figure 5: Survey respondents' self-declared smoking habits.

Supplementary Table 4: Age distribution of participants' smoking habits

	N	mean age (years)	standard error in the mean	95% CI		median age (years)	standard deviation	minimum age	maximum age
				lower	upper				
non smokers	420	43.03	0.66	41.73	44.32	44.00	13.52	18	80
occasional and regular smokers	110	38.36	1.04	36.33	40.40	39.00	10.88	18	60

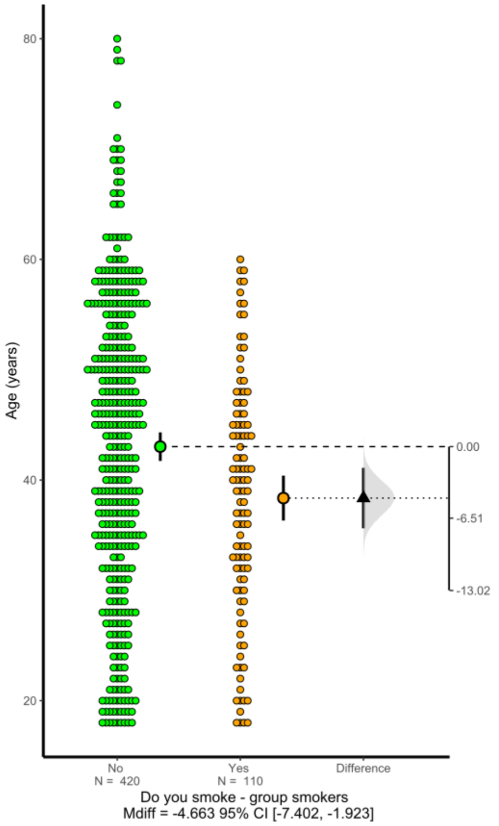

Supplementary Figure 6: Comparison of the age distributions of non-smokers with the group of occasional & regular smokers. Average ages with 95% confidence intervals highlighted. The average age of occasional and regular smokers (38.4 years) was significantly lower than the average age of non-smokers (43.0 years) (p<.001) (for the sake of this comparison the ex-smokers were not included owing to the absence on information related to previous smoking habits and the length of time since the last cigarette.).

Supplementary Table 5: Gender distribution of smokers and non-smokers. Grouping together the occasional and regular smokers and comparing to the non-smokers we find no significant gender difference in the proportion of occasional & regular smokers to non-smokers (men 20.9% and women 20.8%).

	Gender	
	male	female
I used to smoke	30	79
No	91	328
Yes, occasionally	10	31
Yes, every day	14	55

Supplementary Table 6: Pregnancy statistics

Are you pregnant?	Gender	
	male (145)	female (493)
I might be		2
No		482
Yes		9

Supplementary Table 7: Gender distribution of incidence of chronic illness (total number of men and women provided in parentheses)

Do you have a chronic illness?	Gender	
	male (145)	female (493)
I would prefer not to say	2	4
No	118	365
Yes	25	124

Supplementary Table 8: Gender distribution of incidence of regular medication (total number of men and women provided in parentheses)

Are you taking regular medication?	Gender	
	male (145)	female (493)
I would prefer not to say	4	7
No	96	292
Yes	45	194

Supplementary Table 9: : Proportion of vaccinated to non-vaccinated participants by gender. There is no significant difference between these proportions.

					95% CI	
	**# Vaccinated**	**# Not vaccinated**	**Total Number**	**Proportion vaccinated (by gender)**	**Lower**	**Upper**
men	111	34	145	0.77	0.69	0.83
women	360	133	493	0.73	0.69	0.77
difference				0.04	-0.05	0.11

Supplementary Table 10: Proportions of participants vaccinated by diagnosis date. The vaccination campaign began in France on 28th December 2020. 24th January 2021 is one year after the start of the pandemic. Many participants received their vaccination after contracting COVID-19.

					95% CI	
	**# Vaccinated**	**# Not vaccinated**	**Total Number**	**Proportion vaccinated**	**Lower**	**Upper**
all participants	472	167	639	0.74	0.70	0.77
participants diagnosed after 28 December 2020	211	108	319	0.66	0.61	0.71
participants diagnosed after 24 Jan 2021	189	105	294	0.64	0.59	0.70

Supplementary Table 11: Age distributions of vaccinated and non-vaccinated survey participants

	mean age (years)	95% CI		standard error in the mean	median age (years)	standard deviation	minimum age	maximum age
		lower	upper					
not vaccinated	40.41	38.61	42.20	0.92	40	11.83	18	70
vaccinated	43.94	42.76	45.13	0.60	45	13.14	18	80

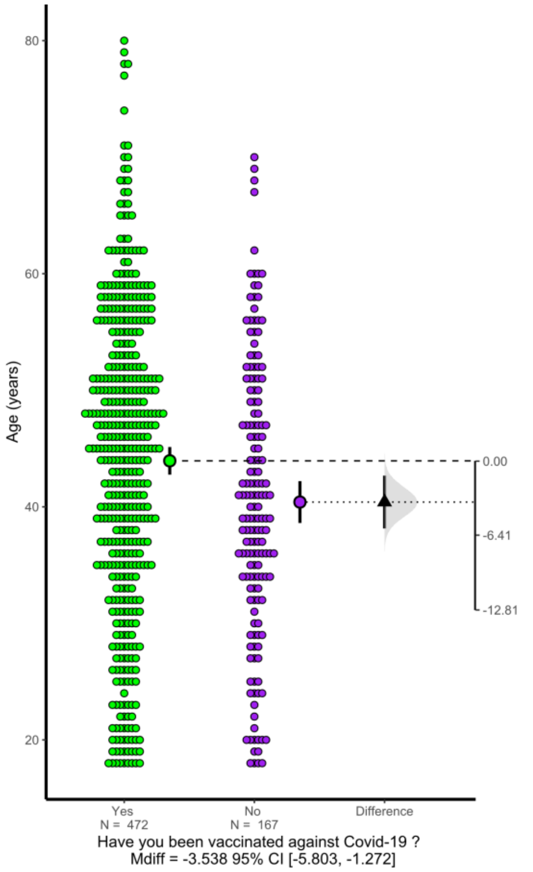

Supplementary Figure 7: Comparison of the age distribution of vaccinated (green) and unvaccinated (purple) participants, showing mean ages with 95% confidence intervals. The average age of vaccinated participants is greater than that of non-vaccinated participants (p=0.002).

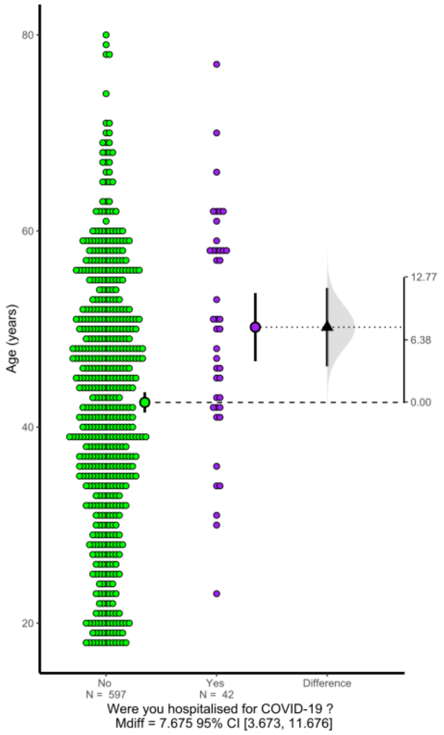

Supplementary Figure 8: Comparison of ages of hospitalized (purple) and not-hospitalized (green) survey participants (p<0.001). Error bars are 95% confidence intervals in the mean.

Supplementary Table 12: Age distribution of participants by hospitalization

		95% CI					
	**Mean age (years)**	**Lower**	**Upper**	**s**	**N**	**t**	**p**
Yes	50.19	46.71	53.67	11.48	42		
No	42.52	41.48	43.55	12.85	597		
difference	7.67	3.67	11.68	12.77	639	3.77	<0.001

Supplementary Table 13: Proportion of participants hospitalized, by gender. There is no significant gender bias in hospitalized patients.

	Were you hospitalized?				95% CI	
	**# Yes**	**# No**	**Total Number**	**Proportion hospitalized (by gender)**	**Lower**	**Upper**
men	7	138	145	0.05	0.02	0.10
women	35	458	493	0.07	0.05	0.10
difference				-0.02	-0.06	0.03

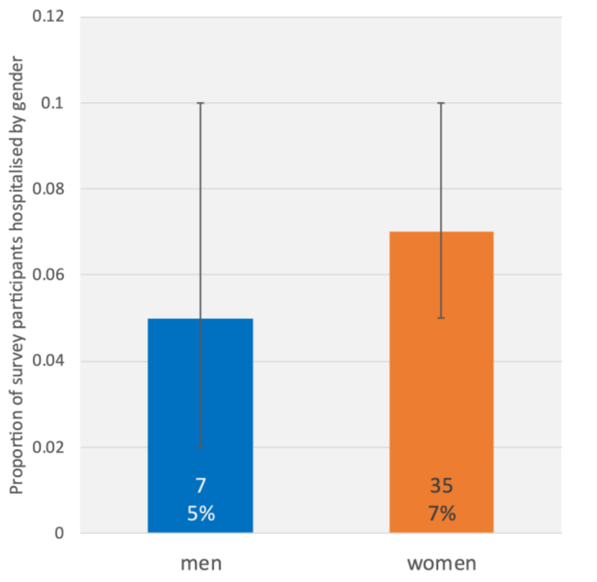

Supplementary Figure 9: Proportion of survey participants hospitalized by gender. Error bars correspond to calculated 95% confidence intervals. Number of participants & percentage by gender indicated inside the bar. There is no significant difference (p=0.332)

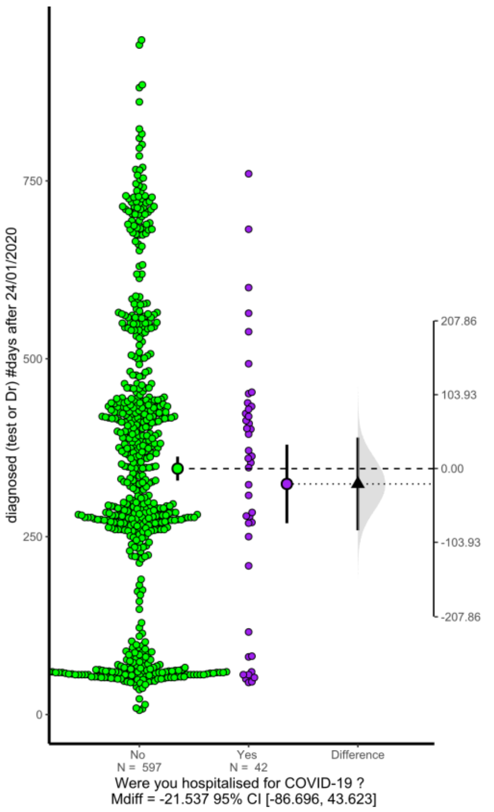

Supplementary Figure 10: Hospitalization as a function of diagnosis date. Error bars are 95% confidence intervals in the mean. Not hospitalized (green); hospitalized (purple) (p=0.517).

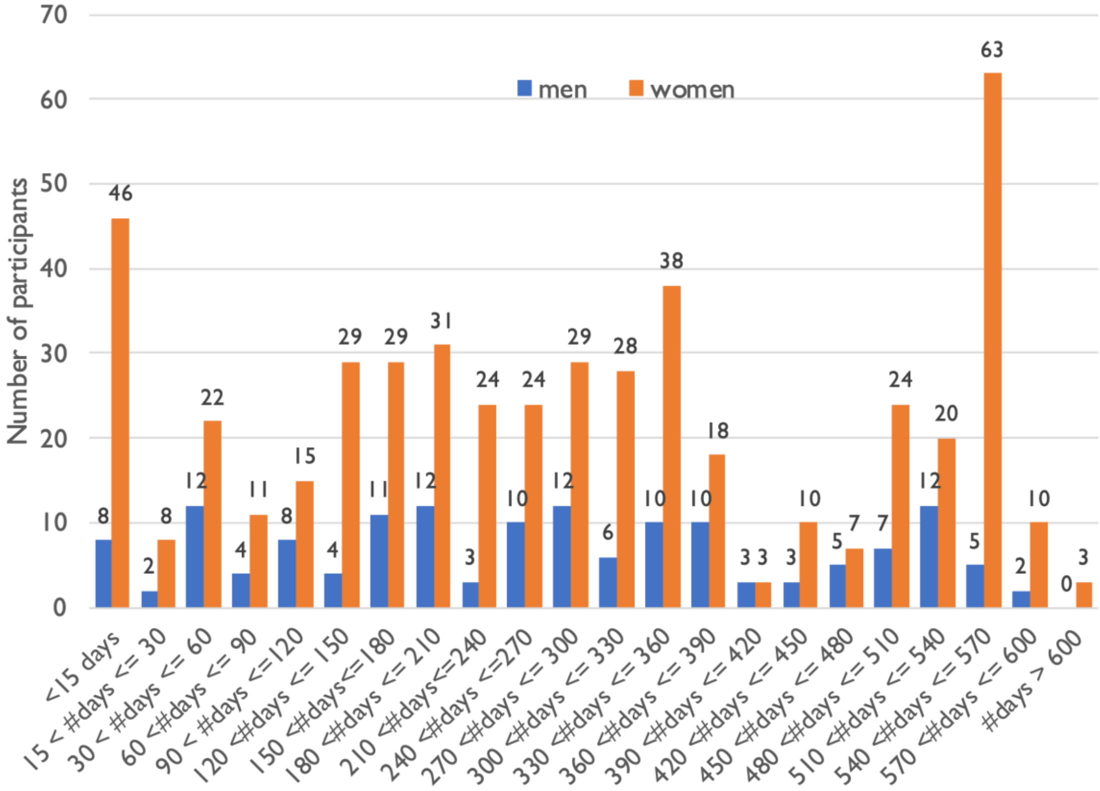

Supplementary Figure 11: Survey participants grouped by the time between their diagnosis and completing the survey (men (blue); women (orange)). The average number of days between the positive COVID-19 diagnosis and completing the survey was 281.4 days. 1 male and 6 female participants completed the survey on the day of their diagnosis.

Supplementary Table 14: Distribution of elapsed time (in days) between a positive diagnosis of COVID-19 and completing the survey for the 639 survey participants. The total number of participants is provided in parentheses. One participant has undefined gender.

	mean number of days since diagnosis	standard error in the mean	95% CI		median	standard deviation	minimum number of days	maximum number of days
			lower	upper				
men (145)	264.44	13.58	237.71	291.17	266	163.48	0	598
women (493)	286.95	8.30	270.61	303.29	284	184.26	0	627
overall (639)	281.42	7.12	267.46	295.38	276	180.04	0	627

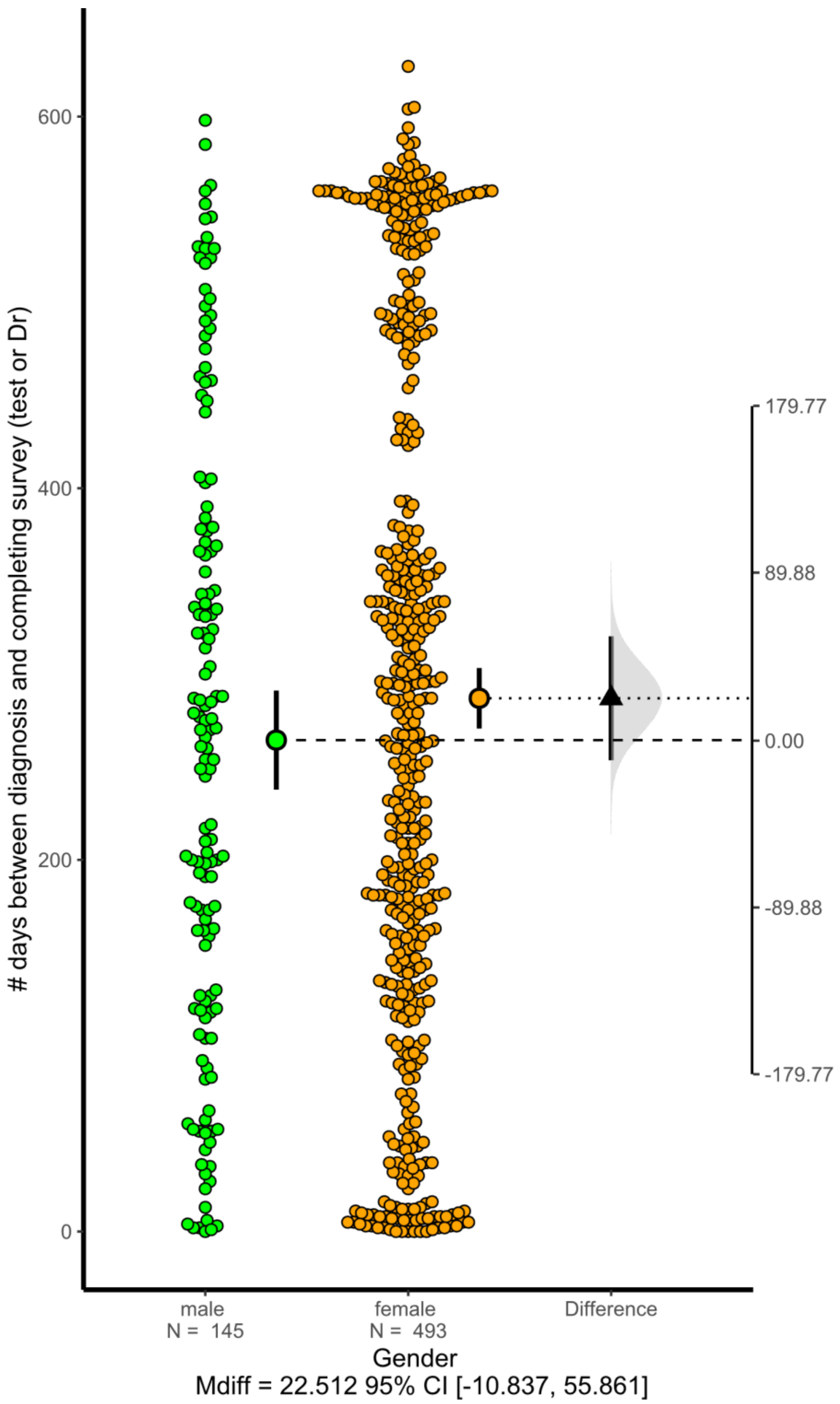

Supplementary Figure 12: Comparison of the average number of days between diagnosis and responding to the survey for men (green) and women (orange). Error bars are calculated 95% confidence intervals. (p=0.185)

Supplementary Notes 3 (geographical information)

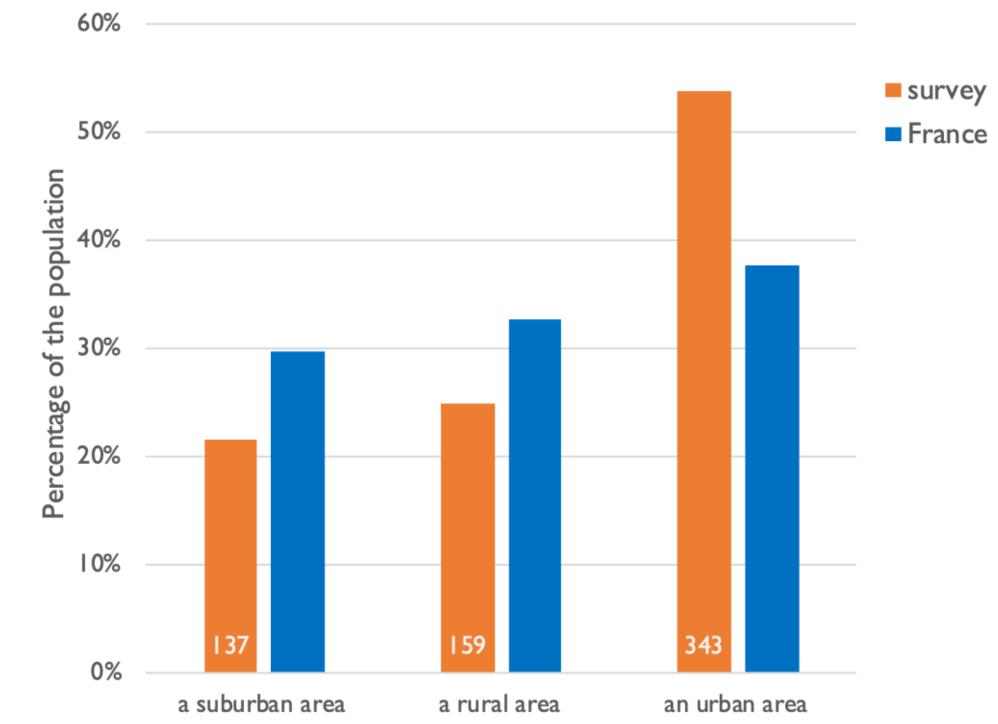

Supplementary Figure 13: Geographical location of survey participants' residence compared to the overall French population (from [1]). Number of survey participants highlighted in white.

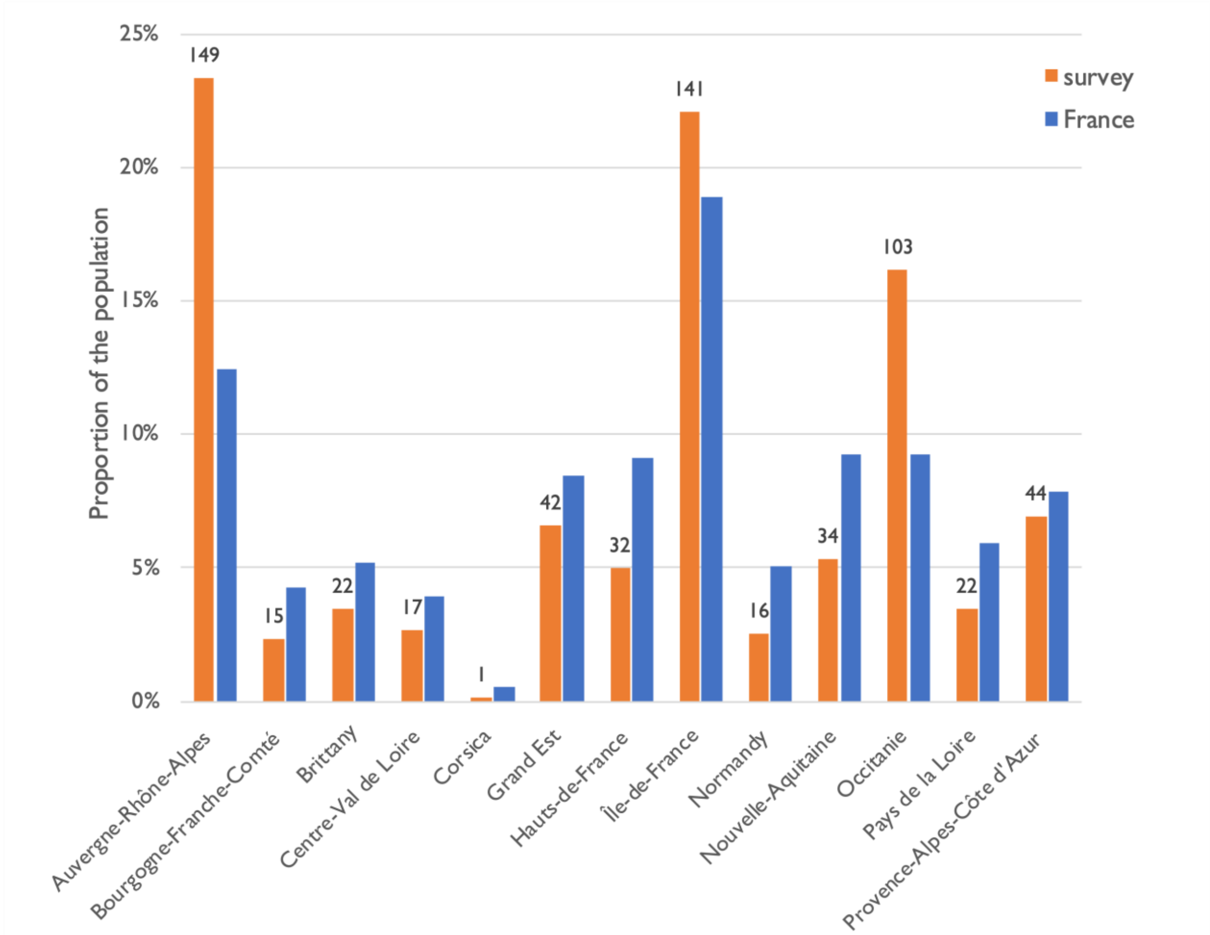

Supplementary Figure 14: Geographical distribution of survey participants (orange) compared to metropolitan France (blue) as a percentage of the population (excluding Guadeloupe (1 participant)). Number of participants indicated. Source of French population data: INSEE population estimates finalised 2021

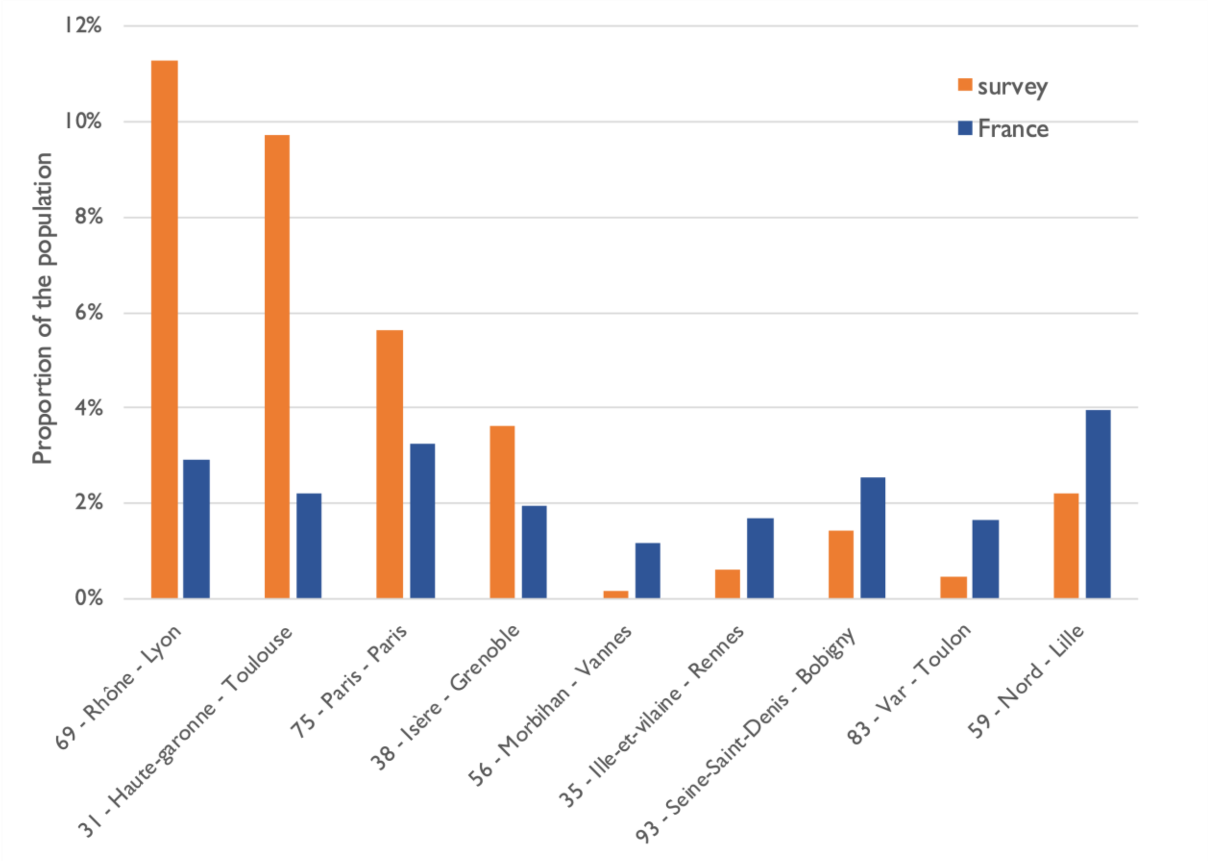

Supplementary Figure 15: French departments with greatest difference between the survey (orange) and metropolitan France (blue) populations (by percentage of total population). The Lyon, Toulouse and Paris areas are over-represented in the survey; the Toulon and Lille areas are under-represented.

Supplementary Notes 4 (symptoms as a function of gender, age; dynamics)

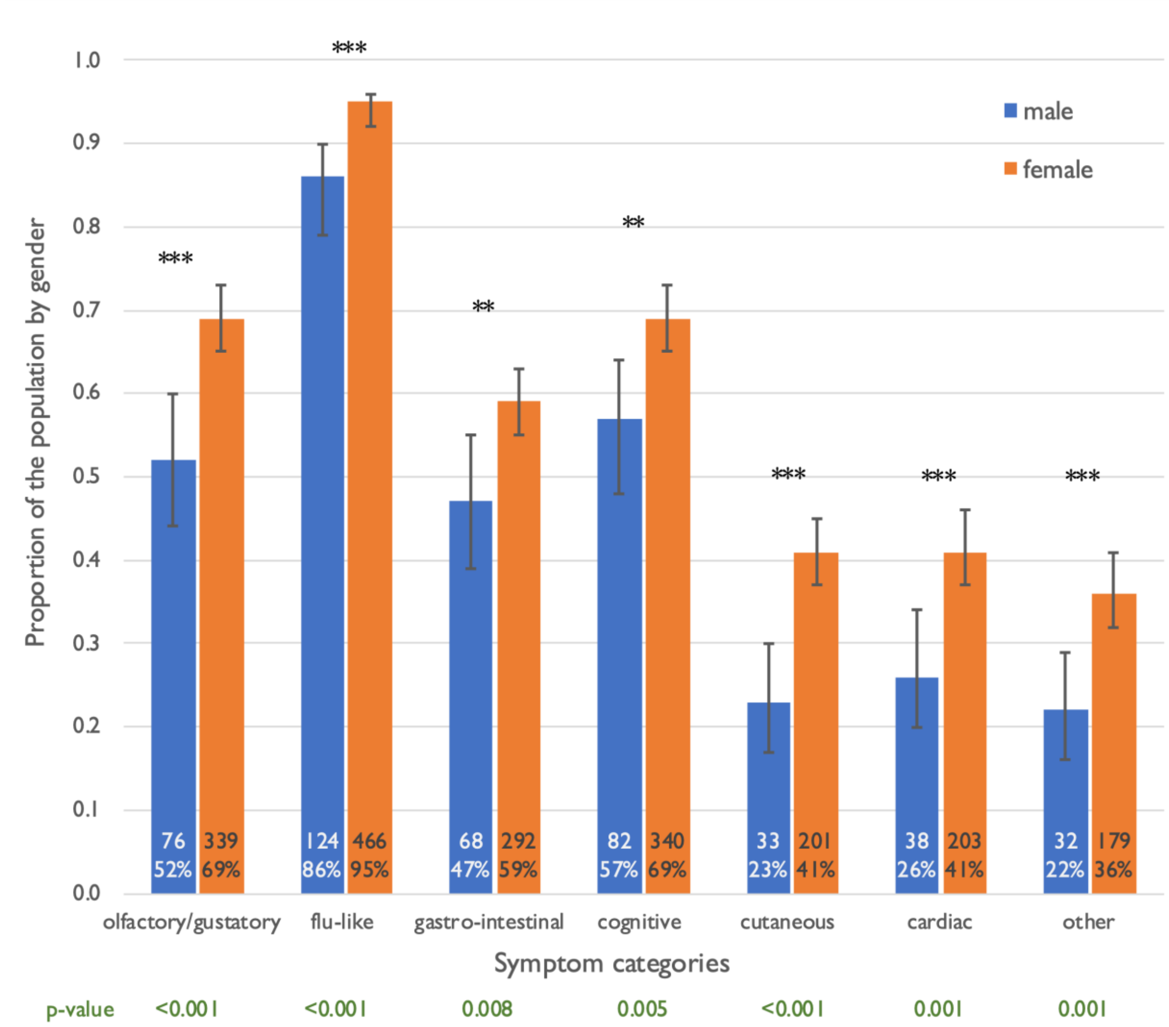

*Supplementary Figure 16: Proportion of survey participants reporting different symptoms by gender (male (blue); female (orange)). Error bars are 95% confidence intervals. Inset in bars: Number of survey participants and percentage of survey population. All gender differences are significant (p<0.01) (significance: *** p≤0.001; ** p≤0.01; * p≤0.05)*

Supplementary Table 15: Proportion of survey participants reporting symptoms by gender, including calculated differences and 95% confidence intervals (638 participants. 1 participant with undefined gender excluded)

		Yes	No	Total N	Proportion (Yes)	95 % CI Lower	95 % CI Upper
Olfactory and Gustatory symptoms	male	76	69	145	0.52	0.44	0.60
	female	339	154	493	0.69	0.65	0.73
	Difference	.	.	.	-0.16	-0.25	-0.07
Lost sense of smell	male	71		145	0.49	0.41	0.57
	female	317		493	0.64	0.60	0.68
	Difference				-0.15	-0.24	-0.06
Lost sense of taste	male	57		145	0.39	0.32	0.47
	female	274		493	0.56	0.51	0.60
	Difference				-0.16	-0.25	-0.07
Flu-like symptoms	male	124	21	145	0.86	0.79	0.90
	female	466	27	493	0.95	0.92	0.96
	Difference	.	.	.	-0.09	-0.16	-0.04
Gastro-intestinal symptoms	male	68	77	145	0.47	0.39	0.55
	female	292	201	493	0.59	0.55	0.63
	Difference	.	.	.	-0.12	-0.21	-0.03
Cognitive, Neurological & Psychiatric symptoms	male	82	63	145	0.57	0.48	0.64
	female	340	153	493	0.69	0.65	0.73
	Difference	.	.	.	-0.12	-0.21	-0.04
Cutaneous & Inflammatory symptoms	male	33	112	145	0.23	0.17	0.3
	female	201	292	493	0.41	0.37	0.45
	Difference	.	.	.	-0.18	-0.26	-0.09
cardiac or renal symptoms	male	38	107	145	0.26	0.2	0.34
	female	203	290	493	0.41	0.37	0.46
	Difference	.	.	.	-0.15	-0.23	-0.06
at least one of the symptoms	male	137	8	145	0.94	0.89	0.97
	female	487	6	493	0.99	0.97	0.99
	Difference	.	.	.	-0.04	-0.09	-0.01

Supplementary Table 16 Statistical significance of gender differences in symptoms reported $P(symptom)_{male}$ vs $P(symptom)_{female}$

Symptom category	χ2	z test	p-value
Olfactory and Gustatory symptoms	13.17	3.63	<0.001
Flu-like symptoms	13.06	3.61	<0.001
Gastro-intestinal symptoms	6.93	2.63	0.008
Cognitive, Neurological & Psychiatric symptoms	7.71	2.78	0.005
Cutaneous & Inflammatory symptoms	15.65	3.96	<0.001
cardiac or renal symptoms	10.68	3.27	0.001
at least one of the symptoms	9.65	3.11	0.002

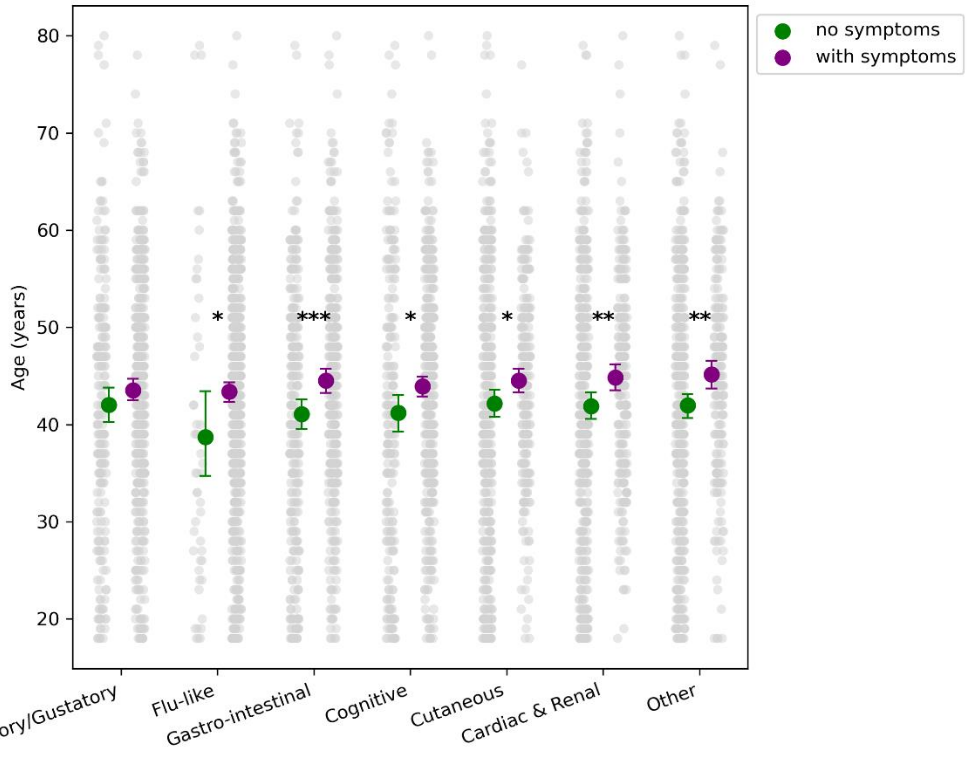

*Supplementary Figure 17: Symptoms experienced as a function of age. Points are averages over the ages of people reporting symptoms in that category. The average age of those reporting symptoms is greater than the average age of those not reporting symptoms, except for olfactory/gustatory symptoms where there is no statistically significant difference. Error bars are calculated 95% CI. Significance: *** p<0.001; ** p<0.01; * p<0.03. Purple: with symptoms; Green: no symptoms; grey dots: raw data*

Supplementary Table 17: Age distributions of participants as a function of symptoms experienced

		N	mean age	standard error in the mean	95% CI		median	standard deviation	min age	max age
					lower	upper				
Olfactory & Gustatory loss	Yes	416	43.54	0.61	42.34	44.74	44	12.46	18	78
	No	223	42.05	0.91	40.25	43.84	42	13.65	18	80
	Difference	639	1.49		-0.61	3.59		12.89		
Flu-like symptoms	Yes	590	43.38	0.52	42.36	44.39	44	12.52	18	80
	No	49	38.73	2.34	34.15	43.32	35	16.35	18	79
	Difference	639	4.64		0.89	8.39		12.85		
Gastro-intestinal symptoms	Yes	360	44.52	0.64	43.26	45.77	45	12.16	18	80
	No	279	41.09	0.81	39.49	42.69	41	13.57	18	79
	Difference	639	3.43		1.42	5.43		12.79		
Cognitive, neurological & psychiatric symptoms	Yes	423	43.95	0.56	42.86	45.04	45	11.43	18	80
	No	216	41.20	1.04	39.17	43.24	40	15.23	18	79
	Difference	639	2.74		0.64	4.85		12.84		
Cutaneous & inflammatory symptoms	Yes	234	44.54	0.68	43.20	45.87	45	10.40	18	77
	No	405	42.14	0.70	40.77	43.52	42	14.08	18	80
	Difference	639	2.40		0.32	4.47		12.85		
Cardiac or Renal symptoms	Yes	241	44.86	0.69	43.51	46.22	45	10.69	18	80
	No	398	41.90	0.70	40.53	43.28	41	13.96	18	79
	Difference	639	2.96		0.90	5.01		12.83		
Other symptoms	Yes	211	45.14	0.74	43.69	46.60	46	10.77	18	79
	No	428	41.97	0.66	40.67	43.28	42	13.72	18	80
	Difference	639	3.17		1.05	5.29		12.82		
Any of the symptoms	Yes	625	43.17	0.51	42.18	44.17	44	12.66	18	80
	No	14	36.21	5.49	25.44	46.99	27.5	20.53	18	79
	Difference	639	6.96		0.13	13.79		12.87		

Supplementary Table 18: Age distribution of participants by symptoms reported

**Symptom category**	**Average age no symptoms (years)**	**95% CI**	**Average age with symptoms (years**	**95% CI**	**t**	**p**	**N with symptom**	**% of survey population with these symptoms**
Olfactory/ gustatory	42.0	[40.3,43.8]	43.5	[42.3,44.7]	1.39	0.164	416	65%
Flu-like	38.7	[34.2,43.3]	43.4	[42.4,44.4]	2.43	0.015	590	92%
Gastro-intestinal	41.1	[39.5,42.7]	44.5	[43.3,45.8]	3.36	<0.001	360	56%
Cognitive	41.2	[39.2,43.2]	44.0	[42.9,45.0]	2.56	0.011	423	66%
Cutaneous	42.1	[40.8,43.5]	44.5	[43.2,45.9]	2.27	0.024	234	37%
Cardiac & renal	41.9	[40.5,43.3]	44.9	[43.5,46.2]	2.83	0.005	241	38%
Other	42.0	[40.7,43.3]	45.1	[43.7,46.6]	2.94	0.003	211	33%

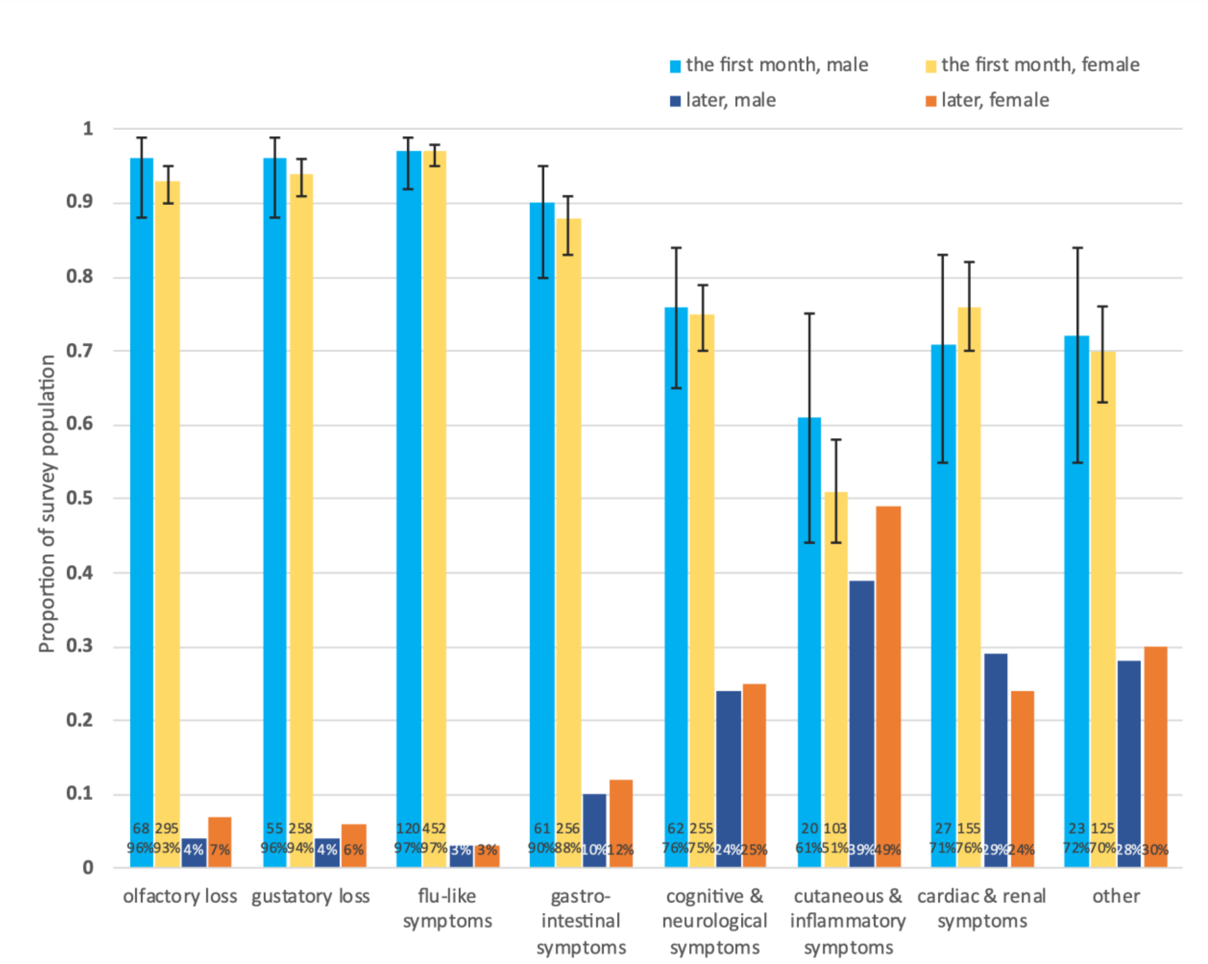

Supplementary Figure 18: Gender comparison for symptom onset. Error bars represent calculated 95% confidence intervals. Number of participants, and percentage of participants by gender indicated in bars. There are no significant gender differences (p values between 0.318 and 0.909)

*Supplementary Table 19: Statistical significance of gender differences in delay in **onset** of symptoms reported $P(symptom)_{male}$ vs $P(symptom)_{female}$*

symptom category	χ2	p value for gender difference
olfactory loss	0.71	0.400
gustatory loss	0.50	0.480
flu-like symptoms	0.02	0.899
gastro-intestinal symptoms	0.22	0.641
cognitive & neurological symptoms	0.01	0.909
cutaneous & inflammatory symptoms	1.00	0.318

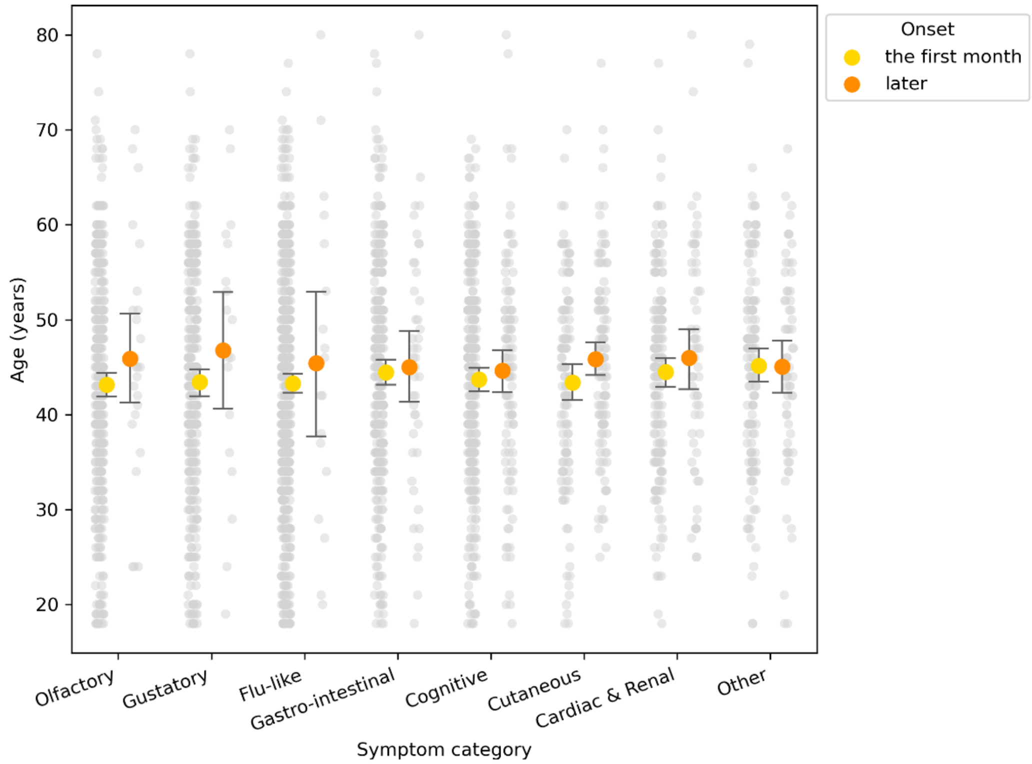

Supplementary Figure 19: Average age of symptom onset (by symptom category). Error bars are calculated 95% CI. There are no significant differences. (p-values: 0.294, 0.279, 0.477, 0.771, 0.470, 0.069, 0.356, 0.956 respectively)

Supplementary Table 20: Statistical significance by age for those with and without delayed onset symptoms

	t	df	p value (with vs without delayed onset symptoms)
olfactory loss	1.05	387	0.294
gustatory loss	1.08	330	0.279
flu-like symptoms	0.71	588	0.477
gastro-intestinal symptoms	0.29	358	0.771
cognitive & neurological symptoms	0.72	421	0.470
cutaneous & inflammatory symptoms	1.83	232	0.069
cardiac & renal symptoms	0.93	239	0.356
other	0.06	209	0.956

Supplementary Table 21: The distribution of #days symptomatic (for any symptom) for the 200 people reporting recovery

	Mean (days)	Median (days)	s	min (days)	max (days)	25th percentile	75th percentile	N
# days symptomatic (if recovered)	54.4	18	92.3	0	462	8	39	200

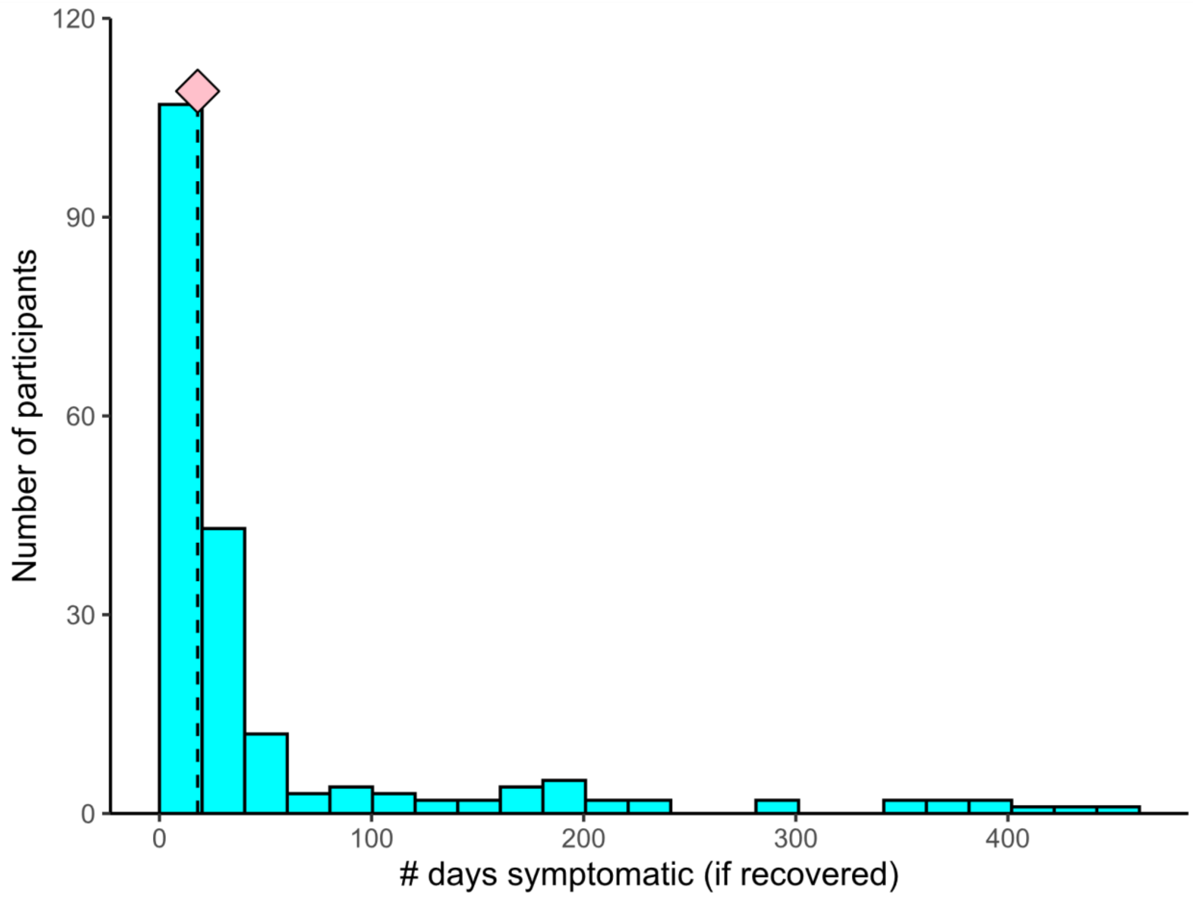

Supplementary Figure 20: The distribution of the 200 participants reporting recovery by the number of days they were symptomatic. Only 200/639 participants (31%) reported recovery. The median number of days between diagnosis and recovery (for the 200 people reporting recovery) was 18. Only 53 people in our sample recovered after more than 35 days, with 33 between three and fifteen months after diagnosis.

*Supplementary Table 22: Proportion of survey participants reporting recovery from COVID-19 by gender including calculated difference and 95% confidence intervals. * These data include the single individual with undefined gender*

		Yes	No	Total N	Proportion recovered (by gender)	95 % CI Lower	95 % CI Upper
Reported recovery from COVID-19	male	66	79	145	0.42	0.38	0.54
	female	133	360	493	0.27	0.23	0.31
	Difference				0.19	0.10	0.27
	overall	200	439	639	0.31	0.28	0.35

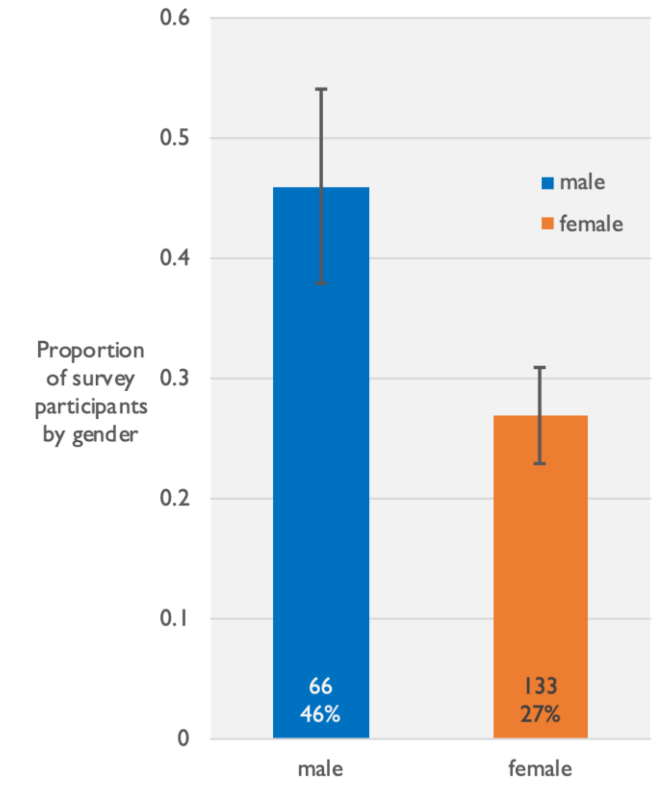

Supplementary Figure 21: Proportion of survey participants reporting recovery from COVID by gender (blue (male); orange (female)). Error bars are calculated 95% CI. More men than women report recovery. The number of participants and percentage of survey population by gender is provided inside the bars. Significance: χ^2=17.94 p<0.0001

Supplementary Table 23: Reported reovery from COVID-19 by age with statistical significance

		95% CI					
Report recovery	Mean age (years)	Lower	Upper	s	N	t	p
Yes	40.06	38.02	42.10	14.66	200		
No	44.37	43.27	45.47	11.78	439		
Difference	-4.31	-6.45	-2.17	12.75	639	**-3.96**	**<0.001**

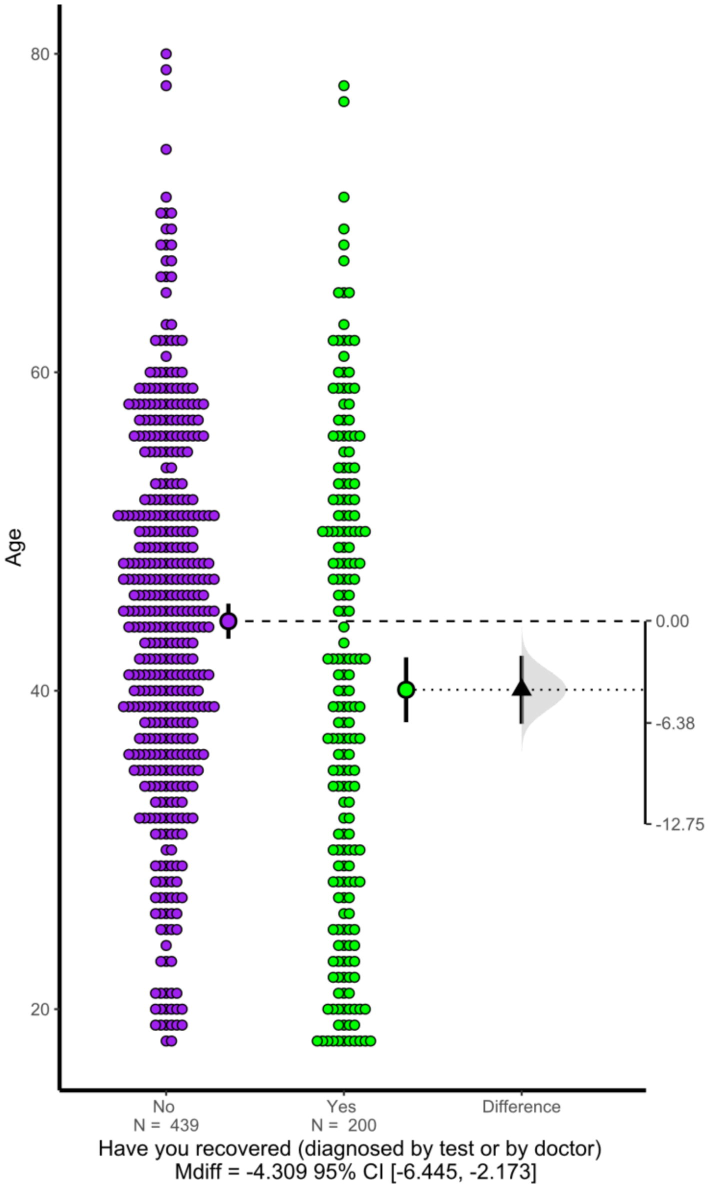

Supplementary Figure 22: Comparison of the age distributions of participants reporting recovery (green) with participants not reporting recovery (purple). The average age of participants reporting recovery is lower than the average age of participants not reporting recovery (p<0.001). Error bars are 95% confidence intervals in the mean.

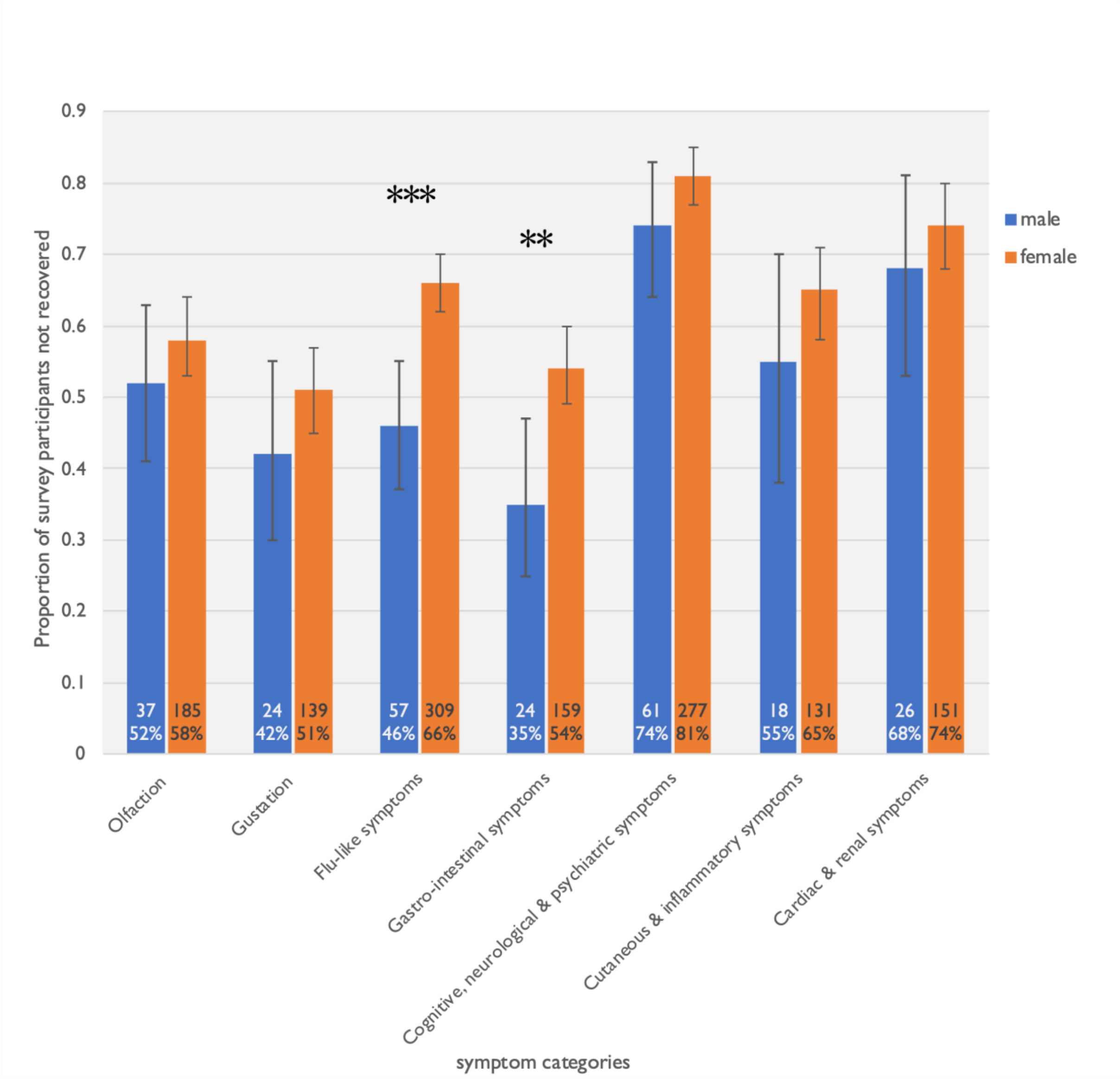

*Supplementary Figure 23: The proportion of survey participants by symptom category reporting that they still had COVID symptoms at the time of completing the survey (blue (male), orange (female)). Error bars are calculated 95% CI. When asking participants whether they still experience symptoms of various kinds, more women than men report symptoms but the difference is only statistically significant for flu-like and gastro-intestinal symptoms. Inset in the bars: The number of participants and percentage by symptom category and gender. Significance: *** p<0.001; ** p=0.004.*

Supplementary Table 24: Statistical significance of gender differences in reporting continued symptoms $P(symptom)_{male}$ vs $P(symptom)_{female}$

Symptom category	χ2	z test	p-value
loss of olfaction	0.925	0.96	0.336
loss of gustation	1.404	1.19	0.236
Flu-like symptoms	17.206	4.15	<0.001
Gastro-intestinal symptoms	8.100	2.85	0.004
Cognitive, Neurological & Psychiatric symptoms	2.077	1.44	0.149
Cutaneous & Inflammatory symptoms	1.384	1.18	0.239
cardiac or renal symptoms	0.584	0.76	0.445
other	0.925		0.336

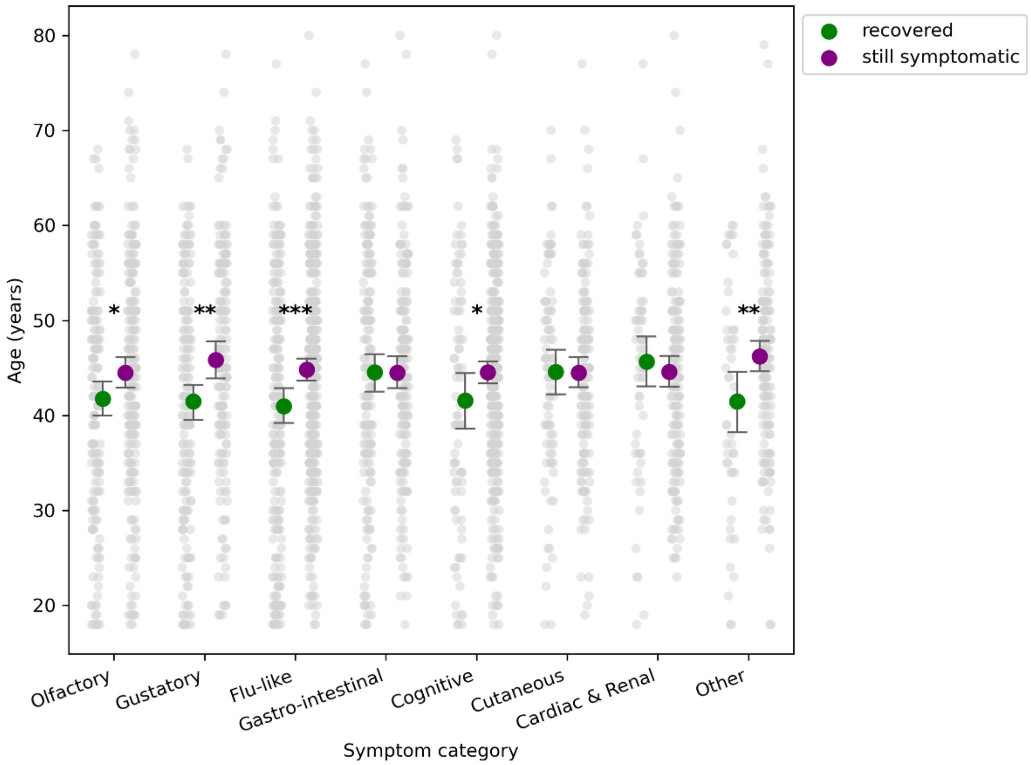

*Supplementary Figure 24: Comparison of the average age of those still reporting symptoms (purple) with those reporting recovery (green) by symptom category. Those participants stating that they still experience symptoms are older on average than those not reporting symptoms, except for gastro-intestinal, cutaneous & inflammatory and cardiac & renal symptoms where no statistically significant difference was found. Significance: * p≤0.05 ** p≤0.01 *** p<0.001*

Supplementary Table 25: Statistical significance of the average difference in age of those reporting continued symptoms versus those not reporting continued symptoms by symptom category

	t	df	p-value
olfactory loss	2.13	387	0.034
gustatory loss	3.18	330	0.002
flu-like symptoms	3.66	588	<0.001
gastro-intestinal symptoms	-0.01	358	0.989
cognitive & neurological symptoms	2.15	421	0.032

cutaneous & inflammatory symptoms	60.06	232	0.956
cardiac & renal symptoms	-0.69	239	0.490
other	2.71	209	0.007

Supplementary Notes 5 (perceptions)

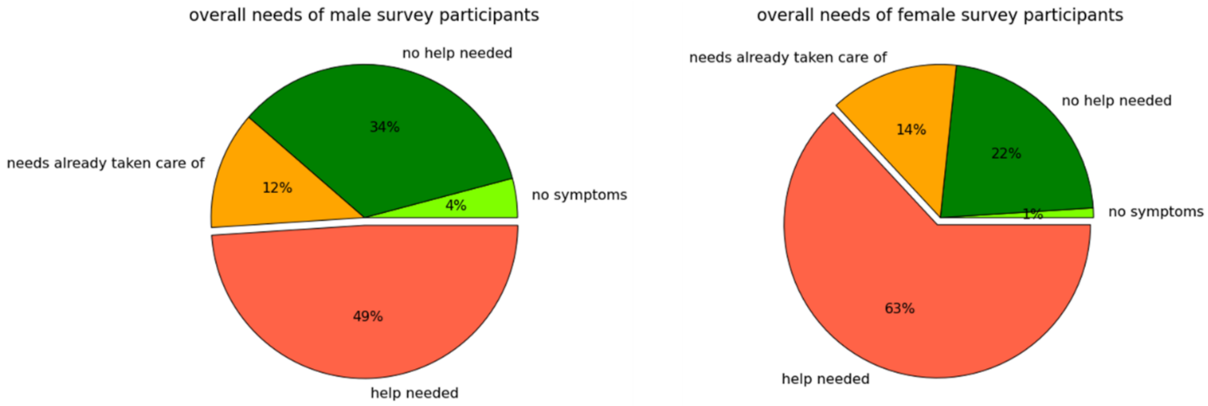

Supplementary Figure 25: Comparison of male (left) and female (right) participants' perception of the help they need with their symptoms. More women than men need help with their symptoms. Pale green: no symptoms; dark green: no help needed; orange: sufficient help has already been supplied; Red: help needed. Associated numbers and statistics provided in Supplementary Table 26

Supplementary Table 26: Statistical significance of gender differences for the perception of help needed as a consequence of COVID-19 symptoms

	χ^2	p value for gender difference	N (men)	N (women)
No symptoms	6.452	0.011 (very small numbers)	6	5
No help needed	8.833	0.003	50	110
Needs met	0.134	0.714	18	67
Help needed	9.295	0.002	71	311

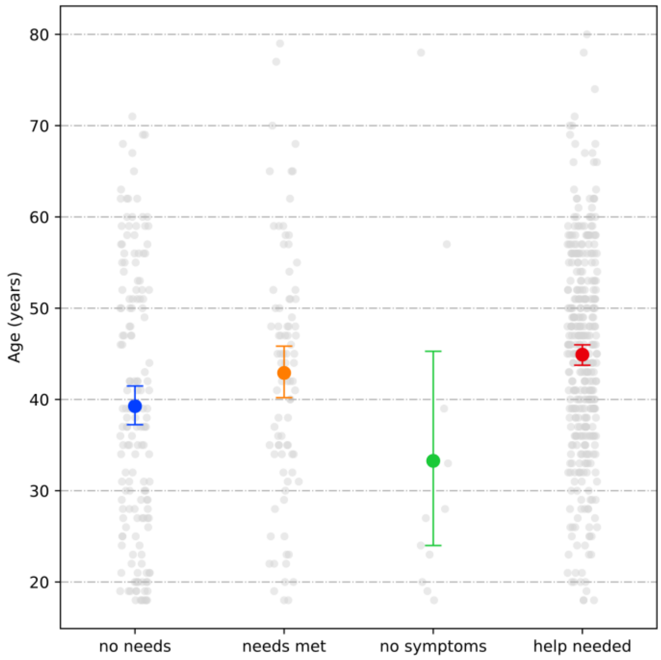

Supplementary Figure 26: Comparison of average ages of participants by perception of need with respect to their symptoms. Error bars are 95% confidence intervals in the mean. N(no symptoms): 11; N(no needs): 161; N(needs met): 85; N(help needed): 382. People with no needs are younger on average than people needing help with their symptoms (see Supplementary Table 27).

Supplementary Table 27: Statistical comparisons of the differences in average age between participants by perceived needs

Group 1	Group 2	t	df	p-value
no symptoms	no needs	1.30	170	0.197
needs met	help needed	1.42	465	0.155
no needs	needs met	1.90	244	0.059
no needs	help needed	4.87	541	<0.001

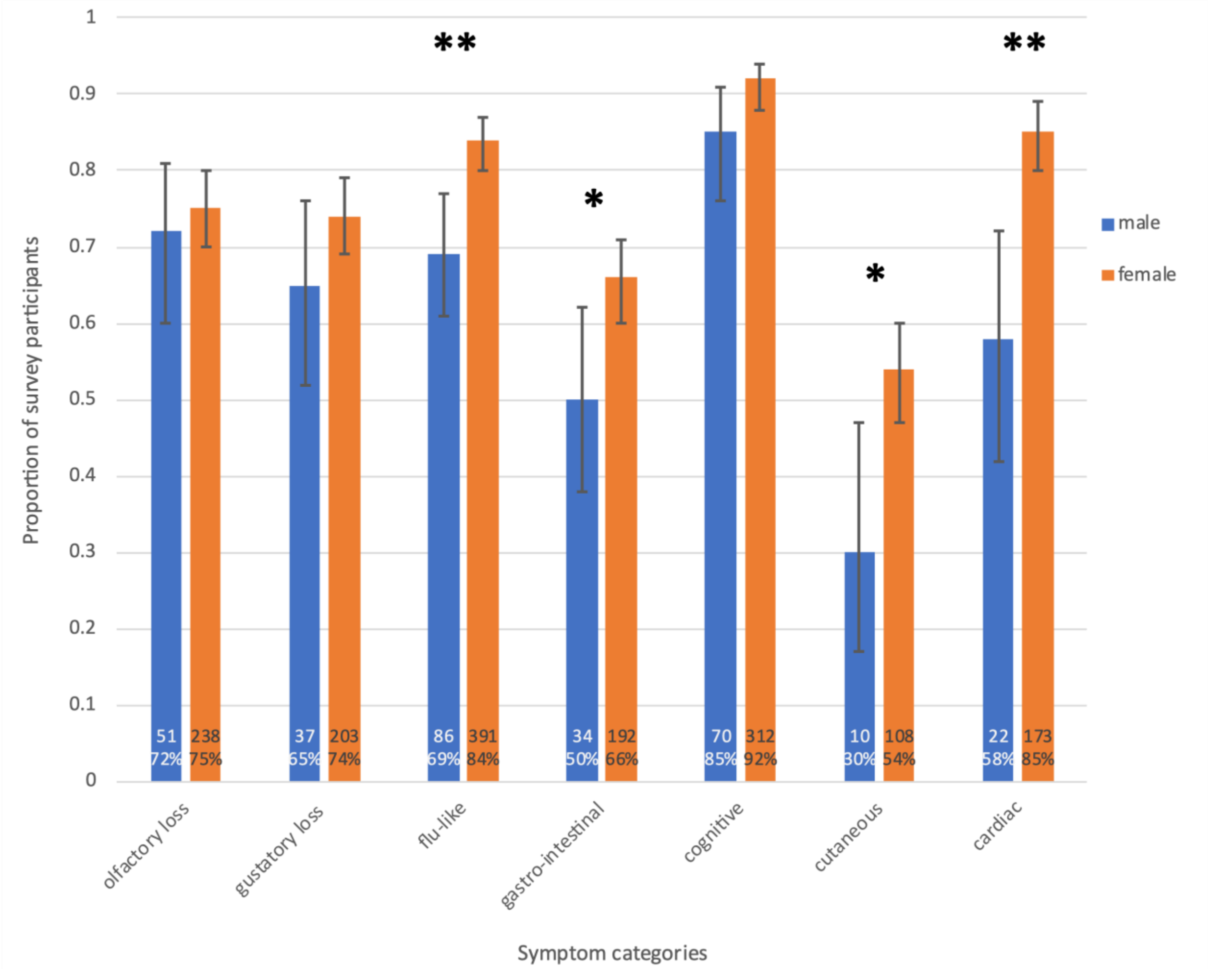

*Supplementary Figure 27: Participants who reported that their symptoms were a handicap in their everyday life, as a proportion of those suffering those symptoms, by gender. More women than men find their flu-like, gastro-intestinal, cutaneous & inflammatory and cardiac & renal symptoms handicap them in their everyday life. Error bars are calculated 95% confidence intervals. The number of participants and the proportion by gender of those reporting each symptom is provided inside the bars. Significance of differences: ** p<0.001; * 0.01<p<0.02*

Supplementary Table 28: Statistical significance of gender differences in reporting symptoms were handicapping P(symptom)$_{male}$ vs P(symptom)$_{female}$

Symptom category	χ2	z test	p-value
loss of olfaction	0.32	0.57	0.570
loss of gustation	1.99	1.41	0.158
Flu-like symptoms	13.39	3.66	<0.001
Gastro-intestinal symptoms	5.86	2.42	0.016
Cognitive, Neurological & Psychiatric symptoms	3.15	1.78	0.076
Cutaneous & Inflammatory symptoms	6.22	2.49	0.013
cardiac or renal symptoms	15.48	3.93	<0.001

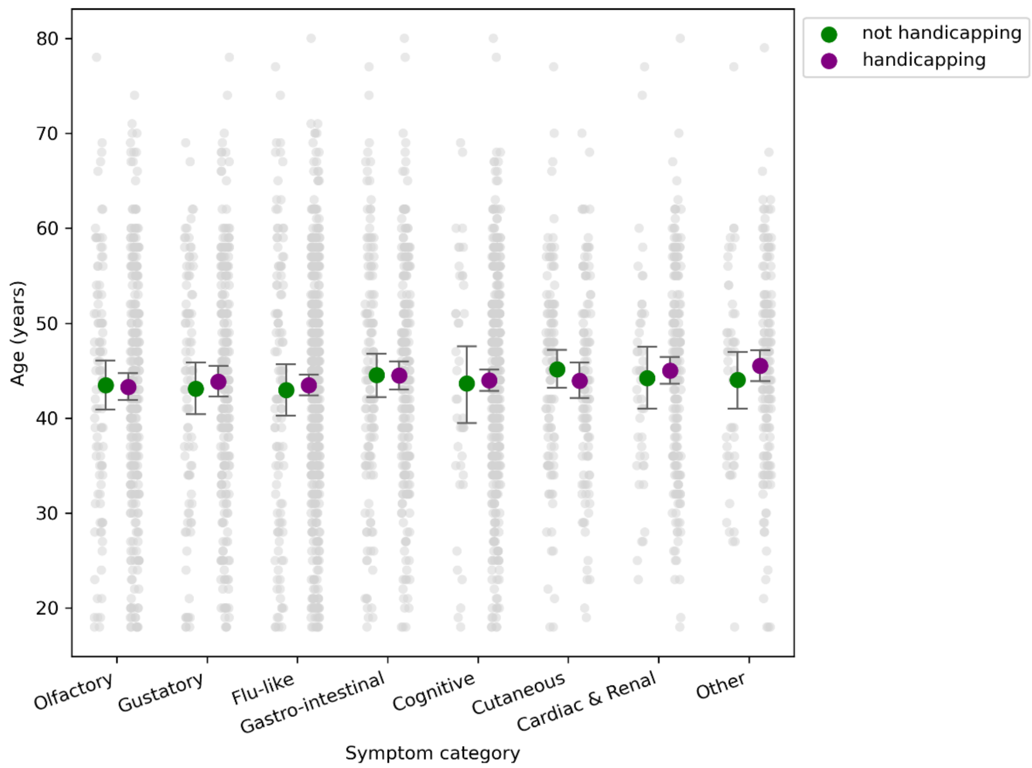

Supplementary Figure 28: Comparison of the average age of those finding their symptom a handicap with those who don't, by symptom category. There are no statistically significant differences in age between those that find their symptoms handicap them in their everyday life, and those that don't. Error bars are 95% confidence intervals in the mean.

Supplementary Table 29: Statistical significance of the difference in average age of those participants finding their symptoms handicap them in their everyday life and the average age of those that don't.

	t	df	p value for age difference
olfactory loss	-0.12	387	0.906
gustatory loss	0.48	330	0.632
flu-like symptoms	0.38	588	0.704
gastro-intestinal symptoms	-0.02	358	0.98
cognitive & neurological symptoms	0.17	421	0.865
cutaneous & inflammatory symptoms	-0.87	232	0.383
cardiac & renal symptoms	0.44	239	0.661
other	0.85	209	0.396

(a)

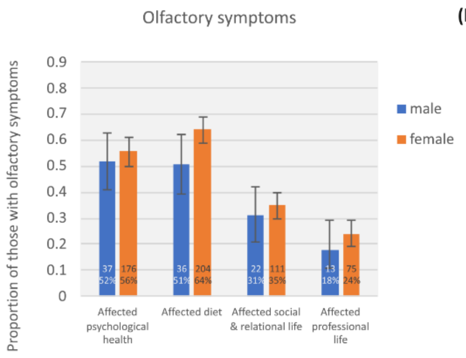

	χ^2	p value for gender difference
Psychological health	0.272	0.602
diet	4.580	0.032
Social & relational life	0.418	0.518
Professional life	0.947	0.331

(b)

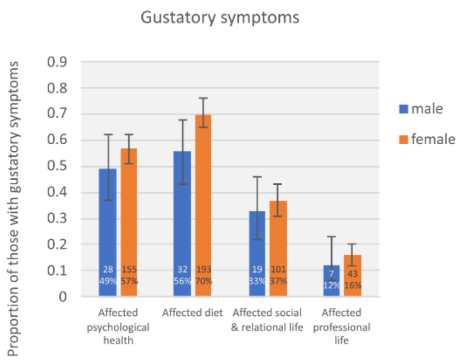

	χ^2	p value for gender difference
Psychological health	1.058	0.304
diet	4.431	0.035
Social & relational life	0.254	0.614
Professional life	0.429	0.513

(c)

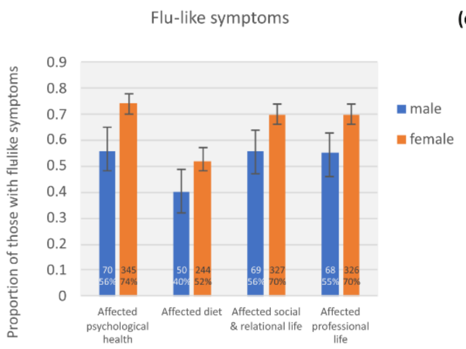

	χ^2	p value for gender difference
Psychological health	14.513	0.000
diet	5.677	0.017
Social & relational life	9.365	0.002
Professional life	10.091	0.001

(d)

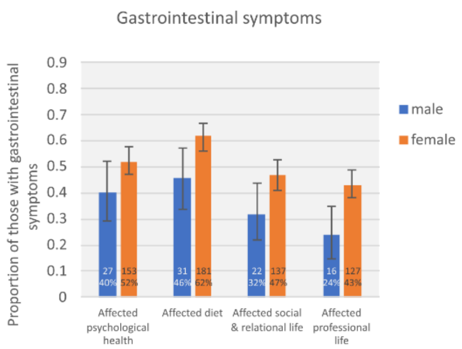

	χ^2	p value for gender difference
Psychological health	3.554	0.059
diet	6.126	0.013
Social & relational life	4.745	0.029
Professional life	9.181	0.002

*Supplementary Figure 29: Impact of symptoms on everyday life (psychological health, diet, social & professional life) by gender. (a) olfactory symptoms (b) gustatory symptoms (c) flu-like symptoms (d) gastro-intestinal symptoms. * Note the small number of men. The statistical calculations are unreliable. Error bars are 95% confidence intervals.*

(e)

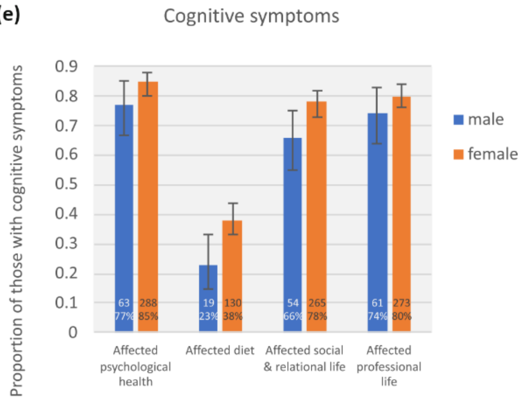

	χ²	p value for gender difference
Psychological health	2.929	0.087
diet	6.564	0.010
Social & relational life	5.232	0.022
Professional life	1.395	0.238

(f)

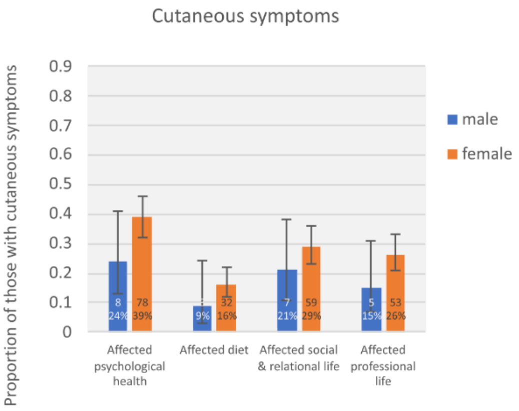

	χ²	p value for gender difference
Psychological health	2.586	0.108 (numbers small)
diet	1.039	0.308
Social & relational life	0.928	0.335
Professional life	1.913	0.167

(g)

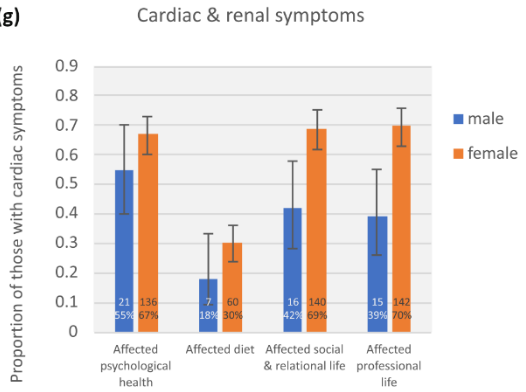

	χ²	p value for gender difference
Psychological health	1.940	0.164
diet	1.977	0.160
Social & relational life	10.115	0.001
Professional life	13.094	<0.001

*Supplementary Figure 30: Impact of symptoms on everyday life (psychological health, diet, social & professional life) by gender continued (e) cognitive symptoms (f) cutaneous & inflammatory symptoms (g) cardiac & renal symptoms. * Note the small number of men. The statistical calculations are unreliable. Error bars are 95% confidence intervals.*

(a)

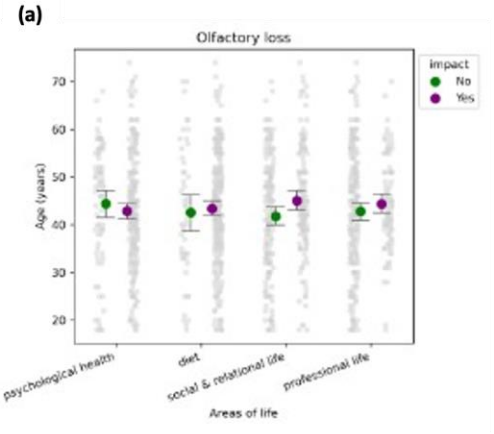

(b)

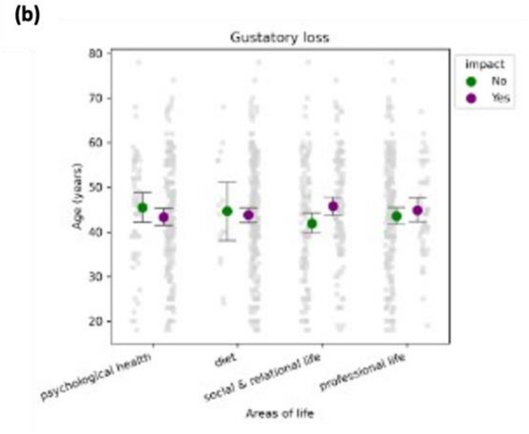

Olfactory loss	t	df	p
Affected psychological health	-0.93	288	0.353
Affected diet	0.42	288	0.673
Affected social & relational life	2.25	288	0.025
Affected professional life	1	288	0.32

Gustatory loss	t	df	p
Affected psychological health	-1.14	238	0.254
Affected diet	-0.27	238	0.791
Affected social & relational life	2.41	238	0.017
Affected professional life	0.65	238	0.517

(c)

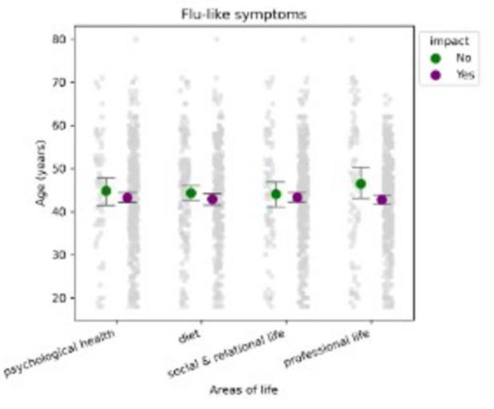

(d)

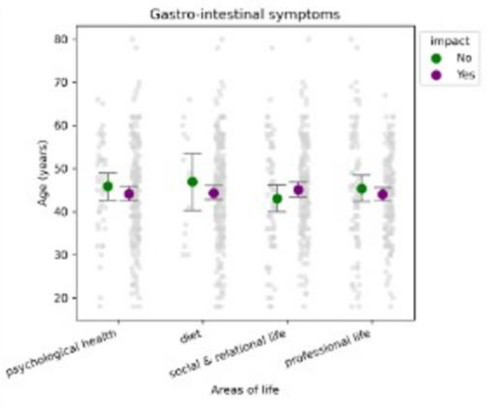

Flu-like symptoms	t	df	p
Affected psychological health	-0.95	475	0.34
Affected diet	-1.24	475	0.217
Affected social & relational life	-0.51	475	0.612
Affected professional life	-2.57	475	0.01

Gastro-intestinal symptoms	t	df	p
Affected psychological health	-0.91	224	0.363
Affected diet	-0.8	224	0.427
Affected social & relational life	1.19	224	0.236
Affected professional life	-0.82	224	0.411

Supplementary Figure 31: Impact of symptoms on everyday life (psychological health, diet, social & professional life) by age. (a) olfactory symptoms (b) gustatory symptoms (c) flu-like symptoms (d) gastro-intestinal symptoms. Error bars are 95% confidence intervals in the mean.

(e)

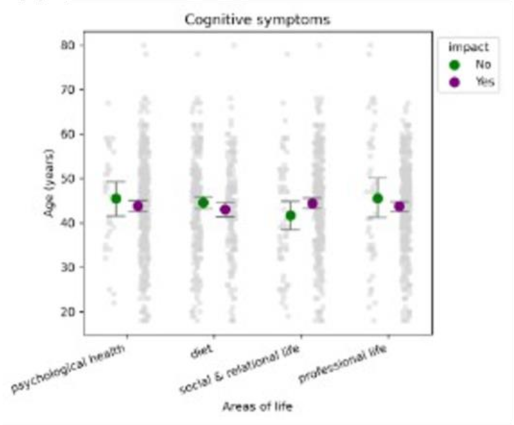

(f)

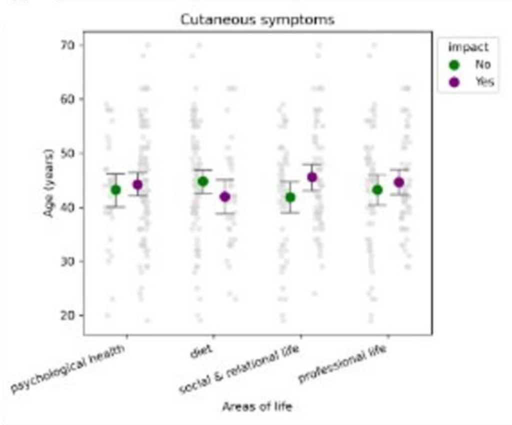

Cognitive symptoms	t	df	p
Affected psychological health	-0.79	380	0.428
Affected diet	-1.28	380	0.202
Affected social & relational life	1.73	380	0.085
Affected professional life	-1.02	380	0.311

Cutaneous symptoms	t	df	p
Affected psychological health	0.47	116	0.64
Affected diet	-1.36	116	0.176
Affected social & relational life	1.96	116	0.053
Affected professional life	0.75	116	0.455

(g)

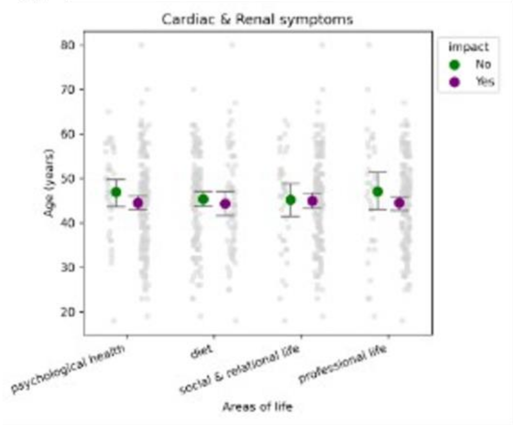

Cardiac symptoms	t	df	p
Affected psychological health	-1.28	193	0.203
Affected diet	-0.64	193	0.526
Affected social & relational life	-0.13	193	0.898
Affected professional life	-1.35	193	0.18

Supplementary Figure 32: Impact of symptoms on everyday life (psychological health, diet, social & professional life) by age continued. (e) cognitive symptoms (f) cutaneous & inflammatory symptoms (g) cardiac & renal symptoms. Error bars are 95% confidence intervals in the mean.

Supplementary Notes 6 (Summary information & verbatim reponses)

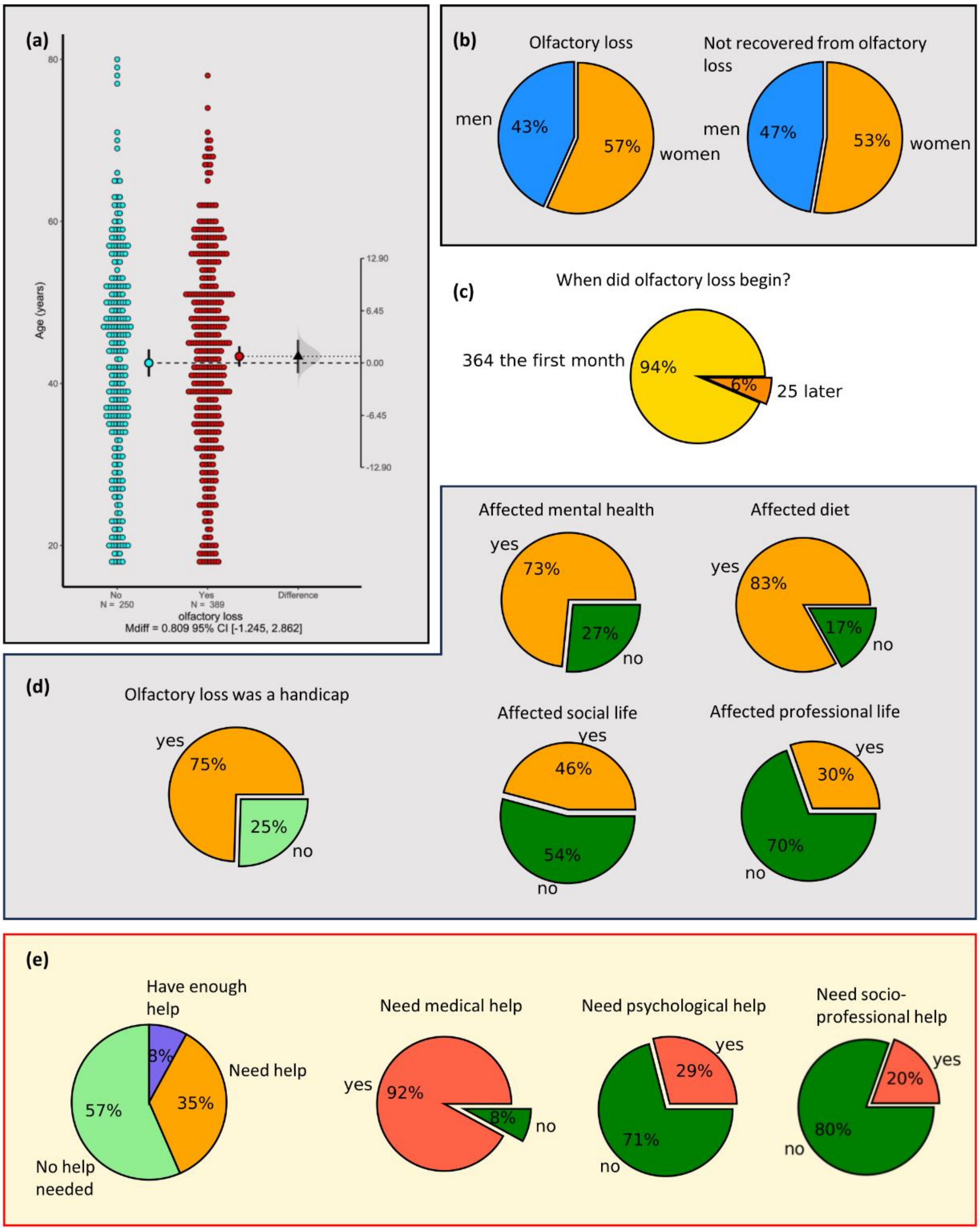

Supplementary Figure 33: Overview of olfactory symptoms for the of the 389 participants. (a) Age distributions of participants; those with olfactory symptoms (red) are older on average than those without, but the difference is not significant at the 95% confidence level. (b) Gender comparisons. Proportions of men (blue) and women (orange) who reported olfactory loss, and the proportions still reporting loss at the time of completing the survey. (c) Reported recovery; note that the times for recovery only represent the proportion of those recovering, many participants had not recovered after six months (d) Proportion of the 389 participants with olfactory loss reporting impact on their psychological well-being, diet, social & relational life, and professional life (e) The needs of those suffering olfactory loss with respect to this symptom (as a proportion of those needing additional help)

Supplementary Table 30: Selected verbatim responses for impact of olfactory loss with English translation

Verbatim response	English translation
je mangeais pour me nourir mais plus par plaisir et je faisais bruler la plupart de mes repas du a mes oublis et je sentais pas que cela brulais	*I ate to feed myself, but no longer for pleasure and I burned most of my meals because of my forgetfulness and I didn't smell what I burned*
totale uniformité des aliments: plus d'odeur plus de saveur, une véritable horreur	*Total uniformity of foodstuffs : no more odour, no more flavour, a real horror*
J'ai arrêté de manger	*I have stopped eating*
perte d'appétit , tendance à trop salé et trop sucré	*loss of appetite, tendency [to season or to eat] too salty and too sweet*
Peur de faire des intoxications alimentaires	*Scared to get food poisoning*
Je ne peux plus manger certains aliments car le goût ressenti n'est plus agréable	*I can't eat certain foods because the taste isn't nice any more*
Je pourrais manger n'importe quoi, tout a ce goût de menthe poivrée qui masque presque tout	*I could eat anything, everything has this peppermint taste that masks almost everything*
Sans l'odeur et le goût, il m'est arrivé de manger des plats qui avaient tourné	*Without smell and taste I have eaten dishes that had gone off*
plus aucune sensation de bien être ou d'être rassasié lors de mon alimentation donc perte d'un besoin de s'alimenter ou d'une sensation de faim	*without smell and taste I no longer have any feeling of well-being or of being satiated when I eat so loss of the need to eat or of a feeling of hunger*
Ne pas savoir ce que l'on sent. Hygiène invérifiable. Oublier une douche après le sport	*Not knowing what you smell. Unverifiable hygiene. Forget a shower after sport*

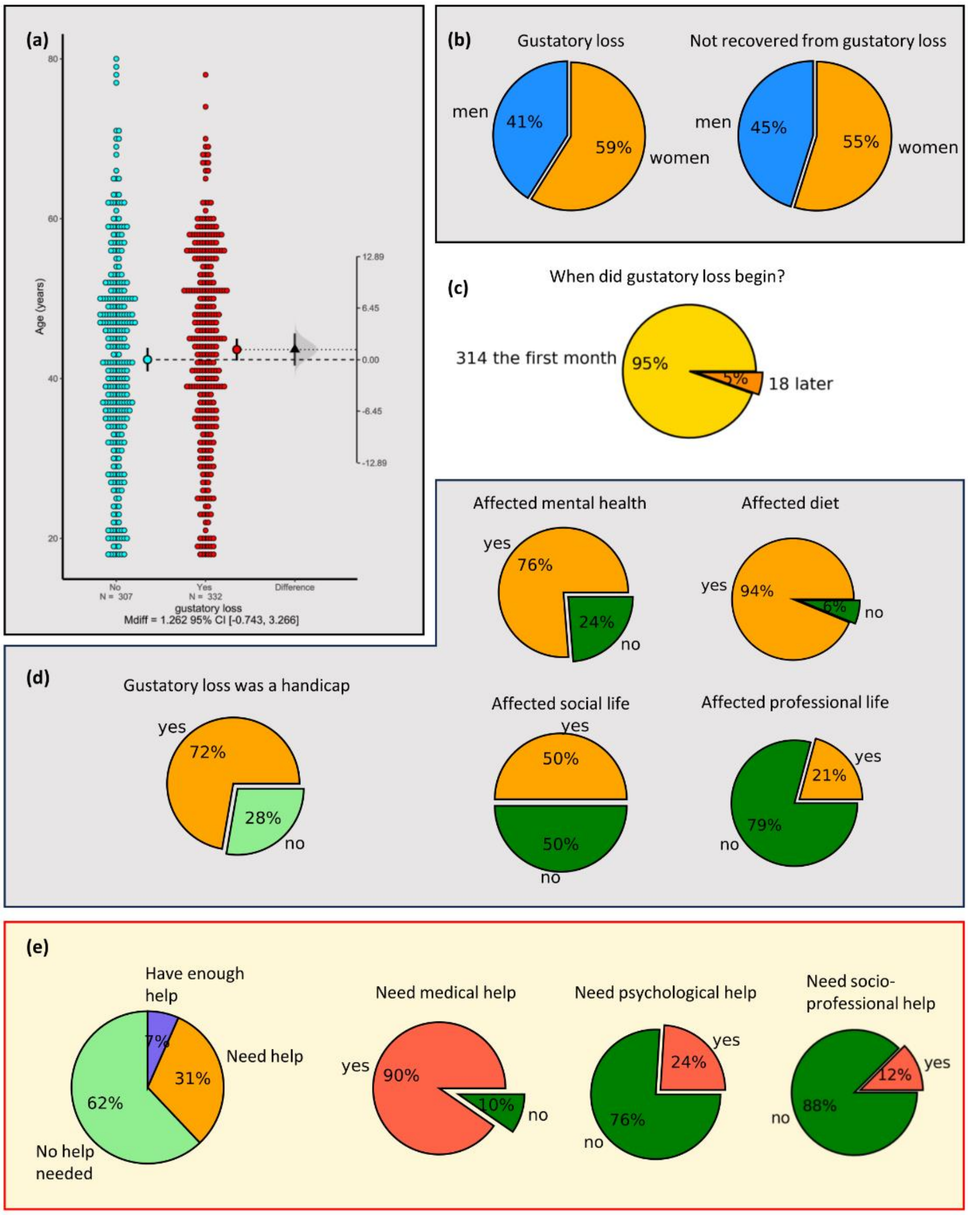

Supplementary Figure 34: Overview of gustatory symptoms for the of the 389 participants. (a) Age distributions of participants; those with gustatory symptoms (red) are older on average than those without, but the difference is not significant at the 95% confidence level (b) Gender comparisons. Proportions of men (blue) and women (orange) who reported gustatory loss, and the proportions still reporting loss at the time of completing the survey. (c) Onset of symptoms (d) Proportion of the 389 participants with gustatory loss reporting impact on their psychological well-being, diet, social & relational life, and professional life (e) The needs of those suffering gustatory loss with respect to this symptom (as a proportion of those needing additional help)

Supplementary Table 31: Selected verbatim responses for impact of gustatory loss with English translation

**Verbatim response**	**English translation**
je me suis nourrie n'importe comment , j'ai testé frénétiquement tout ce que je pouvais tester comme poivre, piments etc dans l'espoir de percevoir au moins une infime partie des saveurs d'avant	*I fed myself anyhow, I frantically tested everything I could test like pepper, chilli peppers etc in the hope of perceiving at least a tiny part of the flavors of before*
Enceinte, plus envie de manger car plus de goût. Perte de poids.	*Pregnant, no desire to eat as no taste. Lost weight.*
pour une cuisinière ,les assaisonnements sont compliqués et ont parfois réservés des surprises pas toujours agréables !!	*for a cook, seasoning is difficult and has sometimes produced surprises not always pleasant !!*
dégout alimentaire pour certains aliments que je mangeais avant	*Dislike of certain foods that I used to eat*
plus de plaisir de cuisiner ou même d'aller au restaurant	*no more pleasure in cooking or even going to a restaurant*
Perte du goût surtout pour les produits naturels (exemple : fraises du jardin), alors que le gout de fraise est présent dans les produits artificiels (yaourts aromatisés). J'achète plus de produits transformés et moins de produits naturels	*Loss of taste especially for natural products (example: strawberries from the garden), while the strawberry taste is present in artificial products (flavored yoghurts).*<br>*I buy more processed products and fewer natural products*
Pas envie de manger alors qu'il le faut	*Don't want to eat when one must*
manger est plus un plaisir , mais une obligation !	*Eating is no longer a pleasure, but an obligation!*
Ajout de sel ou d'épice ou ajout de sucre	*Addition of salt, or spice or addition of sugar*
moins d'envie de manger malgré la faim ressentie, parce que tout était fade, j'ajoutais plus d'épices, sel, sucre... qu'avant	*less desire to eat despite feeling hungry, because everything was bland, I added more spices, salt, sugar... than before*

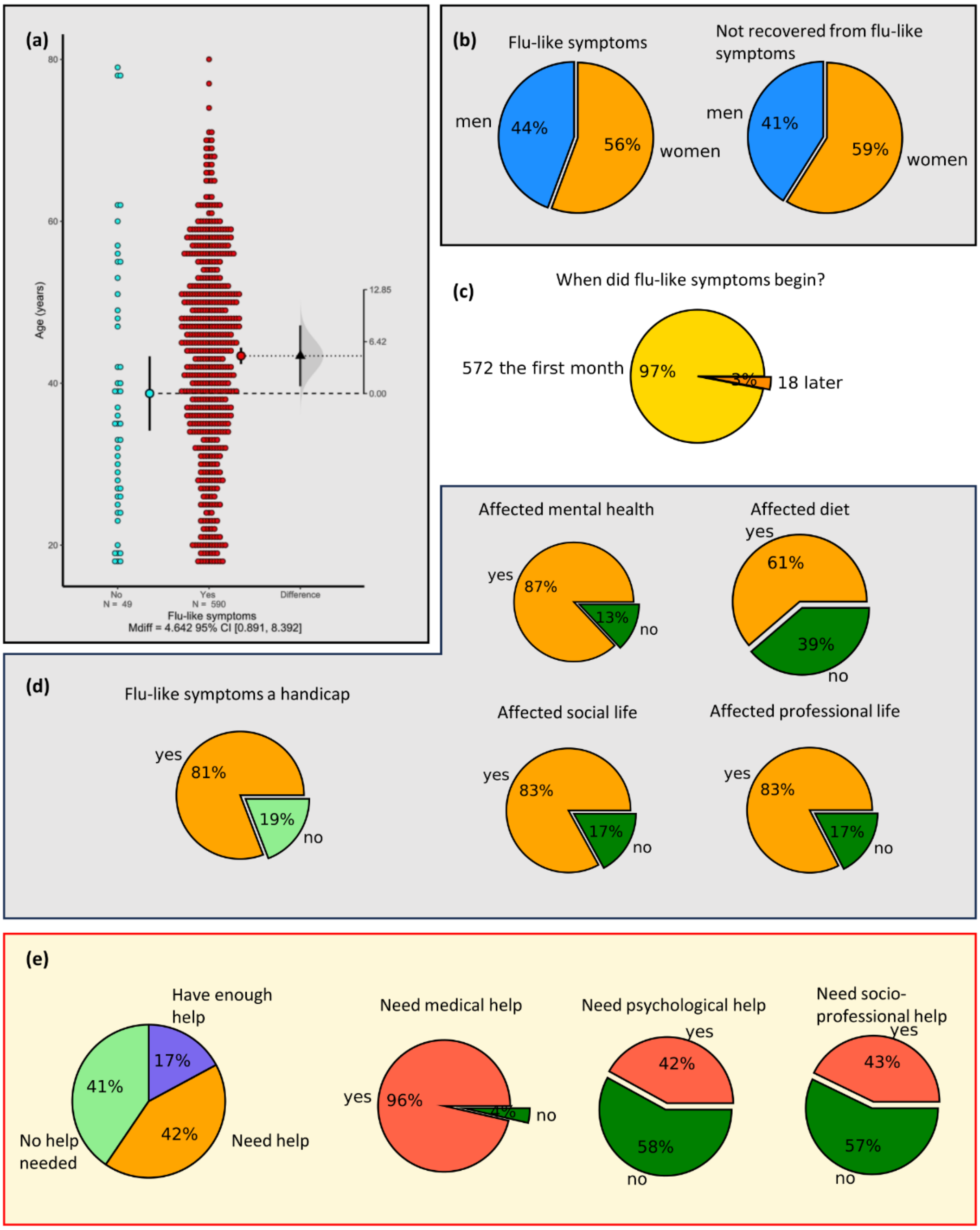

Supplementary Figure 35: Overview of flu-like symptoms for the of the 389 participants. (a) Age distributions of participants; those with flu-like symptoms (red) are older on average than those without. (b) Gender comparisons. Proportions of men (blue) and women (orange) who reported flu-like symptoms, and the proportions still reporting symptoms at the time of completing the survey. (c) Onset of symptoms (d) Proportion of the 389 participants with flu-like symptoms reporting impact on their psychological well-being, diet, social & relational life, and professional life (e) The needs of those suffering flu-like symptoms with respect to this symptom (as a proportion of those needing additional help)

Supplementary Table 32: Selected verbatim responses for impact of flu-like symptoms with English translation

**Verbatim response**	**English translation**
fatigue je dormais 19h/24 mal de gorge , essouflement (genre asthme) ,tachycardie a l'effort , marche rapide ou montée d'escalier	*tiredness I slept 19hrs/24hrs sore throat, breathlessness (like asthma) tachycardia induced by any effort, quick walking or going up stairs.*
Fatigue, migraine, essouflement qui ont duré 7 mois	*Tiredness, migraine, breathlessnes that has lasted 7 months*
Cloué au lit durant 6 jours. Ensuite, coups de fatigue extrême nécessitant de m'allonger et de dormir, de moins en moins fréquents au cours des deux mois suivants les premiers symptomes	*Bedridden for 6 days. After that bouts of extreme tiredness that meant I had to lie down and sleep. Les and less often over the 2 months following the first symptoms*
etat febrile et fatigue soudaine qq jours puis ça disparaissait et revenait ensuite : pendant 6 mois environ	*feverish and sudden exhaustion a few days then that disappeared and came back again : for 6 months*

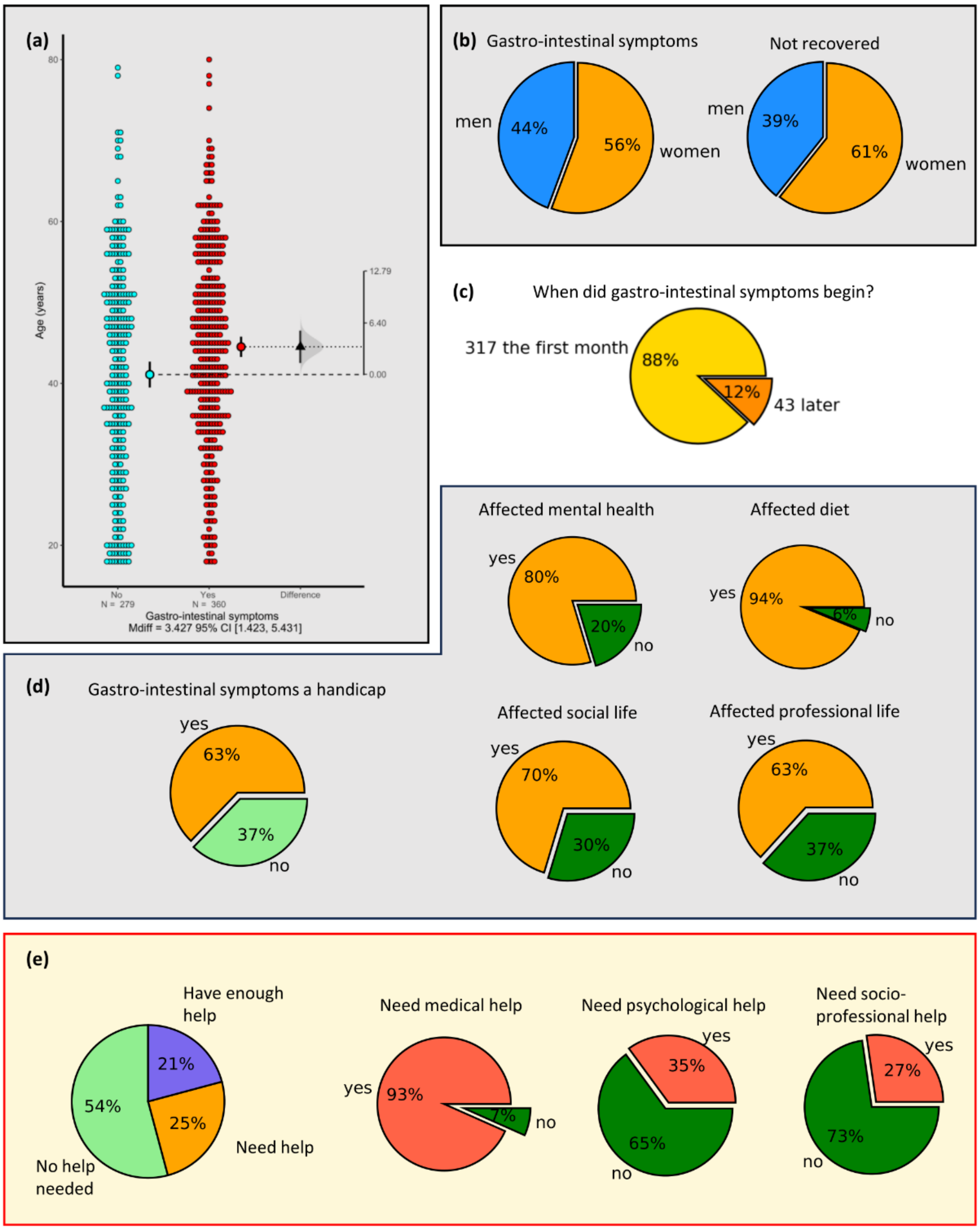

Supplementary Figure 36: Overview of gastro-intestinal symptoms for the of the 389 participants. (a) Age distributions of participants; those with gastro-intestinal symptoms (red) are older on average than those without. (b) Gender comparisons. Proportions of men (blue) and women (orange) who reported gastro-intestinal symptoms, and the proportions still reporting symptoms at the time of completing the survey. (c) Onset of symptoms (d) Proportion of the 389 participants with gastro-intestinal symptoms reporting impact on their psychological well-being, diet, social & relational life, and professional life (e) The needs of those suffering gastro-intestinal symptoms with respect to this symptom (as a proportion of those needing additional help)

Supplementary Table 33: Selected verbatim responses for impact of gastro-intestinal symptoms with English translation

**Verbatim response**	**English translation**
Impossible de manger normalement ou de sortir	*Impossible to eat normally or to go out*
nausées, vomissements, diarrhées	*nausea, vomiting, diarrhea*
Pour tout le quotidien... épuisant, douleurs…	*For all everyday life… exhausting, pain…*
Je me suis étouffée deux fois, j'ai maigri de 5 kilos en deux mois (ce dernier symptôme revient)	*I choked myself twice, I lost 5kg in two months (this last symptom is coming back)*
Trouver des WC en urgence	*Find toilets urgently*

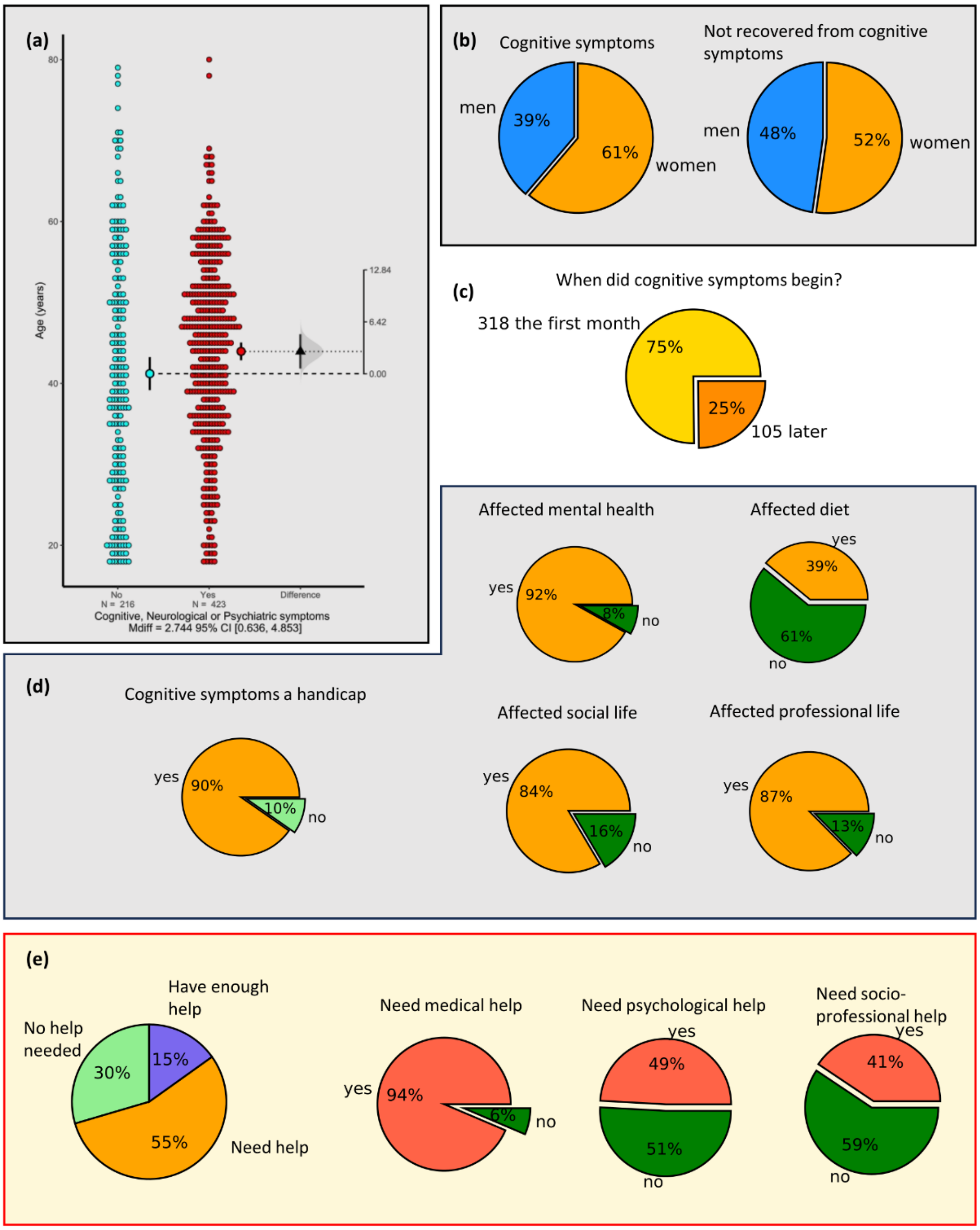

Supplementary Figure 37: Overview of cognitive, neurological and psychiatric symptoms for the of the 389 participants. (a) Age distributions of participants; those with cognitive, neurological and psychiatric symptoms (red) are older on average than those without. (b) Gender comparisons. Proportions of men (blue) and women (orange) who reported cognitive, neurological and psychiatric symptoms, and the proportions still reporting symptoms at the time of completing the survey. (c) Onset of symptoms (d) Proportion of the 389 participants with cognitive, neurological and psychiatric symptoms reporting impact on their psychological well-being, diet, social & relational life, and professional life (e) The needs of those suffering cognitive, neurological and psychiatric symptoms with respect to this symptom (as a proportion of those needing additional help)

Supplementary Table 34: Selected verbatim responses for impact of cognitive symptoms with English translation

Verbatim response	English translation
le sommeil oui car certains jours je ne peut remplir mes obligations en raisons de nuits perturbées. Difficultés à reprendre sereinement des activités sociales ou relationnelles je vis tout cela comme un traumatisme (je fais des cauchemars du covid et je me suis sentie en panique dans des moments de convivialités entre amis) , je ne me sens pas comprise, et les troubles de mémoire et d'attention m'angoissent (j'ai fait bruler des choses par exemple en oubliant que c'était sur le feu ou en laissant des casseroles vides sur le gaz. je ne me rappelle plus si j'ai pris mon traitement, peur de le prendre deux fois par erreur ou de faire la vaisselle avec un autre détergent qui se trouve à proximité (je me surprend à faire les mauvais gestes dans le cadre d'une action déterminée)	*sleep yes because some days I cannot fulfill my obligations due to disturbed nights. Difficulties in tranquilly resuming social or relational activities. I experience all of this as a trauma (I have nightmares of covid and I felt panicked in moments of conviviality between friends), I do not feel understood, and the problems of memory and attention worry me (I burned things for example by forgetting that it was on the stove or by leaving empty pans on the gas. I no longer remember if I took my medicine, I'm afraid of taking it twice by mistake or doing the dishes with another detergent that is nearby (I find myself doing the wrong things as part of a specific action)*
fatigue extrême celle qui vous tire vers votre lit avec une somnolence . Le sommeil n'est pas réparateur avec des insomnies terribles même qd très fatiguée. il y a aussi cette prise de poids incroyable pour la 1ere fois de ma vie Je ne peux plus plus avoir tout en tête comme avant pendant des conversations j oublie des points je n' ai plus cette faculté a resynthetiser une conversation soutenue c'est plus compliqué	*extreme fatigue the kind that pulls you to your bed with drowsiness. Sleep is not restorative with terrible insomnia even when very tired. there is also this incredible weight gain for the first time in my life. I can no longer have everything in my mind as before during conversations. I forget points. I no longer have this ability to resynthesise a sustained conversation it's more complicated*
Incapacité à conduire et à réfléchir pour mon travail	*Inability to drive and to think for my job*
fonctionnement à 2 à l'heure au travail	*running at 2 an hour at work*
Migraine, troubles de la mémoire, brouillard mental (horrible à vivre), trouble de l'attention (difficulté pour conduire)	*Migraine, memory problems, mental fog (horrible to live with), attention disorder (difficulty driving)*

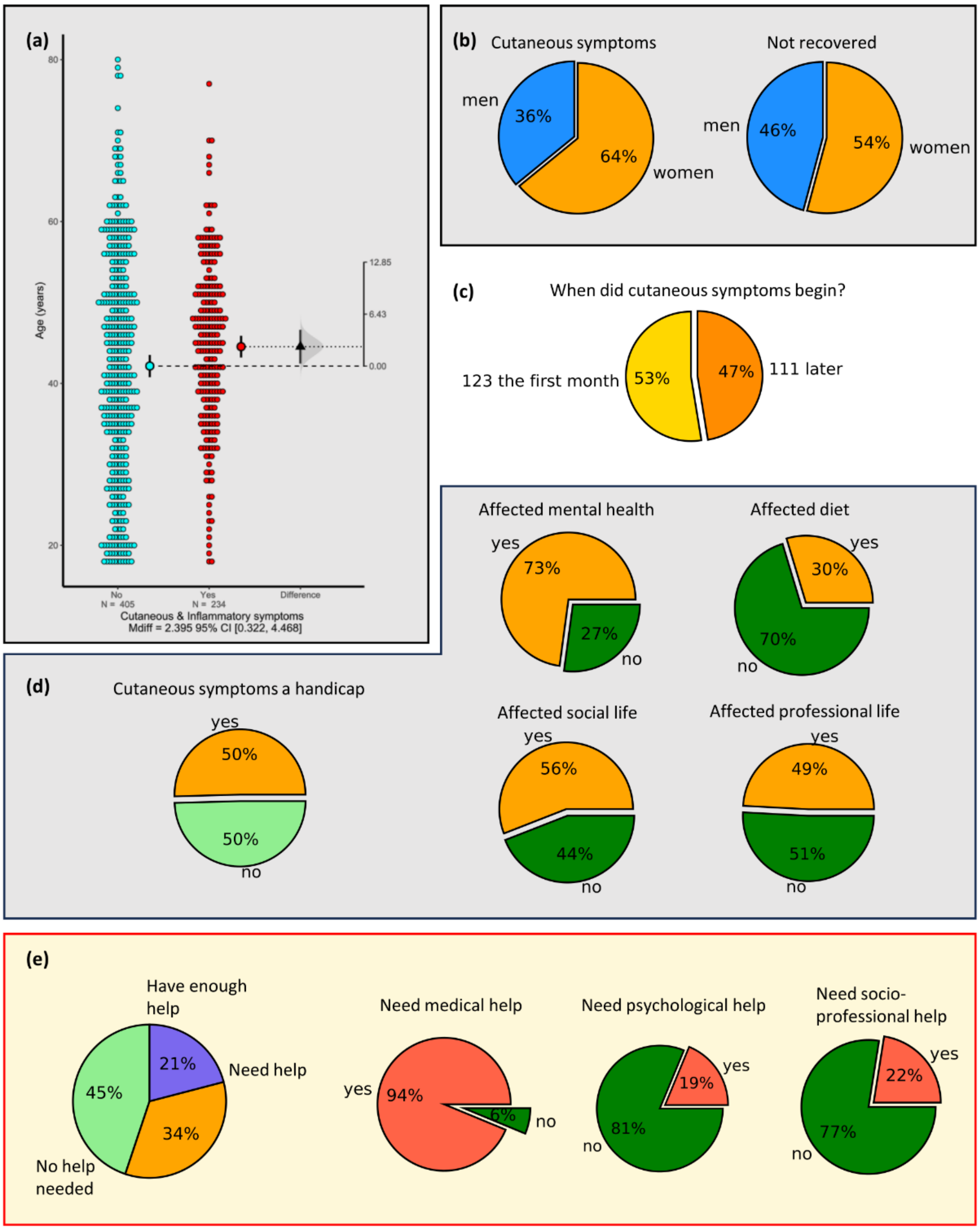

Supplementary Figure 38: Overview of cutaneous & inflammatory symptoms for the of the 389 participants. (a) Age distributions of participants; those with cutaneous & inflammatory symptoms (red) are older on average than those without. (b) Gender comparisons. Proportions of men (blue) and women (orange) who reported cutaneous & inflammatory symptoms and the proportions not recovered from these symptoms at the time of completing the survey. (c) Onset of symptoms (d) Proportion of the 389 participants with cutaneous & inflammatory symptoms reporting impact on their psychological well-being, diet, social & relational life, and professional life (e) The needs of those suffering cutaneous & inflammatory symptoms with respect to this symptom (as a proportion of those needing additional help)

Supplementary Table 35: Selected verbatim responses for impact of cutaneous and inflammatory symptoms with English translation

**Verbatim response**	**English translation**
Douleurs dues aux engelures aux pieds	*Pain from chilblain in the feet*
Démangeaisons plaques troubles dermatologiques	*itchy plaques and dermatological problems*
Difficultés de faire des activités	*Difficulties doing activities*
Perte de cheveux et démangeaisons	*Hair loss and itching*
Sensation de brûlures dans le dos	*Burning sensation on my back*
problème d'yeux , par moment je porte des lunettes de soleil dans la maison, ou les yeux brulent difficiles à ouvrir et problème peau gratte au cou et visage, ou dans le dos	*problem with eyes, sometimes I wear sunglasses in the house, or the eyes burn difficult to open and problem with itchy skin on the neck and face or on the back*
« Handicapants » car l'apparence est modifiée	*"handicapping" as the appearance is altered*
Pas tres agréable de perdre ses cheveux	*Not very nice to lose your hair*
Démangeaisons épouvantables pendant 3 mois sur le tronc	*Terrible itching for 3 months on the trunk*

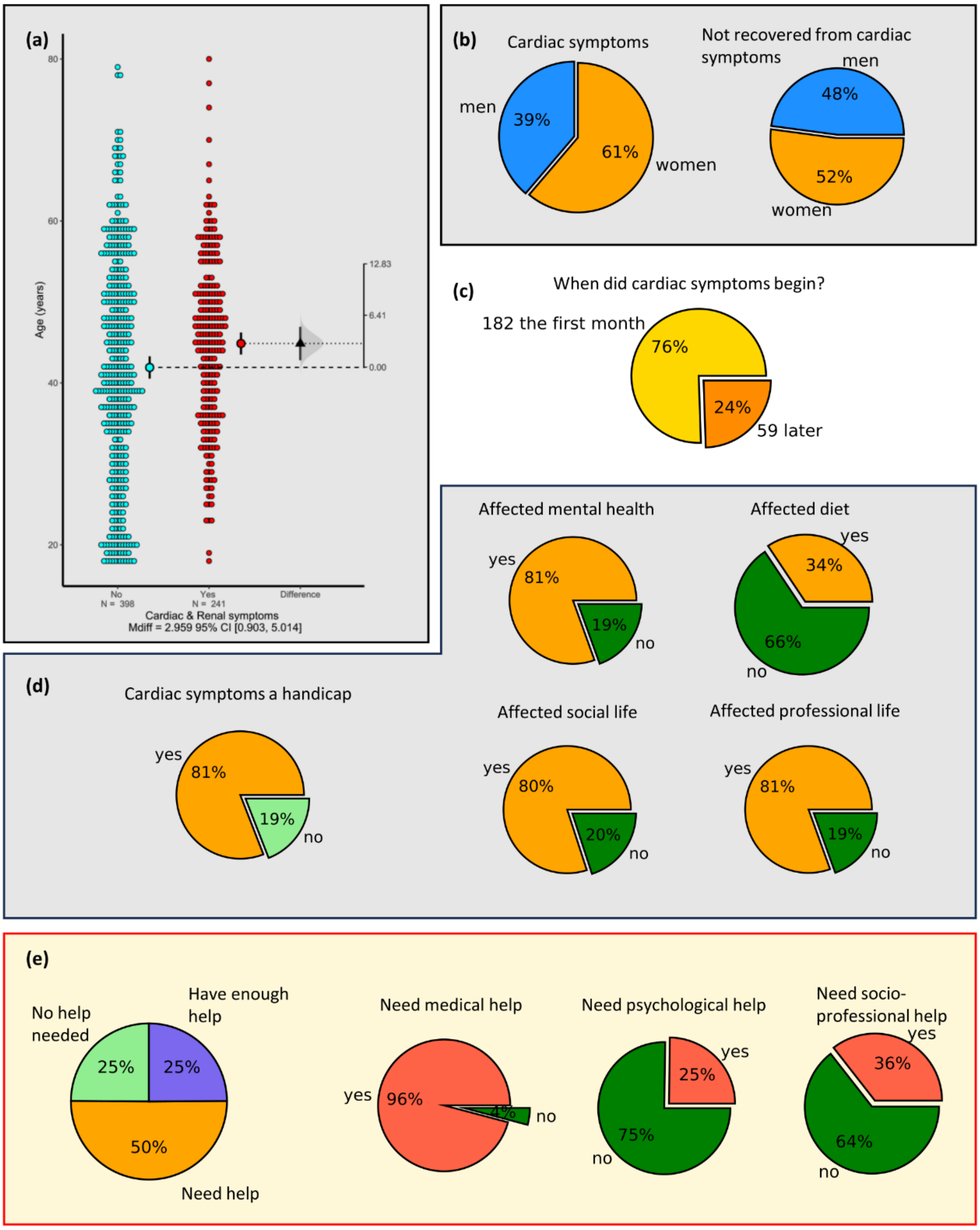

Supplementary Figure 39: Overview of cardiac & renal symptoms for the of the 389 participants. (a) Age distributions of participants; those with cardiac & renal symptoms (red) are older on average than those without. (b) Gender comparisons. Proportions of men (blue) and women (orange) who reported cardiac & renal symptoms, and the proportions still reporting symptoms at the time of completing the survey. (c) Onset of symptoms (d) Proportion of the 389 participants with cardiac & renal symptoms reporting impact on their psychological well-being, diet, social & relational life, and professional life (e) The needs of those suffering cardiac & renal symptoms, with respect to this symptom (as a proportion of those needing additional help)

Supplementary Table 36: Selected verbatim responses for impact of cardiac or renal symptoms with English translation

**Verbatim response**	**English translation**
Douleurs thoraciques	*chest pain*
Mal au coeur, battements trop rapides, surtout au réveil ce qui entraînait des difficultés pour me lever	*Heart pain, beating too fast, especially when waking up which made it difficult for me to get up*
Au moindre effort je faisais de la tachycardie	*At the slightest effort I had tachycardia*
plus génant lors de sortie que je raccourcis car je rentre vite de peur de faire un malaise. c'est douloureux et rien ne fonctionne en médicament.	*more annoying when going out that I shorten because I come back quickly for fear of fainting. it's painful and no medicine works.*
douleurs intercostales si importantes que j'ai cru 3 fois faire un infarctus: 3 passages aux urgences d'avril 2020 à jui 2020: tension aux alentours de 280/140 a deux fois	*intercostal pain so severe that 3 times I thought I was having a heart attack: 3 visits to A&E between April 2020 and June 2020: blood pressure around 280/140 twice*
incapacité à faire de l'exercice dans ce cas, fatigue, angoisse	*inability to exercise in this case, fatigue, anxiety*
douleurs à la poitrine récurrentes avec essoufflement, troubles cardiaques (tachycardie pour des efforts ridicules), mais pas d'examens parce qu'il "faut que je me déteeeende", et que "je suis anxieeeuse"	*recurrent chest pain with breathlessness, cardiac problems (tachycardia for ridiculously small efforts) but no examinations because I "need to relaaaax" and that "I am anxiooous"*
Les douleurs sont intenses	*the pain is intense*

Supplementary References

1. de Bellefon, M.-P., Eusebio, P., Forest, J. & Warnod, R. 38% de la population française vit dans une commune densément peuplée. *Statistiques et études* https://www.insee.fr/fr/statistiques/4252859 (2019).
2. INSEE. Population statistics of France. https://www.insee.fr/fr/statistiques/6036447 (2021).
