# Supplementary material for: Identification of the needs of individuals affected by COVID-19
Source: Commun Med (Lond). 2024 May 9;4:83. doi: 10.1038/s43856-024-00510-1 (PMC11082167; doi:10.1038/s43856-024-00510-1)
Supplement: Supplementary file 2 — Reporting Summary [file 43856_2024_510_MOESM2_ESM.pdf]

nature portfolio

Corresponding author(s): DBPR; COMMSMED-23-0404B

Last updated by author(s): Mar 4, 2024

Reporting Summary

Nature Portfolio wishes to improve the reproducibility of the work that we publish. This form provides structure for consistency and transparency in reporting. For further information on Nature Portfolio policies, see our Editorial Policies and the Editorial Policy Checklist.

Statistics

For all statistical analyses, confirm that the following items are present in the figure legend, table legend, main text, or Methods section.

n/a	Confirmed	
☐	☒	The exact sample size (*n*) for each experimental group/condition, given as a discrete number and unit of measurement
☐	☒	A statement on whether measurements were taken from distinct samples or whether the same sample was measured repeatedly
☐	☒	The statistical test(s) used AND whether they are one- or two-sided<br>*Only common tests should be described solely by name; describe more complex techniques in the Methods section.*
☐	☒	A description of all covariates tested
☐	☒	A description of any assumptions or corrections, such as tests of normality and adjustment for multiple comparisons
☐	☒	A full description of the statistical parameters including central tendency (e.g. means) or other basic estimates (e.g. regression coefficient) AND variation (e.g. standard deviation) or associated estimates of uncertainty (e.g. confidence intervals)
☐	☒	For null hypothesis testing, the test statistic (e.g. F, t, r) with confidence intervals, effect sizes, degrees of freedom and P value noted<br>*Give P values as exact values whenever suitable.*
☒	☐	For Bayesian analysis, information on the choice of priors and Markov chain Monte Carlo settings
☒	☐	For hierarchical and complex designs, identification of the appropriate level for tests and full reporting of outcomes
☐	☒	Estimates of effect sizes (e.g. Cohen's *d*, Pearson's *r*), indicating how they were calculated

Our web collection on statistics for biologists contains articles on many of the points above.

Software and code

Policy information about availability of computer code

Data collection	Lime survey (csv format)
Data analysis	jamovi

For manuscripts utilizing custom algorithms or software that are central to the research but not yet described in published literature, software must be made available to editors and reviewers. We strongly encourage code deposition in a community repository (e.g. GitHub). See the Nature Portfolio guidelines for submitting code & software for further information.

Data

Policy information about availability of data

All manuscripts must include a data availability statement. This statement should provide the following information, where applicable:

- Accession codes, unique identifiers, or web links for publicly available datasets
- A description of any restrictions on data availability
- For clinical datasets or third party data, please ensure that the statement adheres to our policy

the data are publicly available on Zenodo (DOI: 10.5281/zenodo.7920303).

Human research participants

Policy information about studies involving human research participants and Sex and Gender in Research.

Reporting on sex and gender	Participants self-reported their gender. Gender effects were considered wherever possible.
Population characteristics	The people were mostly of working age (75th percentile is 52 years) and predominantly (77%) female. The majority experienced mild initial illness. Only 42 people were hospitalized (7%), of whom 25 received oxygen and 8 were treated in intensive care. 110 people (17%) were either occasional or regular smokers, which compares to 18.5% of people over 18 years old in France
Recruitment	Participants had access to the questionnaire on the website https://project.crnl.fr/covid/. They learned about the survey through their internet searches and we also distributed the link to our scientific and academic network, and to the communication officers of the French institutions involved in the project. Our data are limited to those people who chose to respond and to complete a long questionnaire. Our survey population has a preponderance of women and a large number of people with "long COVID" (this appears typical for this kind of survey). We also over-represent urban, educated individuals and exclude those with no internet access. We can assume a selection bias towards people motivated to seek assistance with symptoms that are problematic for them. There is also the possibility that people who chose to respond are especially health sensitive. The large proportion of the survey population needing help with their symptoms may not, therefore, be reflected in the general population.
Ethics oversight	The study was approved by the Institutional Review Board of INSERM (IRB00003888, IORG0003254, FWA00005831) of the French Institute of medical research and health, under number 21-805.

Note that full information on the approval of the study protocol must also be provided in the manuscript.

Field-specific reporting

Please select the one below that is the best fit for your research. If you are not sure, read the appropriate sections before making your selection.

☐ Life sciences ☒ Behavioural & social sciences ☐ Ecological, evolutionary & environmental sciences

For a reference copy of the document with all sections, see nature.com/documents/nr-reporting-summary-flat.pdf

Behavioural & social sciences study design

All studies must disclose on these points even when the disclosure is negative.

Study description	The study consisted of a cross-sectional online participatory survey collecting both qualitative and quantitative data.
Research sample	639 adults (over 18 years old), 77% female, resident in France, who completed the entire questionnaire, were answering the questionnaire for the first time, completed the entire survey, and either declared having been diagnosed COVID+ by an analytical test (PCR, lateral flow, blood test etc.) or having been diagnosed COVID+ by a doctor on the basis of their symptoms alone.
Sampling strategy	It was difficult to estimate sample size a priori. Volunteers learned about the survey through their Internet searches and via our distribution network (scientists, academics, communication officers), and the survey was able to cover the waves of infection in France until approximately the start of the omicron wave, providing a total of 639 participants. All planned analyses were carried out. Some exploratory analyses in sub-groups of subjects (e.g. according to Covid variants) could not be carried out because the sample size was too small.
Data collection	LimeSurvey, a tool that creates online questionnaires and surveys, was used to collect the data. The raw data was first exported from LimeSurvey in xlsx format The participants were unsupervised and data were totally anonymous
Timing	Data were collected between 15th July 2021 and 6th September 2022
Data exclusions	Exclusion criteria were: persons under the age of 18 years, those without a diagnosis of COVID-19 (made either via an analytical test or by a doctor on the basis of symptoms alone), those who failed to complete the entire survey, or attempted to fill it in for a second time, those not resident in France and those who provided implausible diagnosis dates. These criteria excluded 415 of the 1054 participants
Non-participation	not applicable
Randomization	not applicable

Reporting for specific materials, systems and methods

We require information from authors about some types of materials, experimental systems and methods used in many studies. Here, indicate whether each material, system or method listed is relevant to your study. If you are not sure if a list item applies to your research, read the appropriate section before selecting a response.

Materials & experimental systems

n/a	Involved in the study
☒	☐ Antibodies
☒	☐ Eukaryotic cell lines
☒	☐ Palaeontology and archaeology
☒	☐ Animals and other organisms
☒	☐ Clinical data
☒	☐ Dual use research of concern

Methods

n/a	Involved in the study
☒	☐ ChIP-seq
☒	☐ Flow cytometry
☒	☐ MRI-based neuroimaging
